# Cobalt-Catalyzed Four-Component Carbonylative Synthesis of γ‑Thioesters and γ‑Thioamides from Thioethers and Ethylene

**DOI:** 10.1021/acs.orglett.5c04357

**Published:** 2025-11-07

**Authors:** Xudong Mao, Le-Cheng Wang, Xiao-Feng Wu

**Affiliations:** † Dalian National Laboratory for Clean Energy, Dalian Institute of Chemical Physics, Chinese Academy of Sciences, Dalian 116023, China; ‡ Leibniz-Institut für Katalyse e. V., Albert-Einstein-Straβe 29a, 18059 Rostock, Germany

## Abstract

Multicomponent carbonylative reactions have proven to be indispensable for constructing heterocycles and highly functionalized molecular frameworks and represent an attractive strategy for alkene functionalization. Herein, we report a cobalt-catalyzed four-component carbonylation of thioethers and ethylene gas with diverse nucleophiles, affording a series of γ-thioether amides and γ-thioether esters. The reaction features a broad substrate scope, good chemo- and regioselectivity, and excellent functional-group tolerance.

## Graphical abstract

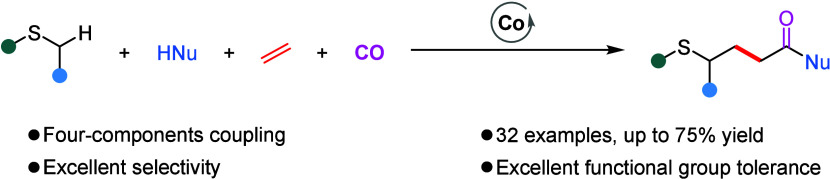

Sulfur-containing compounds (thioethers, sulfoxides, sulfones, and others) are widely distributed in pharmaceuticals, natural products, materials, and agrochemicals.^1^ Accordingly, the synthesis and functionalization of sulfur-containing compounds have gained significant attention in recent years.^2^ In particular, direct functionalization of the C–H bond at the α-position of sulfur has thus been recognized as a valuable method for accessing structurally diverse sulfur-containing compounds. Unsurprisingly, significant advances have been made in the α-C–H functionalization of thioethers during the past decade through photocatalysis, transition metal catalysis, or the use of stoichiometric oxidants (Scheme 1a).^3^

**Scheme 1:**
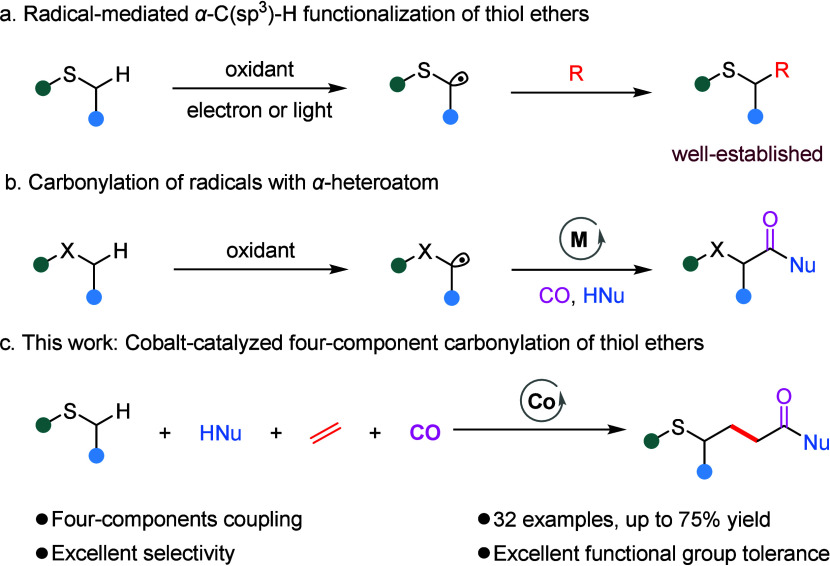
Catalytic Carbonylation of Thioethers

Ethylene is a widely used industrial feedstock, primarily for the production of bulk chemicals such as ethylene oxide and acetaldehyde.^4^ However, its application in modern synthetic methodology remains limited, largely due to its gaseous nature and structural simplicity. Nevertheless, as a readily available C2 source, ethylene represents a highly attractive synthon for molecular framework extension.^5^ On the other hand, the transition metal-catalyzed carbonylative reaction is a class of potent transformations as it involves CO as the C1 source. Besides, carbonylation reactions provide an efficient and atom-economical route to access a wide array of carbonyl-containing compounds.^6^ Multicomponent carbonylation reactions involving ethylene and carbon monoxide under transition metal catalysis have been explored in recent years. A tandem carbonylation reaction between thioethers and ethylene offers a promising approach for the synthesis and derivatization of such compounds. Recently, numerous impressive examples of carbonylative reactions that have four or more components have been reported as particularly challenging synthetic strategies,^7^ including transition metal-catalyzed carbonylative transformation of α-heteroatom-substituted carbon radicals with various nucleophiles (Scheme 1b).^8^ Nevertheless, strategies based on the α-C–H activation of thioethers have rarely been reported. On the other hand, γ-carbonylated thioethers have been broadly applied in the pharmaceutical industry.^9^ Multicomponent carbonylation reactions of sulfur-containing compounds have attracted more attention due to their unique biological compounds, but transition metal catalysis still faces significant challenges. (1) Sulfur, carbon monoxide, and ethylene can all coordinate to the metal center, influencing the catalytic environment. (2) Activation of thioethers to radicals renders highly reactive compounds, frequently giving rise to various byproducts, such as sulfur atom oxidation. In this work, we report a cobalt-catalyzed four-component carbonylation of thioethers and ethylene, with alcohols and amines as the nucleophiles, affording a series of γ-carbonylated thioether derivatives in moderate to good yields (Scheme 1c).

Inspired by our previous studies,^10^ we chose tetrahydrothiophene (**1a**), aniline (**2a**), and ethylene gas as model substrates to explore the optimal conditions for this four-component carbonylation (Table 1). Through systematic screening, employing Co­(acac)_2_ as the catalyst and DTBP as the oxidant in the presence of 10 bar of ethylene gas and 50 bar of carbon monoxide at 120  °C, the desired product **3a** was isolated in 67% yield (entry 1). Control experiments revealed that both the cobalt catalyst and the oxidant are essential for this reaction and the desired carbonylation product could be obtained in 40% yield without any additional ligand (entries 2–4). Further evaluation of the cobalt catalyst showed that Co­(acac)_3_ or Co­(OAc)_2_ gave lower yields while CoCl_2_ and CoBr_2_ failed to afford the desired product. Other transition metals, including Fe­(acac)_3_, Ni­(acac)_2_, and Cu­(acac)_2_, were entirely ineffective, while PdCl_2_ provided the target product in 43% yield, without generating direct carbonyl insertion byproduct **3a′**. We next examined a series of bipyridine ligands with different electronic substituents (**L2**–**L4**) and 1,10-phenanthroline, which all resulted in lower yields (entries 12–15). Moreover, all tested peroxides failed to promote the reaction, underscoring the high sensitivity of the system to the identity of the oxidant (entries 16 and 17).

**Table 1:**
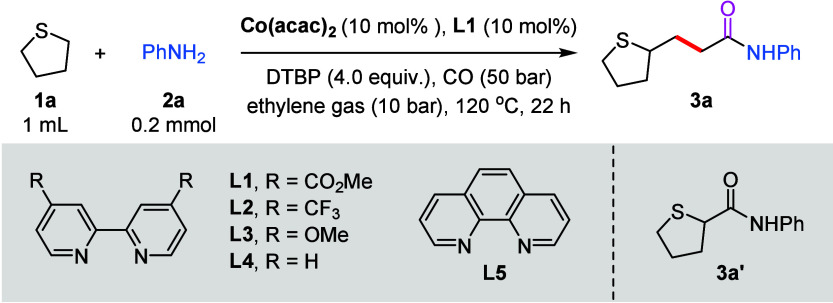
Optimization of the Reaction Conditions^a^,^b^

entry	variation from the standard conditions	yield of **3a** (%)^c^
1	none	70 (67)
2	no Co(acac)_2_	0
3	no ligand	40
4	no DTBP	0
5	Co(acac)_3_ instead of Co(acac)_2_	62
6	Co(OAc)_2_ instead of Co(acac)_2_	31
7	CoCl_2_ or CoBr_2_ instead of Co(acac)_2_	0
8	Fe(acac)_3_ instead of Co(acac)_2_	0
9	Ni(acac)_2_ instead of Co(acac)_2_	0
10	Cu(acac)_2_ instead of Co(acac)_2_	0
11	PdCl_2_ instead of Co(acac)_2_	21
12	**L2** instead of **L1**	52
13	**L3** instead of **L1**	56
14	**L4** instead of **L1**	50
15	**L5** instead of **L1**	61
16	TBPB, BPO, or H_2_O_2_ instead of DTBP	0
17	TBHP or *m*-CPBA instead of DTBP	0

a Reaction conditions: **1a** (1.0 mL), **2a** (0.2 mmol, 1.0 equiv), Co­(acac)_2_ (10 mol %), **L1** (10 mol %), DTBP (4.0 equiv), CO (50 bar), ethylene gas (10 bar), 120 °C, 22 h. DTBP = di-*tert*-butyl peroxide. TBPB = *tert*-butyl peroxybenzoate. BPO = benzoyl peroxide. TBHP = *tert*-butyl hydroperoxide. *m*-CPBA = 3-chloroperbenzoic acid.

b Yields were determined by GC analysis using hexadecane as an internal standard.

c Isolated yield.

Building upon the optimized conditions, we next explored the substrate scope to evaluate the functional-group tolerance of this four-component carbonylation (Scheme 2). A range of nucleophiles, including amines, alcohols, and phenols, were investigated. Initially, *para*-monosubstituted anilines bearing either electron-donating groups (Me or OMe), electron-withdrawing groups (CF_3_ or CO_2_Me), or halogens (F, Cl, or Br) were well tolerated, affording the corresponding γ-amido thioesters in moderate to good yields (**3a**–**3i**). Similarly, a *m*-methoxy-substituted aniline (**3j**) also amenable to this transformation provided the corresponding carbonylation product in 67% yield. Similarly, heteroaryl amines were also compatible under the standard conditions, providing the desired products in 60–72% yields (**3k**–**3m**).

**Scheme 2:**
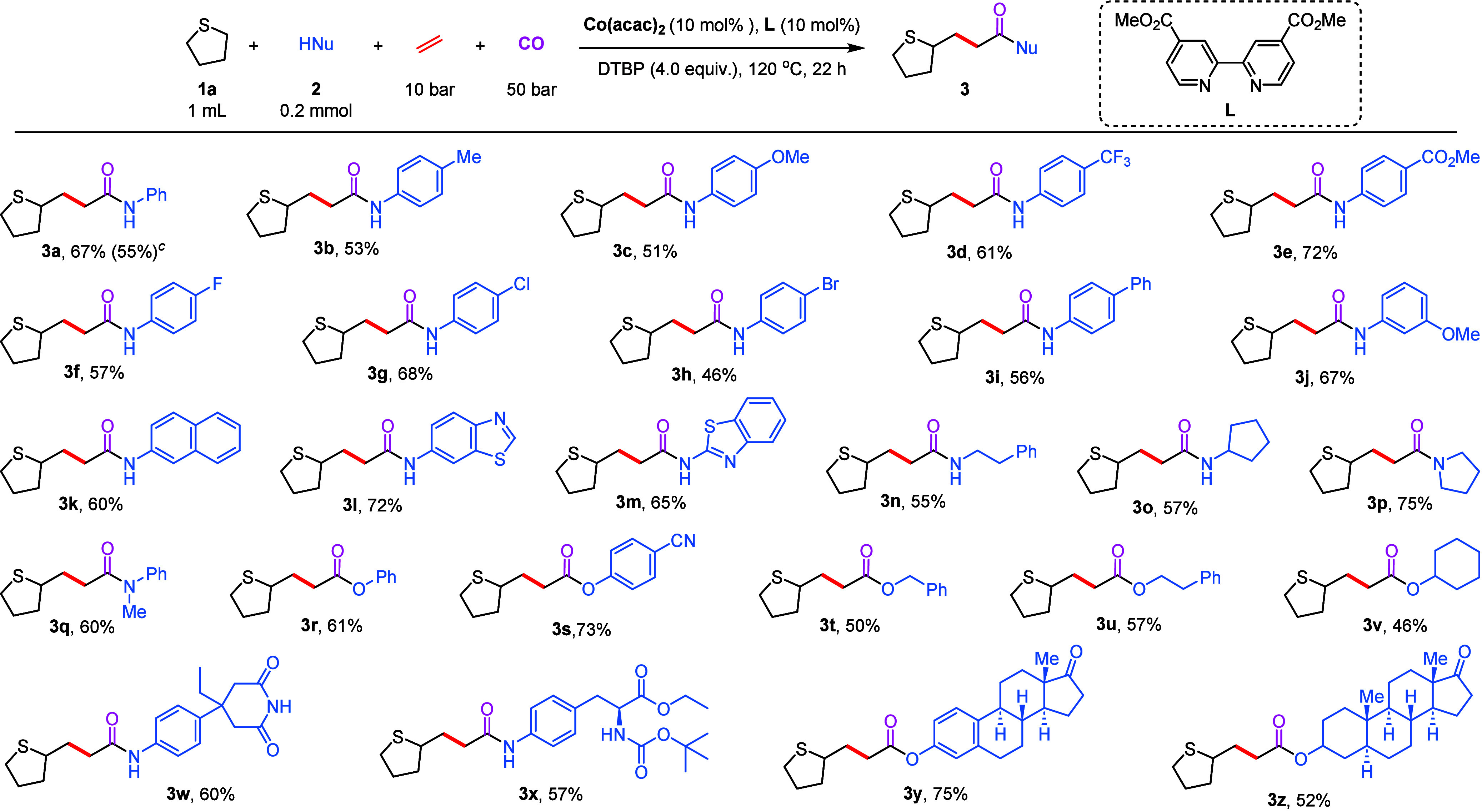
Scope of Nucleophiles^a^,^b^

a Reaction conditions: **1a** (1.0 mL), **2** (0.2 mmol, 1.0 equiv), Co­(acac)_2_ (10 mol %), **L** (10 mol %), DTBP (0.8 mmol), CO (50 bar), ethylene gas (10 bar), 120 °C, 20 h.
b Isolated yields.
c On a 2.0 mmol scale: **1a** (10.0 mL), **2a** (2.0 mmol, 1.0 equiv), Co­(acac)_2_ (10 mol %), **L** (10 mol %), DTBP (4.0 equiv), CO (60 bar), ethylene gas (10 bar), 120 °C, 22 h.

Subsequently, a series of alkylamines were tested, delivering the corresponding carbonylation products in moderate yields (**3n** and **3o**). Notably, secondary amines, such as tetrahydropyrrole **3p** and *N*-methylaniline **3q**, smoothly participated in the four-component carbonylation, giving the desired products in 75% and 60% yields, respectively. Furthermore, phenols and alcohols also served as viable nucleophiles, delivering the target products in moderate to good yields (**3r**–**3v**). To further assess the synthetic applicability of this methodology, several naturally occurring and drug-related molecules (e.g., **3w**–**3z**) were well tolerated and provided the desired products smoothly. To further probe the synthetic utility of this reaction, a gram-scale reaction (2 mmol) was performed under the standard conditions, providing target product **3a** in 55% yield.

Next, the substrate scope of the thioethers was investigated in the multicomponent carbonylation reaction of aniline **2a** (Scheme 3). Various thiol ethers were tested as both solvents and reactants in the subsequent reactions. Initially, various alkyl-substituted thiol ethers provided target products **5a**–**5d** in moderate to good yields. Subsequently, we examined aryl-substituted methyl sulfides; methyl phenyl sulfide gave the desired product **5e** in 52% yield, whereas the use of methyl *p*-tolyl sulfide resulted in a mixture of isomers, with activation at two different methyl positions, giving a 1:1 mixture of isomers **5f**. Furthermore, several common alkenes were tested as the olefin source. Regrettably, both activated and unactivated alkenes in this system predominantly gave direct α-thiolate carbonylation products instead of alkene difunctionalization products (see Table S4 for more details).^11^

**Scheme 3:**
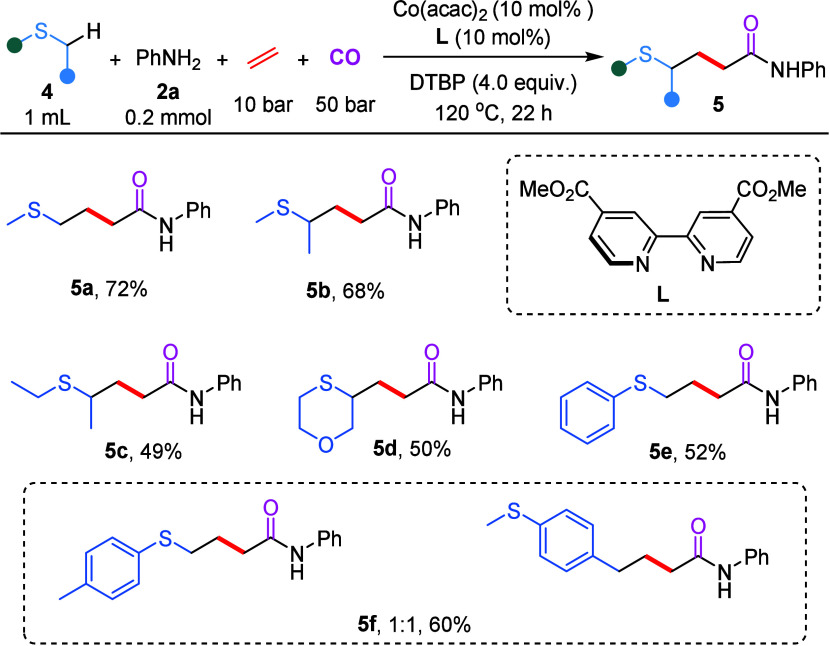
Scope of Thioethers^a^,^b^

a Reaction conditions: **1a** (1.0 mL), **2** (0.2 mmol, 1.0 equiv), Co­(acac)_2_ (10 mol %), **L** (10 mol %), DTBP (0.8 mmol), CO (50 bar), ethylene gas (10 bar), 120 °C, 20 h.
b Isolated yields.

Subsequently, a series of mechanistic studies were conducted to probe the possible pathway of this cobalt-catalyzed four-component carbonylation reaction (Scheme 4). First, the addition of radical scavengers 2,2,6,6-tetramethylpiperidine-1-oxy (TEMPO) completely suppressed the formation of the desired product **3a**. Butylated hydroxytoluene (BHT) significantly inhibited product formation, and an adduct between BHT and a tetrahydrothiophene-derived radical was detected by high-resolution mass spectrometry (HRMS). Next, with the addition of 1.0 equiv of diphenylethylene (DPE), only radical-trapping product **6** was obtained and isolated in 64% yield. These results implied that an alkyl radical is probably involved in this process. Furthermore, a radical clock experiment using (2-cyclopropylallyl)­benzene **7** afforded a ring-opened carbonylated coupling product in 53% yield as a *Z*/*E* mixture, providing additional evidence for the involvement of a sulfur α-carbon radical species in the reaction mechanism (Scheme 4b).

**Scheme 4:**
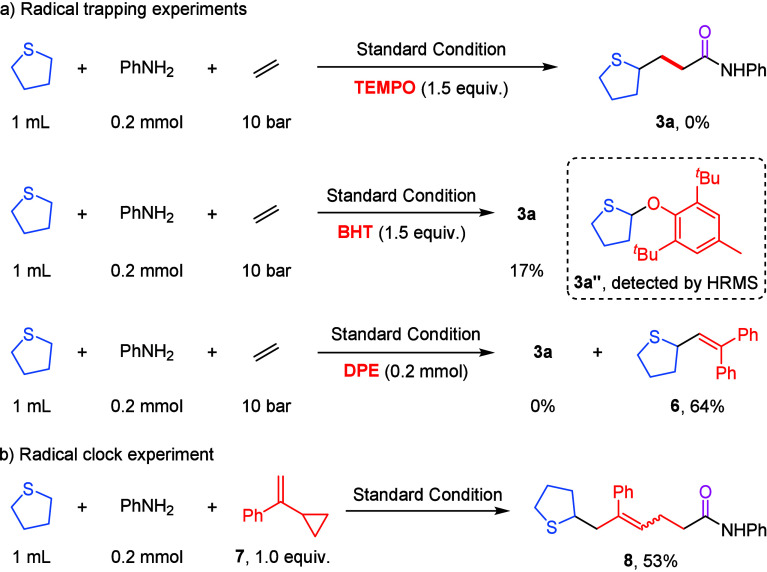
Mechanistic Studies

Based on mechanistic studies and the literature,^12^ a plausible mechanistic pathway is proposed (Scheme 5). First, Co­(I) species can be generated from Co­(II) under the reaction system, in which Co­(II) will be oxidized by DTBP via a single-electron transfer (SET) process to generate a Co­(III) intermediate and then reductive elimination to provide a Co­(I) complex. Meanwhile, the generated *tert*-butoxy radical abstracts the α-hydrogen of the thioether to afford alkyl radical intermediate **A**. Then, radical intermediate **A** is trapped by ethylene to give alkyl radical intermediate **B**, which undergoes radical addition with the Co­(II) species to form alkyl-Co­(III) intermediate **C**. Here, the Co­(II) species can be directly from our precursor or oxidized from the Co­(I) intermediate. Then, intermediate **C** undergoes CO insertion to afford acyl-Co­(III) species **D**. Subsequent anion exchange and reductive elimination yield the final product and regenerate the Co­(I) catalyst, thus completing the catalytic cycle. In alternative path B, radical species **B** may directly trap CO to form acyl radical intermediate **E**, which is subsequently captured by the Co­(II) species. Anion exchange followed by reductive elimination delivers the desired product and regenerates Co­(I) to complete the catalytic cycle.

**Scheme 5:**
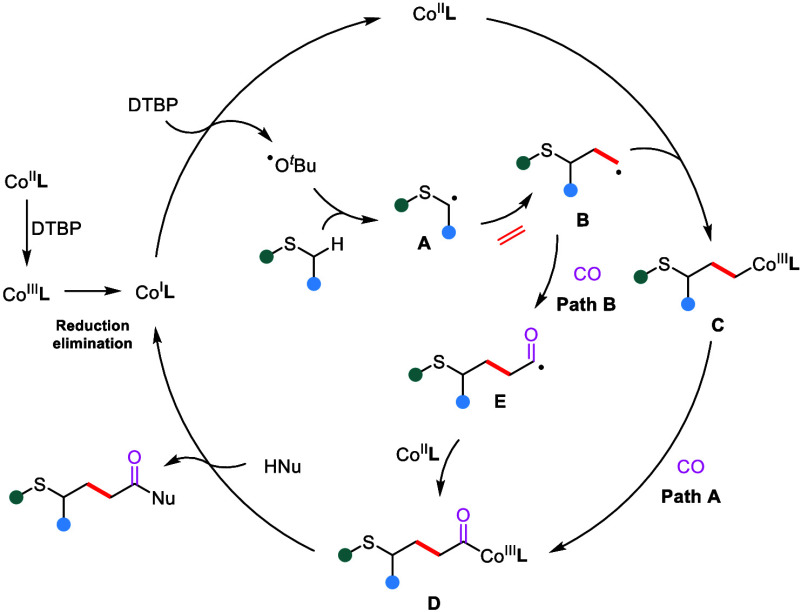
Proposed Mechanism

In summary, we have developed a cobalt-catalyzed four-component functionalization reaction for the synthesis of a series of γ-sulfur-containing carbonylated products with good regioselectivity and excellent functional-group tolerance. Through systematic optimization, the challenge of multicomponent carbonylative coupling can be effectively addressed, delivering a thioether-enabled radical relay carbonylation. Mechanistic control experiments support the generation of an α-carbon radical from the thioether. Overall, this protocol provides an economical and efficient approach to pharmaceutically relevant γ-carbonyl thioethers and expands the toolbox for thioether functionalization and derivatization.

## Supplementary Material



## Data Availability

The data underlying this study are available in the published article and its Supporting Information.

## Notes

General comments, general procedure, optimization details, analytical data, and NMR spectra (PDF)
