# Supplementary material for: Cobalt-Catalyzed Four-Component Carbonylative Synthesis of γ‑Thioesters and γ‑Thioamides from Thioethers and Ethylene
Source: Org Lett. 2025 Nov 7;27(46):12910–5. doi: 10.1021/acs.orglett.5c04357 (PMC12645581; doi:10.1021/acs.orglett.5c04357)

Supporting Information

Cobalt-Catalyzed Four-Component Carbonylative Synthesis of *γ*-Thioesters and *γ*-Thioamides from Thioethers and Ethylene

Xudong Mao,[a,b] Le-Cheng Wang,[a,b] and Xiao-Feng Wu[a,b]*

[a] Dalian National Laboratory for Clean Energy, Dalian Institute of Chemical Physics, Chinese Academy of Sciences, Dalian 116023 China, Email: xwu2020@ dicp.ac.cn

[b] Leibniz-Institut für Katalyse e. V., Albert-Einstein-Straβe 29a, 18059 Rostock, Germany

Contents

1. General Information 1

2. Optimization of Reaction Conditions 2

3. Experimental Procedures and Product Characterization 4

4. Mechanistic Studies 5

5. Spectroscopic Data of Products 7

6. Reference 21

7. The NMR Spectrum 22

1. General Information

General. Unless otherwise noted, all reactions were carried out under carbon monoxide or nitrogen atmosphere. All reagents were from commercial sources and used as received without further purification. All solvents were dried by standard techniques and distilled prior to use. Column chromatography was performed on silica gel (200-300 meshes) using petroleum ether (bp. 60~90 °C), dichloromethane and ethyl acetate as eluent. All NMR spectra were recorded at ambient temperature using Bruker Avance III 400 MHz NMR (^{1}H, 400 MHz; ^{13}C {^{1}H}, 101 MHz, ^{19}F 376 MHz), Bruker AVANCE III HD 700 MHz NMR spectrometers (^{1}H, 700 MHz; ^{13}C{^{1}H}, 175 MHz). ^{1}H NMR chemical shifts are reported relative to TMS and were referenced via residual proton resonances of the corresponding deuterated solvent ($CDCl_3$: 7.26 ppm; d_6-DMSO: 2.50 ppm) whereas ^{13}C{^{1}H} NMR spectra are reported relative to TMS via the carbon signals of the deuterated solvent ($CDCl_3$: 77.0 ppm; d_6-DMSO: 39.5 ppm). Data for ^{1}H are reported as follows: chemical shift (δ ppm), multiplicity (s = singlet, d = doublet, t = triplet, q = quartet, dd (doublet of doublets), dt (doublet of triplets), quint = quintet, m = multiplet, br = broad), coupling constant (Hz), and integration. All ^{13}C NMR spectra were broad band ^{1}H decoupled. All reactions were monitored by GC-FID or NMR analysis. HRMS data was obtained with Micromass HPLC-Q-TOF mass spectrometer (ESI-TOF) or Agilent 6540 Accurate-MS spectrometer (Q-TOF).

Caution!!! (The high toxicity of carbon monoxide, all the reactions should be performed in an autoclave. The laboratory should be well-equipped with a CO detector and alarm system.)

2. Optimization of Reaction Conditions

Table S1. Optimization of metals

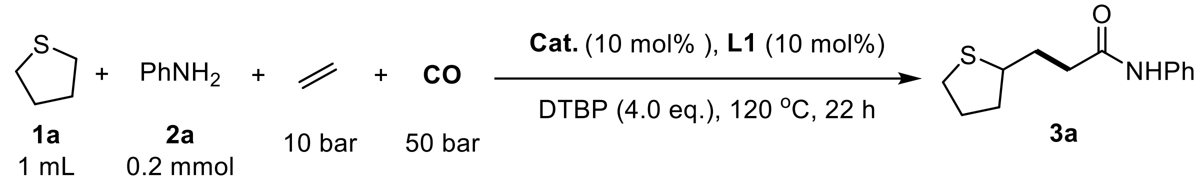

Entry	**Catalysts**	Yield **3a** (%)[a]
1	$Co(acac)_2$	70(67)[b]
2	$Co(acac)_3$	62
3	$Co(OAc)_2$	31
4	$CoCl_2$	0
5	$CoBr_2$	0
6	$Fe(acac)_3$	0
7	$Ni(acac)_2$	0
8	$Cu(acac)_2$	0
9	$PdCl_2$	21

[a]Yields were determined by GC analysis using hexadecane as an internal standard. [b]Isolated yield.

Table S2. Optimization of ligands

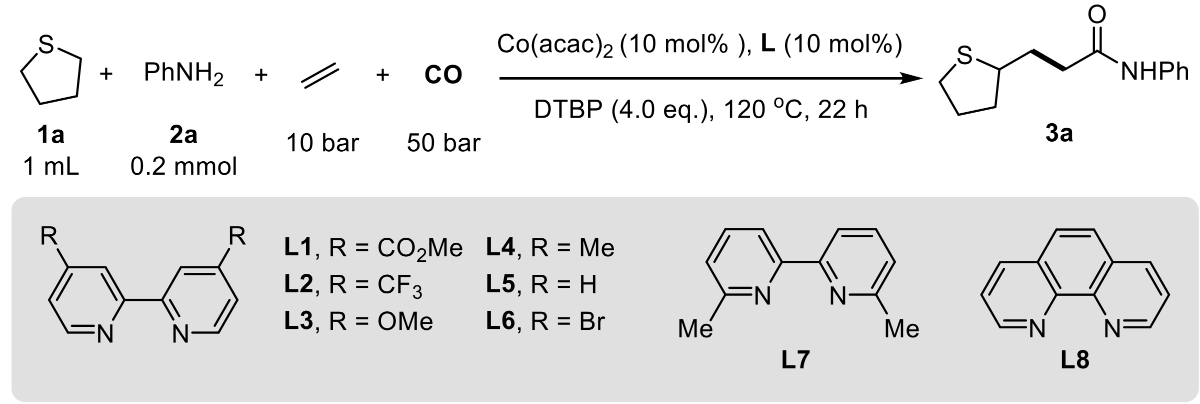

Entry	**Ligands**	Yield **3a** (%)[a]
1	**L2**	52
2	**L3**	56
3	**L4**	32
4	**L5**	50
5	**L6**	35
6	**L7**	34
7	**L8**	61

[a]Yields were determined by GC analysis using hexadecane as an internal standard.

Table S3. Optimization of peroxides

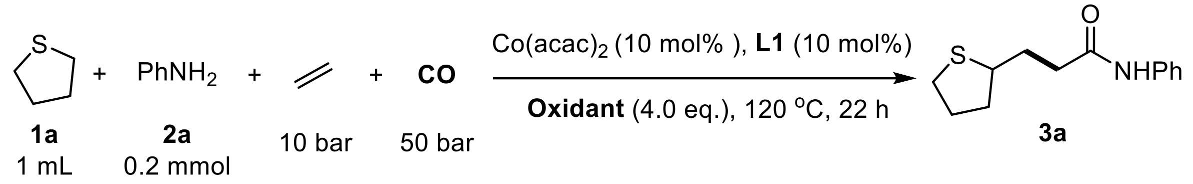

Entry	**Oxidants**	Yield **3a** (%)[a]
1	BPO	0
2	TBHP	0
3	H_2O_2	0
4	$K_2S_2O_8$	0
5	*m*-CPBA	0
6	TBPB	0

[a]Yields were determined by GC analysis using hexadecane as an internal standard.

Table S4. Unsuccessful and Challenging Substrates[a]

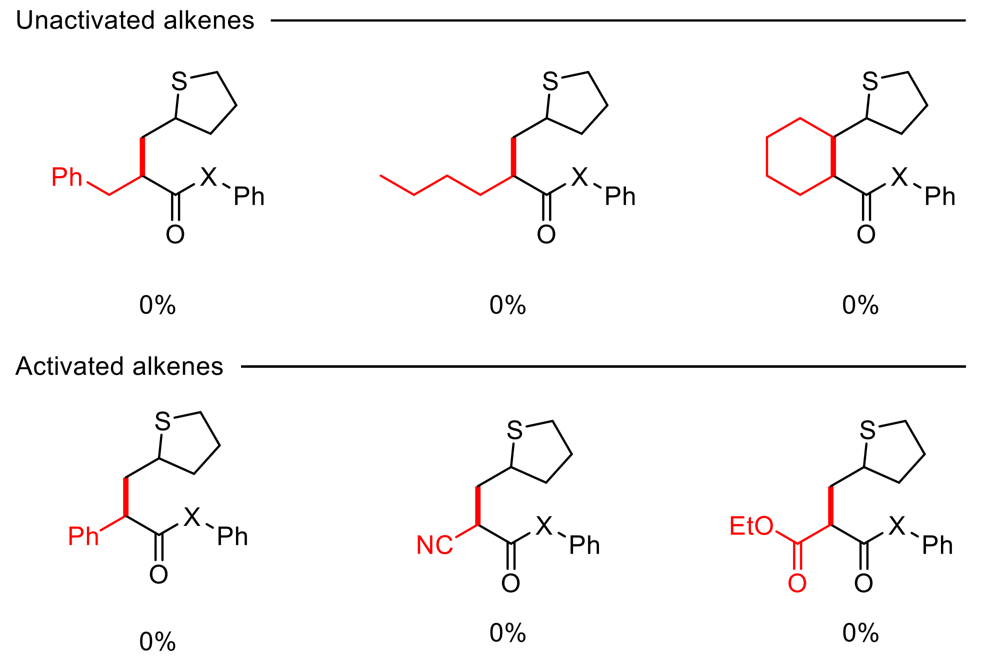

[a]Reaction conditions (X = NH or O): thioether (1.0 mL), nucleophiles (0.2 mmol, 1.0 equiv.), $Co(acac)_2$ (10 mol%), Ligand (10 mol%), DTBP (4.0 equiv.), CO (60 bar), 120 °C, 22 h.

3. Experimental Procedures and Product Characterization

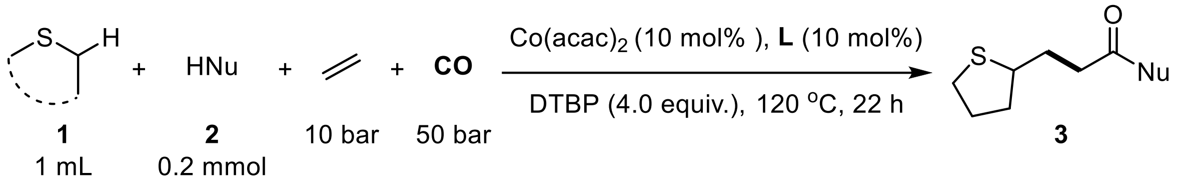

General Procedure: Under an argon atmosphere, a 4 mL screw-cap vial was charged with equipped with a magnetic stir bar was charged with Co(acac)$_2$ (5.1 mg, 0.02 mmol, 10 mol%), **L** (5.5 mg, 0.02 mmol, 10 mol%), **1** sulfides (1 mL, 0.2 M), **2** amines (1 equiv., 0.2 mmol) and DTBP (4.0 equiv.) were added nitrogen atmosphere, the vial was moved to an alloy plate and put into a Parr 4560 series autoclave (300 mL) under an argon atmosphere. At room temperature, the autoclave was flushed with CO three times and charged with 50 bar of CO and 10 bar of ethylene gas. The autoclave was placed on a heating plate equipped with a magnetic stirrer and an aluminum block. The reaction mixture was heated to 120 °C for 22 h. After the reaction was complete, the autoclave was cooled down with ice water to room temperature and the pressure was released carefully. After cooling to room temperature, the reaction mixture was directly purified by column chromatography on silica gel using petroleum ether and ethyl acetate to afford the corresponding product.

Procedure for 2 mmol-scale reaction: Under an argon atmosphere, a 25 mL flask was charged with equipped with a magnetic stir bar was charged with Co(acac)$_2$ (51.4 mg, 0.2 mmol, 10 mol%), **L** (54.4 mg, 0.2 mmol, 10 mol%), **1a** sulfides (10 mL, 0.2 M), **2a** amines (1 equiv., 2 mmol) and DTBP (4.0 equiv.) were added nitrogen atmosphere, the vial was moved to an alloy plate and put into a Parr 4560 series autoclave (300 mL) under an argon atmosphere. At room temperature, the autoclave was flushed with CO three times and charged with 50 bar of CO and 10 bar of ethylene gas. The autoclave was placed on a heating plate equipped with a magnetic stirrer and an aluminum block. The reaction mixture was heated to 120 °C for 22 h. After the reaction was complete, the autoclave was cooled down with ice water to room temperature and the pressure was released carefully. After cooling to room temperature, the reaction mixture was directly purified by column chromatography on silica gel using petroleum ether and ethyl acetate to afford the corresponding product **3a** in 55% yield (258.5 mg).

4. Mechanistic Studies

Radical trapping experiment

Following the standard procedure of the model reaction, when 1.5 equiv. of radical inhibitor 2,2,6,6-tetramethylpiperidine-1-oxy (TEMPO) was added to the reaction mixture, the formation of the desired product **3a** was completely inhibited. When 2,6-di-tert-butyl-4-methylphenol (BHT) was added to the reaction mixture, the formation of the desired product **3a** was partly inhibited. The adducts of phosphinoyl radical by BHT were confirmed by high-resolution mass spectrometry (HRMS).

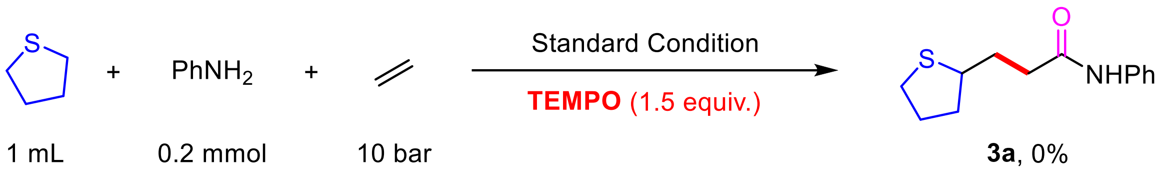

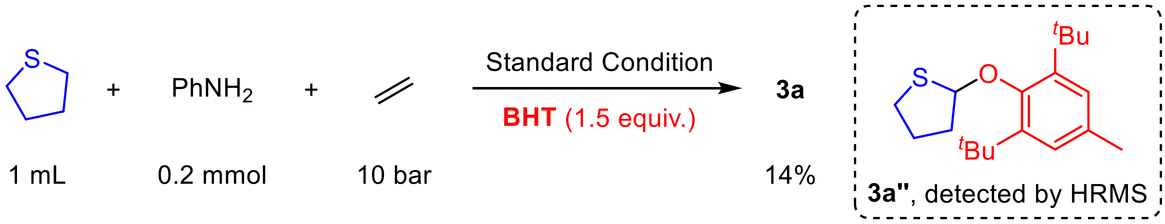

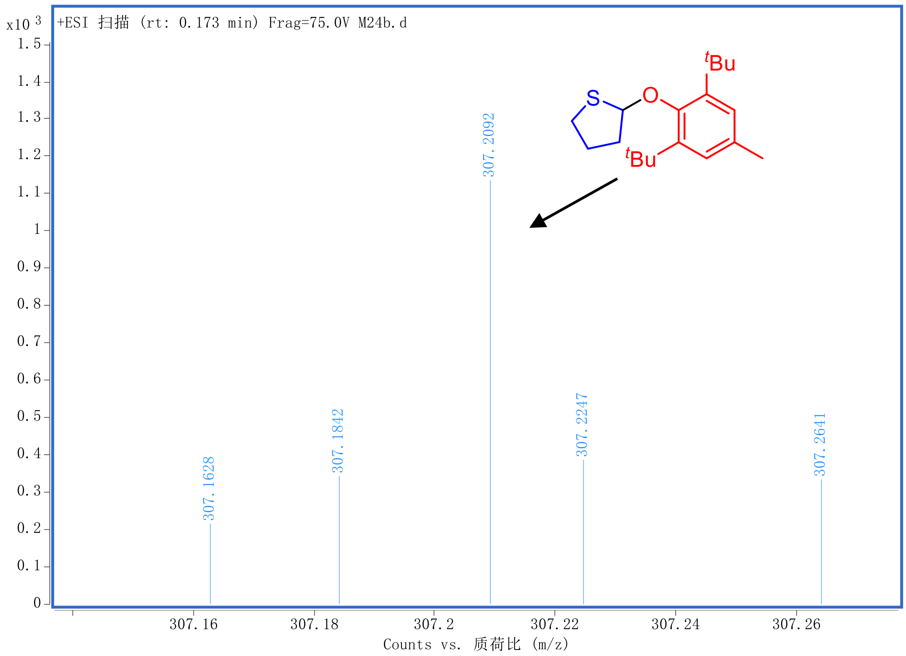

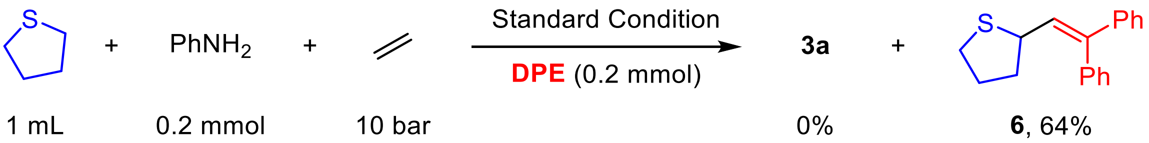

Following the standard procedure of the model reaction, when 0.2 mmol of radical inhibitor 1,1-diphenylethylene (DPE) was added to the reaction mixture, the formation of the desired

product **3a** was completely inhibited, and the coupling product **6** was obtained in 64% yield.

2-(2,2-Diphenylvinyl)tetrahydrothiophene (**6**)

34.0 mg, 64% yield, a colorless oil, eluent: petroleum ether/ethyl acetate = 20:1.

^{1}H NMR (700 MHz, CDCl$_3$) δ 7.38 (t, *J* = 7.5 Hz, 2H), 7.32 (t, *J* = 7.4 Hz, 1H), 7.24 (d, *J* = 7.2 Hz, 2H), 7.23-7.17 (m, 5H), 6.04 (d, *J* = 10.2 Hz, 1H), 4.08-3.89 (m, 1H), 3.09-2.95 (m, 1H), 2.90-2.78 (m, 1H), 2.26-2.06 (m, 2H), 1.92-1.80 (m, 1H), 1.80-1.70 (m, 1H).

^{13}C NMR (175 MHz, CDCl$_3$) δ 142.0, 141.6, 139.4, 130.6, 129.9, 128.2, 128.0, 127.4, 127.21, 127.20, 47.7, 38.6, 33.3, 31.2.

Radical clock experiments

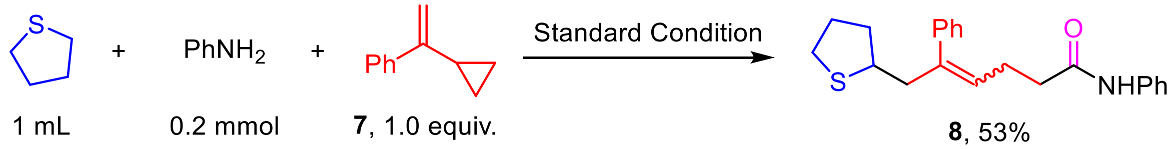

The radical clock reaction was performed according to the general procedure. Following the standard procedure of the model reaction, when 1.0 equiv. (1-cyclopropylvinyl)benzene was added to the reaction mixture. The ring-opened product **8** was obtained as an E/Z mixture of alkenes in 53% yield.

***N*,5-Diphenyl-6-(tetrahydrothiophen-2-yl)hex-4-enamide** (**8**)

37.2 mg, 53% yield, a colorless oil, eluent: petroleum ether/ethyl acetate = 100:1.

^{1}H NMR (700 MHz, CDCl$_3$) δ 7.57 (s, 0.6H), 7.53 (d, *J* = 8.0 Hz, 1.3H), 7.48 (d, *J* = 8.0 Hz, 0.8H), 7.34-7.28 (m, 4.5H), 7.27-7.21 (m, 1.7H), 7.13 (d, *J* = 7.0 Hz, 0.8H), 7.11-7.05 (m, 1.2H), 5.67 (t, *J* = 7.4 Hz, 0.6H), 5.61-5.53 (m, 0.4H), 3.36-3.29 (m, 0.6H), 3.28-3.21 (m, 0.4H), 2.90-2.81 (m, 2.2H), 2.81-2.75 (m, 1.2H), 2.72-2.62 (m, 1.8H), 2.57-2.48 (m, 1.8H), 2.43-2.29 (m, 1.7H), 2.06-2.00 (m, 1H), 1.98-1.93 (m, 1H), 1.83-1.76 (m, 1H), 1.59-1.50 (m, 1H).

^{13}C NMR (175 MHz, CDCl$_3$) δ 170.7, 170.6, 142.2, 141.5, 140.5, 139.8, 137.91, 137.86, 129.0, 128.93, 128.87, 128.3, 128.2, 127.0, 126.9, 126.7, 126.6, 124.2, 124.1, 119.80, 119.78, 47.6, 47.24, 47.17, 38.0, 37.6, 37.2, 36.9, 36.7, 32.3, 32.2, 30.2, 24.9, 24.8.

5. Spectroscopic Data of Products

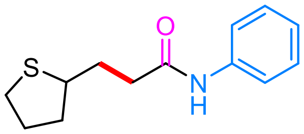

3a

***N*-Phenyl-3-(tetrahydrothiophen-2-yl)propanamide (3a)** Prepared according to the general procedure from **1a** (1 mL, 0.2 M) and **2a** (0.20 mmol) purified by column chromatography on silica gel with petroleum ether/ethyl acetate (10:1) to provide the title compound **3a** as a red solid (31.5 mg, 67 % yield); m.p. 78.3-80.0 °C; ^{1}H NMR (400 MHz, $CDCl_3$) δ 7.51 (d, *J* = 8.0 Hz, 3H), 7.30 (t, *J* = 7.9 Hz, 2H), 7.09 (t, *J* = 7.4 Hz, 1H), 3.46-3.33 (m, 1H), 2.93-2.80 (m, 2H), 2.53-2.44 (m, 1H), 2.44-2.33 (m, 1H), 2.23-2.13 (m, 1H), 2.13-2.02 (m, 2H), 1.98-1.87 (m, 1H), 1.87-1.78 (m, 1H), 1.68-1.56 (m, 1H); ^{13}C NMR (100 MHz, $CDCl_3$) δ 170.7, 137.9, 128.9, 124.2, 119.8, 48.6, 37.3, 36.8, 33.2, 32.3, 30.1; HRMS (ESI) Calcd for $C_{13}H_{18}NOS$ $[M + H]^+$ 236.1104, found 236.1107.

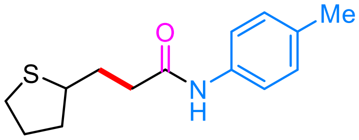

3b

3-(Tetrahydrothiophen-2-yl)-*N*-(*p*-tolyl)propanamide (3b) Prepared according to the general procedure from **1a** (1 mL, 0.2 M) and **2b** (0.20 mmol) purified by column chromatography on silica gel with petroleum ether/ethyl acetate (10:1) to provide the title compound **3b** as a red solid (26.4 mg, 53% yield); m.p. 71.3-72.7 °C; ^{1}H NMR (400 MHz, $CDCl_3$) δ 7.38 (d, *J* = 8.3 Hz, 3H), 7.10 (d, *J* = 8.2 Hz, 2H), 3.45-3.34 (m, 1H), 2.93-2.83 (m, 2H), 2.54-2.42 (m, 1H), 2.41-2.34 (m, 1H), 2.30 (s, 3H), 2.23-2.14 (m, 1H), 2.13-2.02 (m, 2H), 1.98-1.87 (m, 1H), 1.87-1.77 (m, 1H), 1.67-1.56 (m, 1H); ^{13}C NMR (101 MHz, $CDCl_3$) δ 170.6, 135.3, 133.8, 129.4, 119.9, 48.6, 37.3, 36.8, 33.2, 32.2, 30.2, 20.8; HRMS (ESI) Calcd for $C_{14}H_{20}NOS$ $[M + H]^+$ 250.1260, found 250.1264.

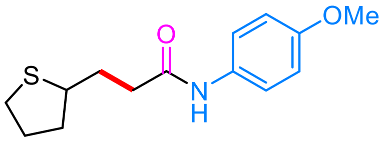

3c

***N*-(4-Methoxyphenyl)-3-(tetrahydrothiophen-2-yl)propanamide (3c)** Prepared according to the general procedure from **1a** (1 mL, 0.2 M) and **2c** (0.20 mmol) purified by column chromatography on silica gel with petroleum ether/ethyl acetate (10:1) to provide the title compound **3c** as a dark yellow solid (27.1 mg, 51% yield); m.p. 75.8-77.1; ^{1}H NMR (400 MHz, $CDCl_3$) δ 7.45-7.36 (m, 2H), 7.27 (s, 1H), 6.87-6.82 (m, 2H), 3.78 (s, 3H), 3.46-3.35 (m, 1H), 2.94-2.83 (m, 2H), 2.52-2.42 (m, 1H), 2.42-2.32 (m, 1H), 2.24-2.15 (m, 1H), 2.15-2.04 (m, 2H), 1.98-1.87 (m, 1H), 1.86-1.76 (m, 1H), 1.67-1.58 (m, 1H); ^{13}C NMR (101 MHz, $CDCl_3$) δ 170.5, 156.3, 130.9, 121.7, 114.1, 55.4, 48.6, 37.4, 36.7, 33.3, 32.3, 30.2; HRMS (ESI) Calcd for $C_{14}H_{20}NO_2S$ $[M + H]^+$ 266.1209, found 266.1214.

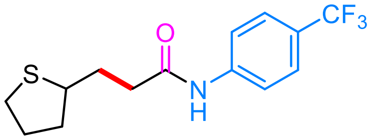

3d

3-(Tetrahydrothiophen-2-yl)-*N*-(4-(trifluoromethyl)phenyl)propanamide (3d) Prepared according to the general procedure from **1a** (1 mL, 0.2 M) and **2d** (0.20 mmol) purified by column chromatography on silica gel with petroleum ether/ethyl acetate (10:1) to provide the title compound **3d** as a red solid (37.0 mg, 61% yield); m.p. 100.0-101.9 °C; ^{1}H NMR (400 MHz, $CDCl_3$) δ 7.64 (d, J = 8.5 Hz, 2H), 7.57 (d, J = 8.5 Hz, 2H), 7.46 (s, 1H), 3.48-3.36 (m, 1H), 2.95-2.83 (m, 2H), 2.58-2.48 (m, 1H), 2.48-2.37 (m, 1H), 2.26-2.16 (m, 1H), 2.16-2.02 (m, 2H), 2.01-1.88 (m, 1H), 1.87-1.76 (m, 1H), 1.68-1.61 (m, 1H); ^{13}C NMR (101 MHz, $CDCl_3$) δ 171.0, 140.9, 126.2 (q, $J_{C\text{-}F}$ = 4 Hz), 126.0 (q, $J_{C\text{-}F}$ = 34 Hz), 124.0 (q, $J_{C\text{-}F}$ = 270 Hz), 119.3, 48.5, 37.4, 36.9, 33.0, 32.3, 30.2; ^{19}F NMR (376 MHz, $CDCl_3$) δ -62.1; HRMS (ESI) Calcd for $C_{14}H_{17}F_3NOS$ $[M + H]^+$ 304.0977, found 304.0982.

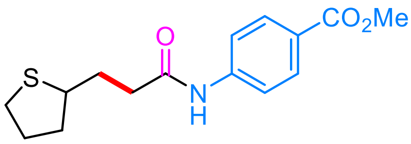

3e

Methyl 4-(3-(tetrahydrothiophen-2-yl)propanamido)benzoate (3e) Prepared according to the general procedure from **1a** (1 mL, 0.2 M) and **2e** (0.20 mmol) purified by column chromatography on silica gel with petroleum ether/ethyl acetate (10:1) to provide the title compound **3e** as a red solid (42.2 mg, 72% yield); m.p. 71.5-73.0 °C; ^{1}H NMR (400 MHz, $CDCl_3$) δ 7.99 (d, *J* = 8.7 Hz, 2H), 7.85 (s, 1H), 7.62 (d, *J* = 8.5 Hz, 2H), 3.90 (s, 3H), 3.44-3.35 (m, 1H), 2.90-2.83 (m, 2H), 2.58-2.49 (m, 1H), 2.47-2.39 (m, 1H), 2.24-2.15 (m, 1H), 2.13-2.03 (m, 2H), 1.98-1.89 (m, 1H), 1.87-1.78 (m, 1H), 1.67-1.58 (m, 1H); ^{13}C NMR (101 MHz, $CDCl_3$) δ 171.1, 166.6, 142.1, 130.7, 125.4, 118.8, 52.0, 48.5, 37.3, 36.8, 33.0, 32.3, 30.1; HRMS (ESI) Calcd for $C_{15}H_{20}NO_3S$ $[M + H]^+$ 294.1158, found 294.1165.

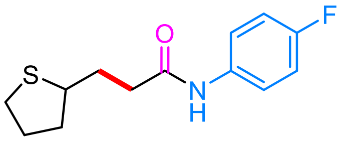

3f

***N*-(4-Fluorophenyl)-3-(tetrahydrothiophen-2-yl)propanamide (3f)** Prepared according to the general procedure from **1a** (1 mL, 0.2 M) and **2f** (0.20 mmol) purified by column chromatography on silica gel with petroleum ether/ethyl acetate (5:1) to provide the title compound **3f** as a red solid (28.9 mg, 57% yield); m.p. 64.3-66.0 °C; ^{1}H NMR (400 MHz, $CDCl_3$) δ 7.50 (s, 1H), 7.48-7.42 (m, 2H), 6.99 (t, *J* = 8.7 Hz, 2H), 3.45-3.34 (m, 1H), 2.94-2.81 (m, 2H), 2.53-2.44 (m, 1H), 2.43-2.33 (m, 1H), 2.23-2.14 (m, 1H), 2.14-2.01 (m, 2H), 1.98-1.87 (m, 1H), 1.85-1.76 (m, 1H), 1.67-1.57 (m, 1H); ^{13}C NMR (101 MHz, $CDCl_3$) δ 170.7, 159.3 (d, J_{C-F} = 242 Hz), 133.83 (d, J_{C-F} = 3 Hz), 121.71 (d, J_{C-F} = 7 Hz), 115.5, (d, J_{C-F} = 22 Hz), 48.6, 37.3, 36.7, 33.1, 32.3, 30.1; ^{19}F NMR (376 MHz, $CDCl_3$) δ -118.1; HRMS (ESI) Calcd for $C_{13}H_{17}FNOS$ $[M + H]^+$ 254.1009, found 254.1010.

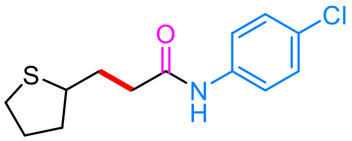

3g

***N*-(4-Chlorophenyl)-3-(tetrahydrothiophen-2-yl)propanamide (3g)** Prepared according to the general procedure from **1a** (1 mL, 0.2 M) and **2g** (0.20 mmol) purified by column chromatography on silica gel with petroleum ether/ethyl acetate (10:1) to provide the title compound **3g** as a white solid (36.7 mg, 68% yield); m.p. 97.3-98.9 °C; ^{1}H NMR (400 MHz, $CDCl_3$) δ 7.44-7.39 (m, 4H), 7.35 (s, 1H), 3.45-3.32 (m, 1H), 2.95-2.80 (m, 2H), 2.54-2.32 (m, 2H), 2.24-2.15 (m, 1H), 2.15-2.02 (m, 2H), 2.00-1.87 (m, 1H), 1.86-1.74 (m, 1H), 1.67-1.58 (m, 1H); ^{13}C NMR (101 MHz, $CDCl_3$) δ 170.8, 136.9, 131.9, 121.4, 116.8, 48.5, 37.3, 36.8, 33.0, 32.3, 30.1; HRMS (ESI) Calcd for $C_{13}H_{17}ClNOS$ $[M + H]^+$ 270.0714, found 270.0711.

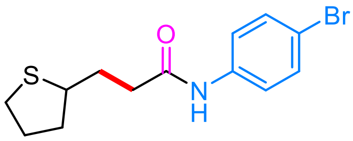

3h

***N*-(4-Bromophenyl)-3-(tetrahydrothiophen-2-yl)propanamide (3h)** Prepared according to the general procedure from **1a** (1 mL, 0.2 M) and **2h** (0.20 mmol) purified by column chromatography on silica gel with petroleum ether/ethyl acetate (10:1) to provide the title compound **3h** as a white solid (31.5 mg, 46% yield); m.p. 81.3-83.2 °C; ^{1}H NMR (400 MHz, $CDCl_3$) δ 7.47 (d, J = 7.8 Hz, 2H), 7.39 (s, 1H), 7.32-7.20 (m, 2H), 3.49-3.32 (m, 1H), 2.98-2.79 (m, 2H), 2.54-2.35 (m, 2H), 2.25-2.15 (m, 1H), 2.15-2.00 (m, 2H), 2.00-1.88 (m, 1H), 1.86-1.75 (m, 1H), 1.68-1.56 (m, 1H); ^{13}C NMR (101 MHz, $CDCl_3$) δ 170.7, 136.4, 129.2, 129.0, 121.0, 48.6, 37.4, 36.8, 33.1, 32.3, 30.2; HRMS (ESI) Calcd for $C_{13}H_{17}BrNOS$ $[M + H]^+$ 314.0209, found 314.0201

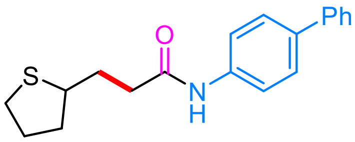

3i

***N*-([1,1'-Biphenyl]-4-yl)-3-(tetrahydrothiophen-2-yl)propanamide (3i)** Prepared according to the general procedure from **1a** (1 mL, 0.2 M) and **2i** (0.20 mmol) purified by column

chromatography on silica gel with petroleum ether/ethyl acetate (10:1) to provide the title compound **3i** as a red solid (34.9 mg, 56% yield); m.p. 111.3-112.7 °C; ^{1}H NMR (400 MHz, $CDCl_3$) δ 7.61-7.52 (m, 6H), 7.46 (s, 1H), 7.41 (t, J = 7.6 Hz, 2H), 7.35-7.29 (m, 1H), 3.47-3.37 (m, 1H), 2.94-2.83 (m, 2H), 2.57-2.47 (m, 1H), 2.46-2.37 (m, 1H), 2.27-2.16 (m, 1H), 2.15-2.02 (m, 2H), 1.98-1.79 (m, 2H), 1.68-1.58 (m, 1H); ^{13}C NMR (101 MHz, $CDCl_3$) δ 170.7, 140.4, 137.13, 137.05, 128.7, 127.6, 127.0, 126.8, 120.1, 48.6, 37.4, 36.9, 33.2, 32.3, 30.2; HRMS (ESI) Calcd for $C_{19}H_{22}NOS$ $[M + H]^+$ 312.1417, found 312.1420.

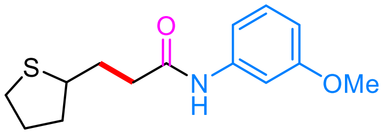

3j

***N*-(3-Methoxyphenyl)-3-(tetrahydrothiophen-2-yl)propanamide (3j)** Prepared according to the general procedure from **1a** (1 mL, 0.2 M) and **2j** (0.20 mmol) purified by column chromatography on silica gel with petroleum ether/ethyl acetate (10:1) to provide the title compound **3j** as a dark yellow oil (35.6 mg, 67% yield); m.p. 65.7-67.9 °C; ^{1}H NMR (400 MHz, $CDCl_3$) δ 7.42 (s, 1H), 7.31 (s, 1H), 7.19 (t, J = 8.1 Hz, 1H), 6.97 (d, J = 6.6 Hz, 1H), 6.65 (dd, J = 8.4, 2.5 Hz, 1H), 3.79 (s, 3H), 3.47-3.34 (m, 1H), 2.95-2.81 (m, 2H), 2.53-2.43 (m, 1H), 2.43-2.33 (m, 1H), 2.24-2.13 (m, 1H), 2.13-2.01 (m, 2H), 1.99-1.87 (m, 1H), 1.87-1.76 (m, 1H), 1.68-1.55 (m, 1H); ^{13}C NMR (101 MHz, $CDCl_3$) δ 170.7, 160.1, 139.1, 129.6, 111.8, 110.1, 105.4, 55.2, 48.5, 37.3, 36.9, 33.1, 32.3, 30.1; HRMS (ESI) Calcd for $C_{14}H_{20}NO_2S$ $[M + H]^+$ 266.1209, found 266.1210.

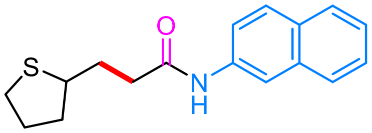

3k

***N*-(Naphthalen-2-yl)-3-(tetrahydrothiophen-2-yl)propanamide (3k)** Prepared according to the general procedure from **1a** (1 mL, 0.2 M) and **2k** (0.20 mmol) purified by column chromatography on silica gel with petroleum ether/ethyl acetate (10:1) to provide the title compound **3k** as a dark yellow oil (34.2 mg, 60% yield); ^{1}H NMR (400 MHz, $CDCl_3$) δ 8.20 (s, 1H), 7.76 (d, J = 8.3 Hz, 3H), 7.51 (s, 1H), 7.47-7.36 (m, 3H), 3.50-3.34 (m, 1H), 2.96-2.81 (m, 2H), 2.62-2.50 (m, 1H), 2.50-2.39 (m, 1H), 2.30-2.17 (m, 1H), 2.16-2.02 (m, 2H), 1.99-1.80 (m, 2H), 1.67-1.58 (m, 1H); ^{13}C NMR (101 MHz, $CDCl_3$) δ 170.9, 135.3, 133.8,

130.6, 128.7, 127.6, 127.5, 126.5, 124.9, 119.8, 116.6, 48.6, 37.4, 36.9, 33.2, 32.3, 30.2; HRMS (ESI) Calcd for $C_{17}H_{20}NOS$ [M + H]$^+$ 286.1260, found 286.1253.

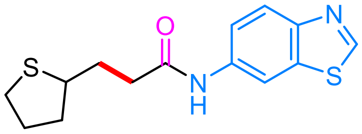

3l

***N*-(Benzo[*d*]thiazol-6-yl)-3-(tetrahydrothiophen-2-yl)propanamide (3l)** Prepared according to the general procedure from **1a** (1 mL, 0.2 M) and **2l** (0.20 mmol) purified by column chromatography on silica gel with petroleum ether/ethyl acetate (10:1) to provide the title compound **3l** as a colorless oil (42.1 mg, 72% yield); ^{1}H NMR (400 MHz, $CDCl_3$) δ 8.91 (s, 1H), 8.56 (d, *J* = 2.1 Hz, 1H), 8.03 (d, *J* = 8.8 Hz, 1H), 7.61 (s, 1H), 7.33 (dd, *J* = 8.8, 2.2 Hz, 1H), 3.50-3.36 (m, 1H), 2.95-2.83 (m, 2H), 2.60-2.51 (m, 1H), 2.51-2.41 (m, 1H), 2.28-2.19 (m, 1H), 2.17-2.05 (m, 2H), 1.99-1.91 (m, 1H), 1.89-1.80 (m, 1H), 1.68-1.61 (m, 1H); ^{13}C NMR (101 MHz, $CDCl_3$) δ 170.9, 153.4, 149.9, 135.6, 134.8, 123.5, 118.9, 112.5, 48.6, 37.4, 37.0, 33.2, 32.3, 30.2; HRMS (ESI) Calcd for $C_{14}H_{17}N_2OS_2$ [M + H]$^+$ 293.0777, found 293.0779.

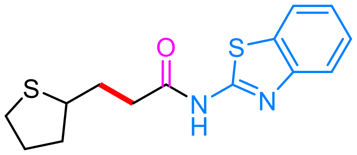

3m

***N*-(Benzo[*d*]thiazol-2-yl)-3-(tetrahydrothiophen-2-yl)propanamide (3m)** Prepared according to the general procedure from **1a** (1 mL, 0.2 M) and **2m** (0.20 mmol) purified by column chromatography on silica gel with petroleum ether/ethyl acetate (10:1) to provide the title compound **3m** as a white solid (38.0 mg, 65% yield); m.p. 117.6-118.9 °C; ^{1}H NMR (400 MHz, $CDCl_3$) δ 11.46 (s, 1H), 7.83 (dd, *J* = 17.3, 8.0 Hz, 2H), 7.55-7.42 (m, 1H), 7.40-7.29 (m, 1H), 3.36-3.23 (m, 1H), 2.87-2.75 (m, 2H), 2.70-2.46 (m, 2H), 2.16-2.06 (m, 1H), 2.05-1.92 (m, 2H), 1.92-1.80 (m, 2H), 1.57-1.46 (m, 1H); ^{13}C NMR (101 MHz, $CDCl_3$) δ 171.3, 159.6, 147.7, 131.9, 126.4, 124.0, 121.6, 120.5, 48.0, 37.1, 35.4, 32.29, 32.28, 30.1; HRMS (ESI) Calcd for $C_{14}H_{17}N_2OS_2$ [M + H]$^+$ 293.0777, found 293.0771.

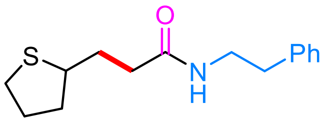

3n

***N*-Phenethyl-3-(tetrahydrothiophen-2-yl)propanamide (3n)** Prepared according to the general procedure from **1a** (1 mL, 0.2 M) and **2n** (0.20 mmol) purified by column chromatography on silica gel with petroleum ether/ethyl acetate (10:1) to provide the title compound **3n** as a dark yellow oil (29.0 mg, 55% yield); ^{1}H NMR (400 MHz, $CDCl_3$) δ 7.31 (t, J = 7.3 Hz, 2H), 7.26-7.22 (m, 1H), 7.22-7.17 (m, 2H), 5.51 (s, 1H), 3.52 (q, J = 6.7, 6.3 Hz, 2H), 3.36-3.26 (m, 1H), 2.89-2.78 (m, 4H), 2.31-2.21 (m, 1H), 2.20-2.12 (m, 1H), 2.12-2.02 (m, 3H), 1.95-1.85 (m, 1H), 1.75-1.65 (m, 1H), 1.63-1.55 (m, 1H); ^{13}C NMR (101 MHz, $CDCl_3$) δ 172.3, 138.8, 128.7, 128.6, 126.5, 48.6, 40.5, 37.3, 35.9, 35.7, 33.3, 32.2, 30.1; HRMS (ESI) Calcd for $C_{15}H_{22}NOS$ $[M + H]^+$ 264.1417, found 264.1413.

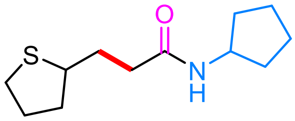

3o

***N*-Cyclopentyl-3-(tetrahydrothiophen-2-yl)propanamide (3o)** Prepared according to the general procedure from **1a** (1 mL, 0.2 M) and **2o** (0.20 mmol) purified by column chromatography on silica gel with petroleum ether/ethyl acetate (10:1) to provide the title compound **3o** as a dark yellow solid (25.9 mg, 57% yield); m.p. 117.6-118.9 °C; ^{1}H NMR (400 MHz, $CDCl_3$) δ 5.49 (s, 1H), 4.28-4.13 (m, 1H), 3.45-3.25 (m, 1H), 2.96-2.77 (m, 2H), 2.32-2.22 (m, 1H), 2.22-2.14 (m, 1H), 2.14-2.04 (m, 3H), 2.03-1.88 (m, 3H), 1.75-1.55 (m, 6H), 1.41-1.31 (m, 2H); ^{13}C NMR (101 MHz, $CDCl_3$) δ 171.8, 51.1, 48.7, 37.3, 36.0, 33.5, 33.12, 33.07, 32.2, 30.2, 23.7; HRMS (ESI) Calcd for $C_{12}H_{22}NOS$ $[M + H]^+$ 228.1417, found 228.1414.

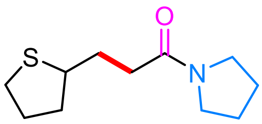

3p

1-(Pyrrolidin-1-yl)-3-(tetrahydrothiophen-2-yl)propan-1-one (3p) Prepared according to the general procedure from **1a** (1 mL, 0.2 M) and **2p** (0.20 mmol) purified by column

chromatography on silica gel with petroleum ether/ethyl acetate (10:1) to provide the title compound **3p** as a dark yellow oil (32.0 mg, 75% yield); ^{1}H NMR (400 MHz, $CDCl_3$) δ 3.52-3.33 (m, 5H), 2.93-2.80 (m, 2H), 2.44-2.33 (m, 1H), 2.33-2.23 (m, 1H), 2.19-2.02 (m, 3H), 2.01-1.90 (m, 3H), 1.90-1.79 (m, 3H), 1.79-1.69 (m, 1H), 1.68-1.60 (m, 1H); ^{13}C NMR (101 MHz, $CDCl_3$) δ 170.9, 48.9, 46.6, 45.6, 37.4, 33.9, 32.7, 32.2, 30.2, 26.1, 24.4; HRMS (ESI) Calcd for $C_{11}H_{20}NOS$ $[M + H]^+$ 214.1260, found 214.1260.

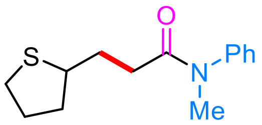

3q

***N*-Methyl-*N*-phenyl-3-(tetrahydrothiophen-2-yl)propanamide (3q)** Prepared according to the general procedure from **1a** (1 mL, 0.2 M) and **2q** (0.20 mmol) purified by column chromatography on silica gel with petroleum ether/ethyl acetate (10:1) to provide the title compound **3q** as a light yellow oil (29.9 mg, 60% yield); ^{1}H NMR (400 MHz, $CDCl_3$) δ 7.42 (t, *J* = 7.6 Hz, 2H), 7.34 (t, *J* = 7.3 Hz, 1H), 7.19 (dd, *J* = 7.4, 1.5 Hz, 2H), 3.26 (s, 4H), 2.79 (q, *J* = 5.6, 5.1 Hz, 2H), 2.23-2.07 (m, 2H), 2.07-1.93 (m, 3H), 1.90-1.82 (m, 1H), 1.77-1.63 (m, 1H), 1.55-1.44 (m, 1H); ^{13}C NMR (101 MHz, $CDCl_3$) δ 172.4, 144.0, 129.8, 127.8, 127.3, 48.5, 37.32, 37.26, 33.4, 33.2, 32.1, 30.1; HRMS (ESI) Calcd for $C_{14}H_{20}NOS$ $[M + H]^+$ 250.1260, found 250.1258.

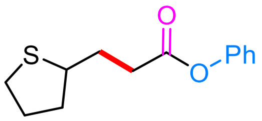

3r

Phenyl 3-(tetrahydrothiophen-2-yl)propanoate (3r)[1] Prepared according to the general procedure from **1a** (1 mL, 0.2 M) and **2r** (0.20 mmol) purified by column chromatography on silica gel with petroleum ether/ethyl acetate (100:1) to provide the title compound **3r** as a light yellow oil (28.8 mg, 61% yield); ^{1}H NMR (400 MHz, $CDCl_3$) δ 7.40-7.34 (m, 2H), 7.25-7.19 (m, 1H), 7.11-7.05 (m, 2H), 3.50-3.40 (m, 1H), 2.97-2.85 (m, 2H), 2.73-2.57 (m, 2H), 2.17-2.06 (m, 3H), 1.99-1.89 (m, 2H), 1.71-1.63 (m, 1H); ^{13}C NMR (101 MHz, $CDCl_3$) δ 171.7, 150.6, 129.4, 125.7, 121.53, 121.49, 48.2, 37.1, 33.5, 32.5, 32.3, 30.2; HRMS (ESI) Calcd for $C_{13}H_{17}O_2S$ $[M + H]^+$ 237.0944, found 237.0942.

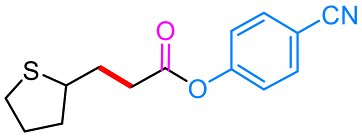

3s

4-Cyanophenyl 3-(tetrahydrothiophen-2-yl)propanoate (3s) Prepared according to the general procedure from **1a** (1 mL, 0.2 M) and **2s** (0.20 mmol) purified by column chromatography on silica gel with petroleum ether/ethyl acetate (100:1) to provide the title compound **3s** as light yellow oil (38.2 mg, 73% yield); ^{1}H NMR (400 MHz, $CDCl_3$) δ 7.72-7.65 (m, 2H), 7.27-7.21 (m, 2H), 3.54-3.35 (m, 1H), 2.95-2.84 (m, 2H), 2.78-2.57 (m, 2H), 2.20-2.05 (m, 3H), 2.01-1.88 (m, 2H), 1.70-1.63 (m, 1H); ^{13}C NMR (101 MHz, $CDCl_3$) δ 170.8, 153.8, 133.6, 122.6, 118.2, 109.6, 48.0, 37.1, 33.3, 32.3, 32.2, 30.1; HRMS (ESI) Calcd for $C_{14}H_{16}NO_2S$ $[M + H]^+$ 262.0896, found 262.0892.

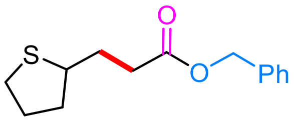

3t

Benzyl 3-(tetrahydrothiophen-2-yl)propanoate (3t)[1] Prepared according to the general procedure from **1a** (1 mL, 0.2 M) and **2t** (0.20 mmol) purified by column chromatography on silica gel with petroleum ether/ethyl acetate (100:1) to provide the title compound **3t** as a colorless oil (25.0 mg, 50% yield); ^{1}H NMR (400 MHz, $CDCl_3$) δ 7.41-7.27 (m, 5H), 5.11 (s, 2H), 3.40-3.31 (m, 1H), 2.90-2.82 (m, 2H), 2.53-2.34 (m, 2H), 2.11-1.99 (m, 3H), 1.94-1.77 (m, 2H), 1.62-1.53 (m, 1H); ^{13}C NMR (101 MHz, $CDCl_3$) δ 173.0, 135.9, 128.5, 128.2, 66.2, 48.2, 37.1, 33.4, 32.5, 32.2, 30.2; HRMS (ESI) Calcd for $C_{14}H_{19}O_2S$ $[M + H]^+$ 251.1100, found 251.1095.

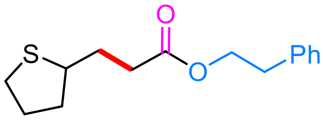

3u

Phenethyl 3-(tetrahydrothiophen-2-yl)propanoate (3u) Prepared according to the general procedure from **1a** (1 mL, 0.2 M) and **2u** (0.20 mmol) purified by column chromatography on silica gel with petroleum ether/ethyl acetate (100:1-50:1) to provide the title compound **3u** as a colorless oil (30.1 mg, 57% yield); ^{1}H NMR (400 MHz, $CDCl_3$) δ 7.33-7.27 (m, 2H), 7.25-

7.19 (m, 3H), 4.29 (t, J = 7.0 Hz, 2H), 3.36-3.27 (m, 1H), 2.93 (t, J = 7.1 Hz, 2H), 2.89-2.82 (m, 2H), 2.44-2.29 (m, 2H), 2.09-2.02 (m, 2H), 2.00-1.86 (m, 2H), 1.83-1.72 (m, 1H), 1.60-1.53 (m, 1H); ^{13}C NMR (101 MHz, $CDCl_3$) δ 173.1, 137.8, 128.9, 128.4, 126.5, 64.8, 48.2, 37.1, 35.1, 33.4, 32.5, 32.2, 30.1; HRMS (ESI) Calcd for $C_{15}H_{21}O_2S$ [M + H]$^+$ 265.1257, found 265.1251.

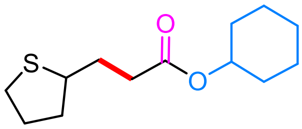

3v

Cyclohexyl 3-(tetrahydrothiophen-2-yl)propanoate (3v) Prepared according to the general procedure from **1a** (1 mL, 0.2 M) and **2v** (0.20 mmol) purified by column chromatography on silica gel with petroleum ether/ethyl acetate (100:1-50:1) to provide the title compound **3v** as a colorless oil (22.3 mg, 46% yield); ^{1}H NMR (400 MHz, $CDCl_3$) δ 4.80-4.71 (m, 1H), 3.41-3.30 (m, 1H), 2.90-2.83 (m, 2H), 2.46-2.29 (m, 2H), 2.13-2.04 (m, 2H), 2.02-1.88 (m, 2H), 1.87-1.77 (m, 3H), 1.76-1.68 (m, 2H), 1.63-1.51 (m, 2H), 1.43-1.32 (m, 4H), 1.30-1.21 (m, 1H); ^{13}C NMR (101 MHz, $CDCl_3$) δ 172.6, 72.6, 48.4, 37.1, 33.9, 32.7, 32.2, 31.62, 31.60, 30.2, 25.4, 23.7; HRMS (ESI) Calcd for $C_{13}H_{23}O_2S$ [M + H]$^+$ 243.1413, found 243.1408.

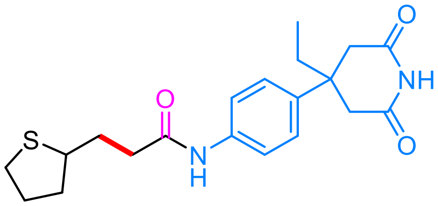

3w

***N*-(4-(4-Ethyl-2,6-dioxopiperidin-4-yl)phenyl)-3-(tetrahydrothiophen-2-yl)propanamide (3w)** Prepared according to the general procedure from **1a** (1 mL, 0.2 M) and **2w** (0.20 mmol) purified by column chromatography on silica gel with petroleum ether/ethyl acetate (10:1) to provide the title compound **3w** as a yellow solid (44.9 mg, 60% yield); m.p. 81.7-83.5 °C; ^{1}H NMR (400 MHz, $CDCl_3$) δ 8.50 (s, 1H), 7.85 (s, 1H), 7.53 (d, J = 8.3 Hz, 2H), 7.19 (d, J = 8.9 Hz, 2H), 3.45-3.33 (m, 1H), 2.93-2.83 (m, 2H), 2.62-2.47 (m, 2H), 2.45-2.33 (m, 3H), 2.26-2.16 (m, 2H), 2.15-2.05 (m, 2H), 2.04-1.99 (m, 1H), 1.96-1.88 (m, 2H), 1.85-1.76 (m, 1H), 1.67-1.55 (m, 1H), 0.86 (t, J = 7.4 Hz, 3H); ^{13}C NMR (101 MHz, $CDCl_3$) ^{13}C NMR (101 MHz, $CDCl_3$) δ 175.4, 172.6, 171.0, 137.4, 134.2, 126.7, 120.1, 50.6, 48.6, 37.3, 36.8, 33.1,

32.8, 32.2, 30.1, 29.2, 26.9, 9.0; HRMS (ESI) Calcd for $C_{20}H_{27}N_2O_3S$ [M + H]$^+$ 375.1737, found 375.1740.

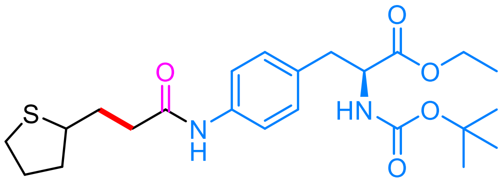

3x

Ethyl (2*S*)-2-((*tert*-butoxycarbonyl)amino)-3-(4-(3-(tetrahydrothiophen-2-yl)propanamido)phenyl)propanoate (3x) Prepared according to the general procedure from **1a** (1 mL, 0.2 M) and **2x** (0.20 mmol) purified by column chromatography on silica gel with petroleum ether/ethyl acetate (10:1) to provide the title compound **3x** as a dark red solid (51.4 mg, 57% yield); m.p. 89.7-91.9 °C; ^{1}H NMR (400 MHz, $CDCl_3$) δ 7.52 (s, 1H), 7.45 (d, *J* = 8.4 Hz, 2H), 7.07 (d, *J* = 8.1 Hz, 2H), 5.00 (d, *J* = 8.2 Hz, 1H), 4.52 (q, *J* = 6.6 Hz, 1H), 4.17-4.13 (m, 2H), 3.44-3.37 (m, 1H), 3.09-3.00 (m, 2H), 2.89-2.83 (m, 2H), 2.53-2.44 (m, 1H), 2.42-2.34 (m, 1H), 2.22-2.15 (m, 1H), 2.11-2.08 (m, 1H), 1.96-1.89 (m, 1H), 1.85-1.78 (m, 1H), 1.67-1.59 (m, 1H), 1.42 (s, 9H), 1.25 (t, *J* = 7.1 Hz, 3H); ^{13}C NMR (101 MHz, $CDCl_3$) δ 171.8, 170.7, 155.1, 136.9, 131.8, 129.8, 119.8, 79.9, 61.3, 60.4, 54.4, 48.6, 37.6, 37.3, 36.8, 33.1, 32.2, 30.1, 28.2, 21.0, 14.13, 14.10; HRMS (ESI) Calcd for $C_{23}H_{35}N_2O_5S$ [M + H]$^+$ 451.2261, found 451.2251.

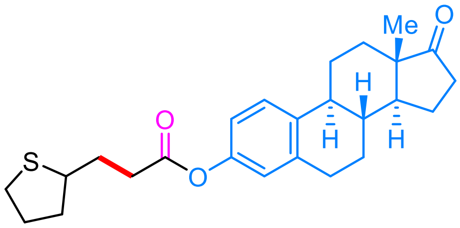

3y

(8*R*,9*S*,13*S*,14*S*)-13-Methyl-17-oxo-7,8,9,11,12,13,14,15,16,17-decahydro-6*H*-cyclopenta[*a*]phenanthren-3-yl 3-(tetrahydrothiophen-2-yl)propanoate (3y) Prepared according to the general procedure from **1a** (1 mL, 0.2 M) and **2y** (0.20 mmol) purified by column chromatography on silica gel with petroleum ether/ethyl acetate (20:1) to provide the title compound **3ya** as colorless oil (61.9 mg, 75% yield); ^{1}H NMR (700 MHz, $CDCl_3$) δ 7.28 (d, *J* = 8.5 Hz, 1H), 6.85 (dd, *J* = 8.5, 2.5 Hz, 1H), 6.80 (d, *J* = 2.6 Hz, 1H), 3.48-3.41 (m, 1H), 2.92-2.86 (m, 4H), 2.71-2.65 (m, 1H), 2.63-2.58 (m, 1H), 2.53-2.48 (m, 1H), 2.42-2.38 (m,

1H), 2.31-2.26 (m, 1H), 2.18-2.11 (m, 3H), 2.11-2.08 (m, 1H), 2.08-2.05 (m, 1H), 2.03-1.99 (m, 1H), 1.98-1.94 (m, 2H), 1.94-1.89 (m, 1H), 1.63-1.59 (m, 2H), 1.59-1.41 (m, 5H), 0.91 (s, 3H); ^{13}C NMR (175 MHz, $CDCl_3$) δ 220.8, 171.9, 148.5, 137.9, 137.3, 126.3, 121.5, 118.7, 50.4, 48.2, 47.9, 44.1, 37.9, 37.1, 35.8, 33.5, 32.5, 32.3, 31.5, 30.2, 29.3, 26.3, 25.7, 21.5, 13.8; HRMS (ESI) Calcd for $C_{25}H_{33}O_3S$ $[M + H]^+$ 413.2145, found 413.2148.

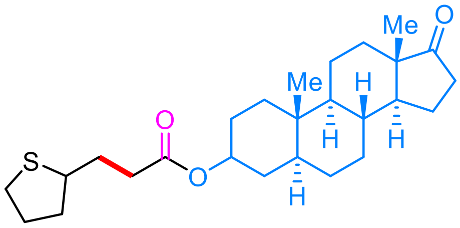

3z

(5*S*,8*R*,9*S*,10*S*,13*S*,14*S*)-10,13-Dimethyl-17-oxohexadecahydro-1*H*-cyclopenta[*a*]phenanthren-3-yl 3-(tetrahydrothiophen-2-yl)propanoate (3z) Prepared according to the general procedure from **1a** (1 mL, 0.2 M) and **2z** (0.20 mmol) purified by column chromatography on silica gel with petroleum ether/ethyl acetate (20:1) to provide the title compound **3z** as colorless oil (45.0 mg, 52% yield); ^{1}H NMR (400 MHz, $CDCl_3$) δ 4.75-4.63 (m, 1H), 3.40-3.28 (m, 1H), 2.93-2.80 (m, 2H), 2.48-2.24 (m, 3H), 2.13-2.01 (m, 3H), 2.01-1.86 (m, 3H), 1.83-1.76 (m, 4H), 1.74-1.71 (m, 1H), 1.68-1.58 (m, 3H), 1.57-1.46 (m, 3H), 1.38-1.19 (m, 7H), 1.08-0.95 (m, 2H), 0.86 (s, 3H), 0.85 (s, 3H), 0.76-0.66 (m, 1H); ^{13}C NMR (175 MHz, $CDCl_3$) δ 221.3, 172.7, 73.5, 54.3, 51.4, 48.3, 47.8, 44.6, 37.1, 36.7, 35.8, 35.6, 35.0, 33.93, 33.86, 32.7, 32.2, 31.5, 30.8, 30.2, 28.3, 27.40, 21.8, 20.4, 13.8, 12.2; HRMS (ESI) Calcd for $C_{26}H_{41}O_3S$ $[M + H]^+$ 433.2771, found 433.2776.

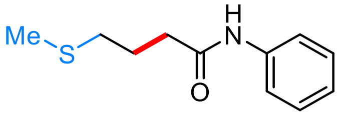

5a

4-(Methylthio)-*N*-phenylbutanamide (5a) Prepared according to the general procedure from **4a** (1 mL, 0.2 M) and **2a** (0.20 mmol) purified by column chromatography on silica gel with petroleum ether/ethyl acetate (10:1) to provide the title compound **5a** as a white solid (30.1 mg, 72% yield); m.p. 56.4-58.0 °C; ^{1}H NMR (400 MHz, $CDCl_3$) δ 7.51 (d, J = 7.9 Hz, 3H), 7.31 (t, J = 7.9 Hz, 2H), 7.10 (t, J = 7.4 Hz, 1H), 2.59 (t, J = 6.9 Hz, 2H), 2.49 (t, J = 7.2 Hz, 2H), 2.09 (d, J = 4.3 Hz, 3H), 2.07-1.99 (m, 2H); ^{13}C NMR (101 MHz, $CDCl_3$) δ 170.7, 137.8, 128.9, 124.2, 119.8, 35.8, 33.4, 24.2, 15.2; HRMS (ESI) Calcd for $C_{11}H_{16}NOS$ $[M + H]^+$ 210.0947, found 210.0949.

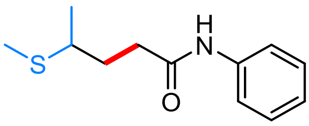

5b

4-(Ethylthio)-*N*-phenylbutanamide (5b) Prepared according to the general procedure from **4b** (1 mL, 0.2 M) and **2a** (0.20 mmol) purified by column chromatography on silica gel with petroleum ether/ethyl acetate (10:1) to provide the title compound **5b** as a colorless oil (30.3 mg, 68% yield); ^{1}H NMR (400 MHz, $CDCl_3$) δ 7.51 (d, *J* = 7.9 Hz, 2H), 7.36 (s, 1H), 7.32 (t, *J* = 7.9 Hz, 2H), 7.10 (t, *J* = 7.4 Hz, 1H), 2.83-2.70 (m, 1H), 2.58-2.46 (m, 2H), 2.07 (s, 3H), 2.05-1.98 (m, 1H), 1.92-1.81 (m, 1H), 1.32 (d, *J* = 6.8 Hz, 3H); ^{13}C NMR (101 MHz, $CDCl_3$) δ 170.8, 137.9, 129.0, 124.2, 119.7, 40.6, 34.9, 31.3, 21.1, 12.4; HRMS (ESI) Calcd for $C_{12}H_{18}NOS$ $[M + H]^+$ 224.1104, found 224.1107.

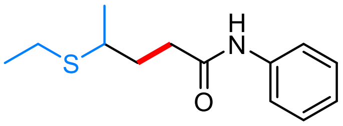

5c

4-(Ethylthio)-*N*-phenylpentanamide (5c) Prepared according to the general procedure from **4c** (1 mL, 0.2 M) and **2a** (0.20 mmol) purified by column chromatography on silica gel with petroleum ether/ethyl acetate (10:1) to provide the title compound **5c** as colorless oil (23.3 mg, 49% yield); ^{1}H NMR (400 MHz, $CDCl_3$) δ 7.51 (d, *J* = 7.9 Hz, 2H), 7.47 (s, 1H), 7.31 (t, *J* = 7.9 Hz, 2H), 7.10 (t, *J* = 7.4 Hz, 1H), 2.93-2.80 (m, 1H), 2.64-2.46 (m, 4H), 2.11-1.94 (m, 1H), 1.93-1.81 (m, 1H), 1.32 (d, *J* = 6.7 Hz, 3H), 1.25 (t, *J* = 7.4 Hz, 3H); ^{13}C NMR (101 MHz, $CDCl_3$) δ 170.9, 137.9, 129.0, 124.2, 119.7, 39.2, 34.8, 32.0, 23.9, 21.7, 14.9; HRMS (ESI) Calcd for $C_{13}H_{20}NOS$ $[M + H]^+$ 238.1260, found 238.1262.

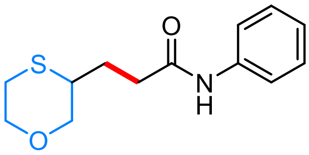

5d

3-(1,4-Oxathian-3-yl)-*N*-phenylpropanamide (5d) Prepared according to the general procedure from **4d** (1 mL, 0.2 M) and **2a** (0.20 mmol) purified by column chromatography on silica gel with petroleum ether/ethyl acetate (10:1) to provide the title compound **5d** as a colorless oil (25.1 mg, 50% yield); ^{1}H NMR (400 MHz, $CDCl_3$) δ 7.50 (d, *J* = 8.0 Hz, 2H), 7.39-7.26 (m, 3H), 7.11 (t, *J* = 7.4 Hz, 1H), 4.14-3.96 (m, 2H), 3.82-3.68 (m, 1H), 3.54 (dd, *J*

= 11.7, 8.1 Hz, 1H), 2.93-2.83 (m, 1H), 2.79-2.69 (m, 1H), 2.65-2.56 (m, 1H), 2.56-2.42 (m, 2H), 2.12-2.01 (m, 1H), 1.91-1.77 (m, 1H); ^{13}C NMR (101 MHz, $CDCl_3$) δ 170.2, 137.8, 129.0, 124.3, 119.8, 73.8, 68.5, 38.8, 34.3, 26.8, 26.3; HRMS (ESI) Calcd for $C_{13}H_{18}NO_2S$ $[M + H]^+$ 252.1053, found 252.1054.

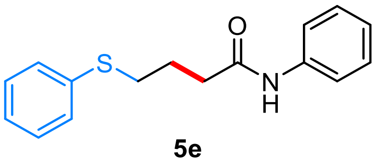

5e

***N*-Phenyl-4-(phenylthio)butanamide (5e)** Prepared according to the general procedure from **4e** (1 mL, 0.2 M) and **2a** (0.20 mmol) purified by column chromatography on silica gel with petroleum ether/ethyl acetate (10:1) to provide the title compound **5e** as light yellow solid (28.2 mg, 52% yield); m.p. 61.2-63.1 °C; ^{1}H NMR (400 MHz, $CDCl_3$) δ 7.48 (d, *J* = 8.0 Hz, 2H), 7.35 (t, *J* = 7.0 Hz, 3H), 7.32-7.27 (m, 3H), 7.23-7.07 (m, 3H), 3.04 (t, *J* = 6.8 Hz, 2H), 2.52 (t, *J* = 7.1 Hz, 2H), 2.14-2.00 (m, 2H); ^{13}C NMR (101 MHz, $CDCl_3$) δ 170.3, 137.7, 135.9, 129.4, 129.0, 126.2, 124.3, 120.6, 119.8, 35.7, 33.0, 24.6; HRMS (ESI) Calcd for $C_{16}H_{18}NOS$ $[M + H]^+$ 272.1104, found 272.1107.

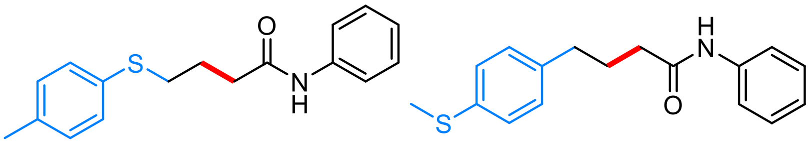

5f **5f'**

***N*-Phenyl-4-(*p*-tolylthio)butanamide (5f) and 4-(4-(Methylthio)phenyl)-*N*-phenylbutanamide (5f')** Prepared according to the general procedure from **4f** (1 mL, 0.2 M) and **2a** (0.20 mmol) purified by column chromatography on silica gel with petroleum ether/ethyl acetate (10:1) to provide the title compound **5f** as a light yellow solid (34.2 mg, 60% yield, rr = 1:1); m.p. 73.4-75.1 °C; ^{1}H NMR (400 MHz, $CDCl_3$) δ 7.47 (d, *J* = 8.0 Hz, 2H), 7.34-7.26 (m, 4H), 7.21 (s, 1H), 7.09 (d, *J* = 8.0 Hz, 3H), 2.98 (t, *J* = 6.8 Hz, 2H), 2.50 (t, *J* = 7.1 Hz, 2H), 2.31 (s, 3H), 2.11-1.97 (m, 2H); ^{13}C NMR (101 MHz, $CDCl_3$) δ 170.4, 137.8, 136.4, 132.0, 130.3, 129.9, 129.0, 124.3, 119.8, 35.7, 33.7, 24.7, 21.0; HRMS (ESI) Calcd for $C_{17}H_{20}NOS$ $[M + H]^+$ 286.1260, found 286.1262.

(5f'): a light yellow solid; m.p. 56.4-57.9 °C; ^{1}H NMR (400 MHz, $CDCl_3$) δ 7.49 (d, *J* = 8.0 Hz, 2H), 7.34-7.28 (m, 2H), 7.23-7.17 (m, 2H), 7.17-7.03 (m, 4H), 2.68 (t, *J* = 7.4 Hz, 2H), 2.47 (s, 3H), 2.33 (t, *J* = 7.4 Hz, 2H), 2.12-1.99 (m, 2H); ^{13}C NMR (101 MHz, $CDCl_3$) δ

170.8, 138.4, 135.7, 129.1, 129.0, 127.1, 124.2, 119.7, 36.7, 34.6, 26.7, 16.2; HRMS (ESI) Calcd for $C_{17}H_{20}NOS$ $[M + H]^+$ 286.1260, found 286.1259.

6. Reference

[1] E. Alfonzo, S. M. Hande, *ACS Catal*. 2020, *10*, 12590-12595.

7. NMR Spectra

^{1}H NMR (400 MHz, CDCl3) and ^{13}C NMR (100 MHz, CDCl3) spectrum of 3a

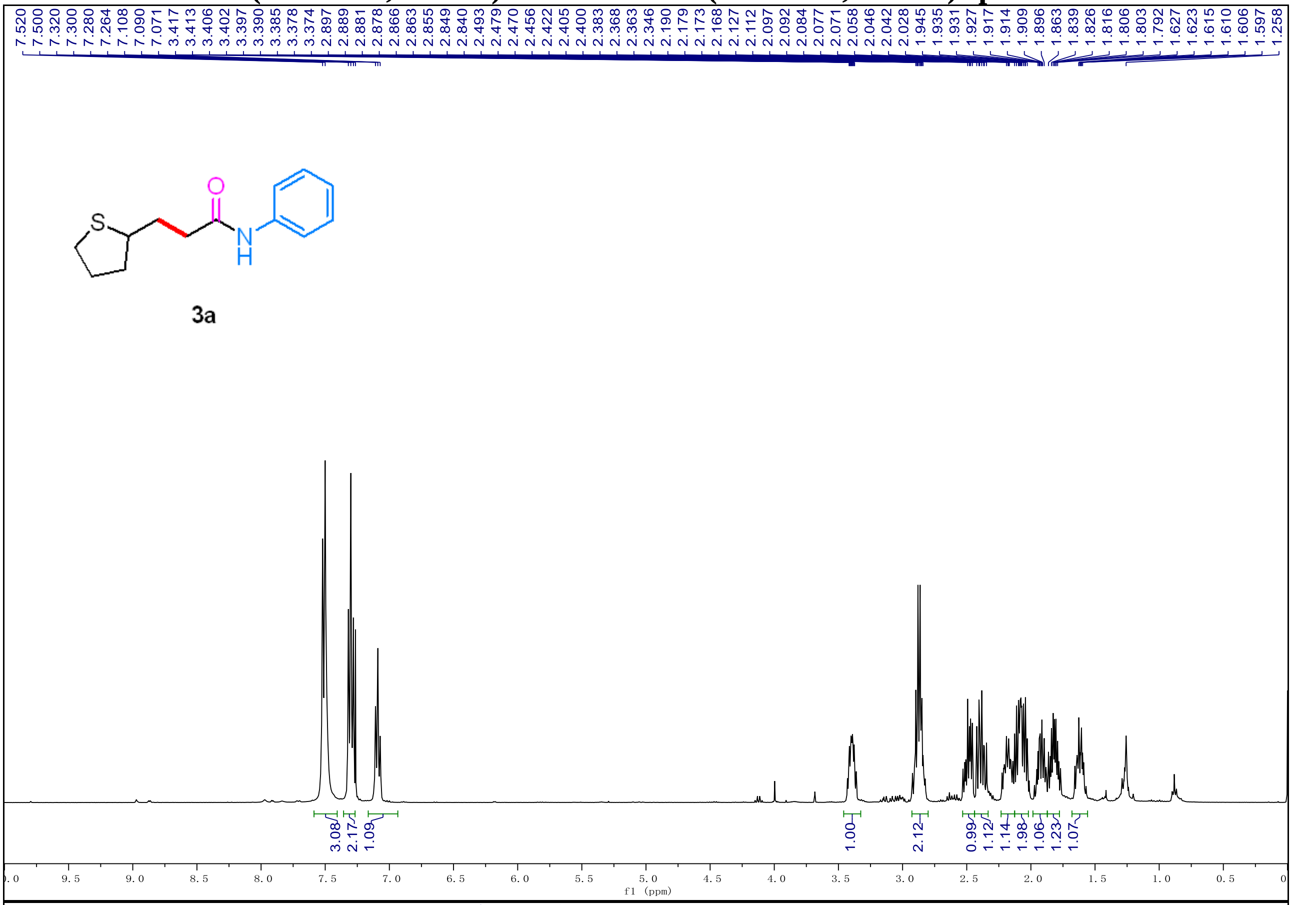

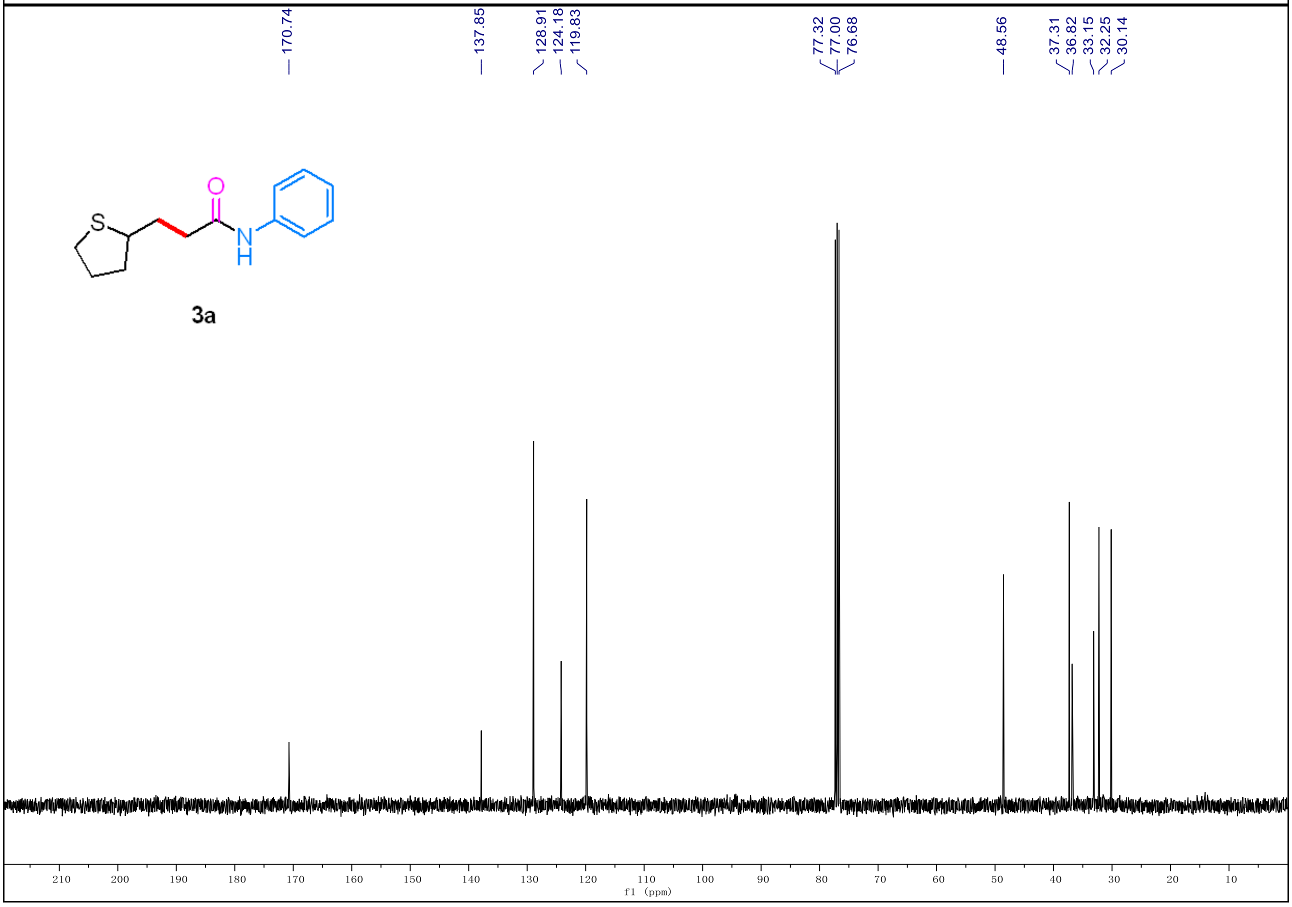

^{1}H NMR (400 MHz, CDCl3) and ^{13}C NMR (100 MHz, CDCl3) spectrum of 3b

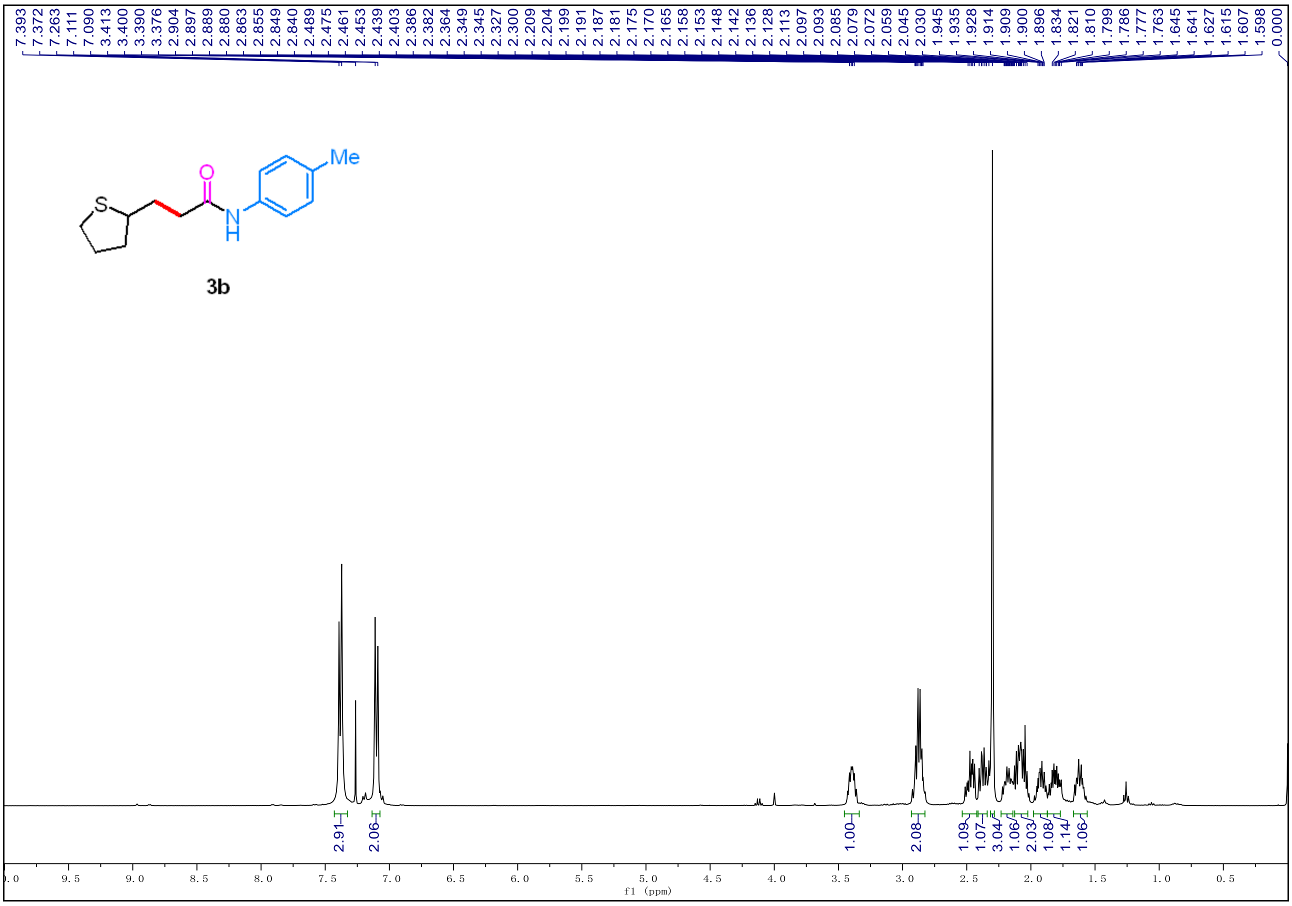

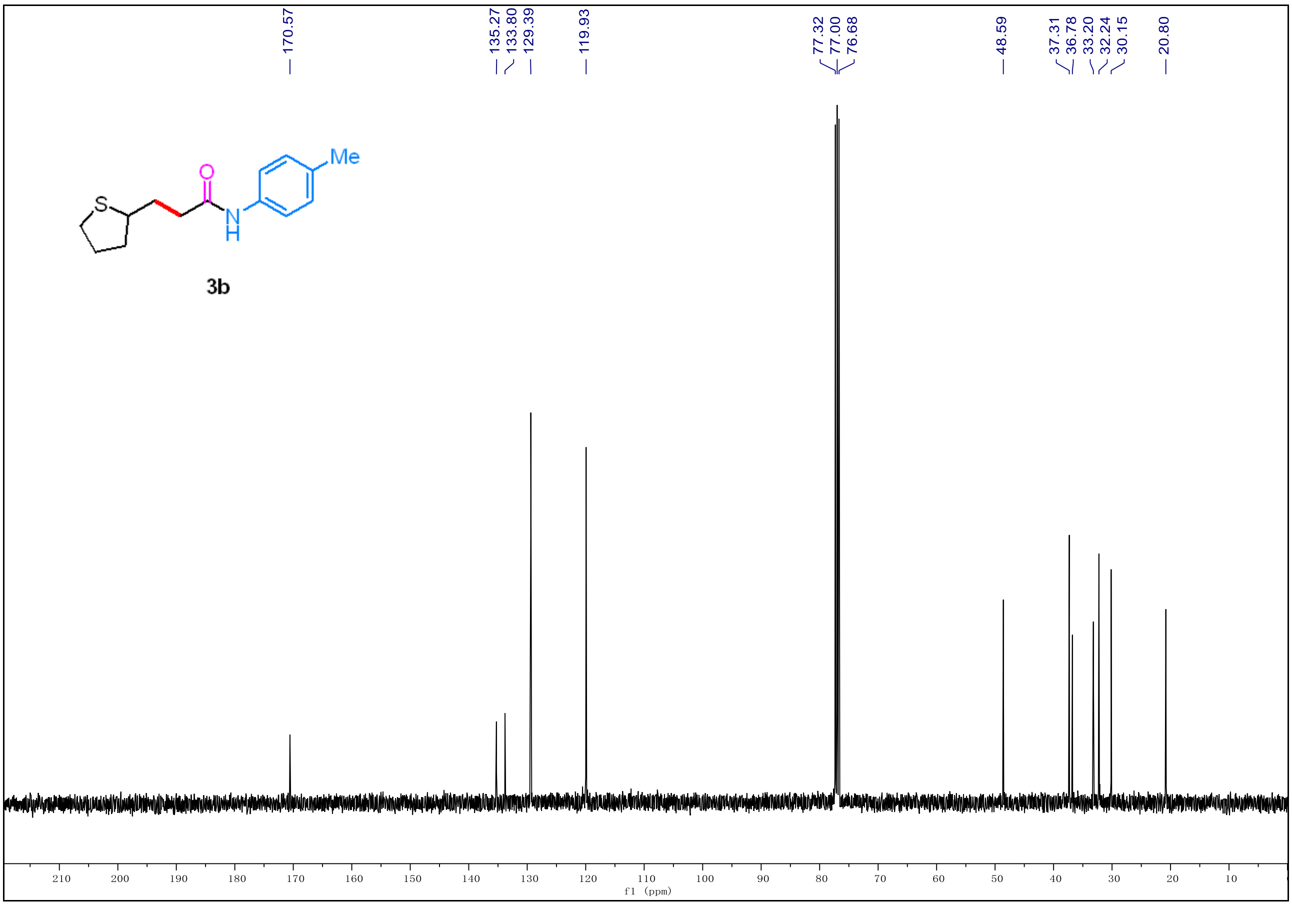

^{1}H NMR (400 MHz, $CDCl_3$) and ^{13}C NMR (100 MHz, $CDCl_3$) spectrum of 3c

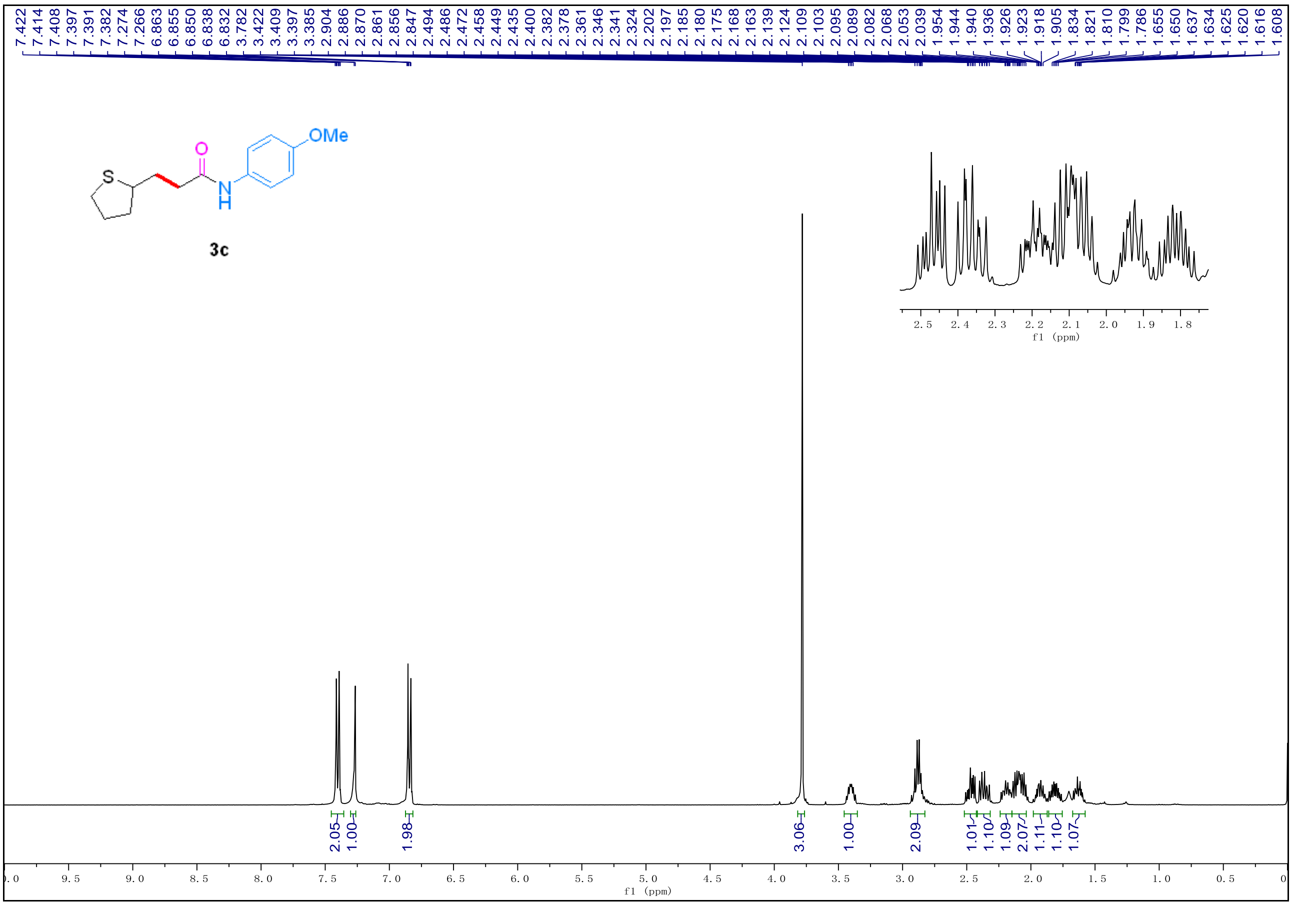

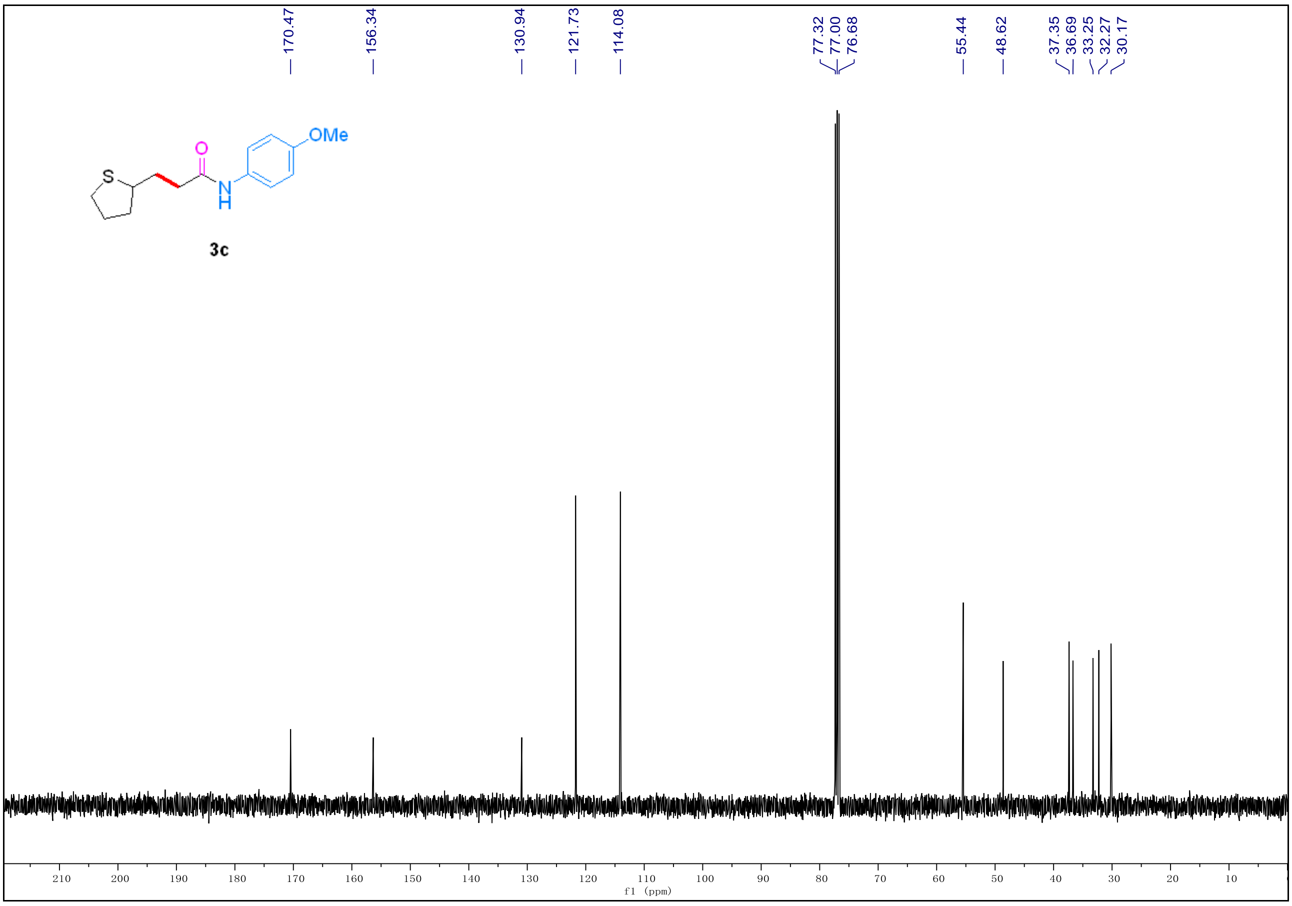

^{1}H NMR (400 MHz, $CDCl_3$) and ^{13}C NMR (100 MHz, $CDCl_3$) spectrum of 3d

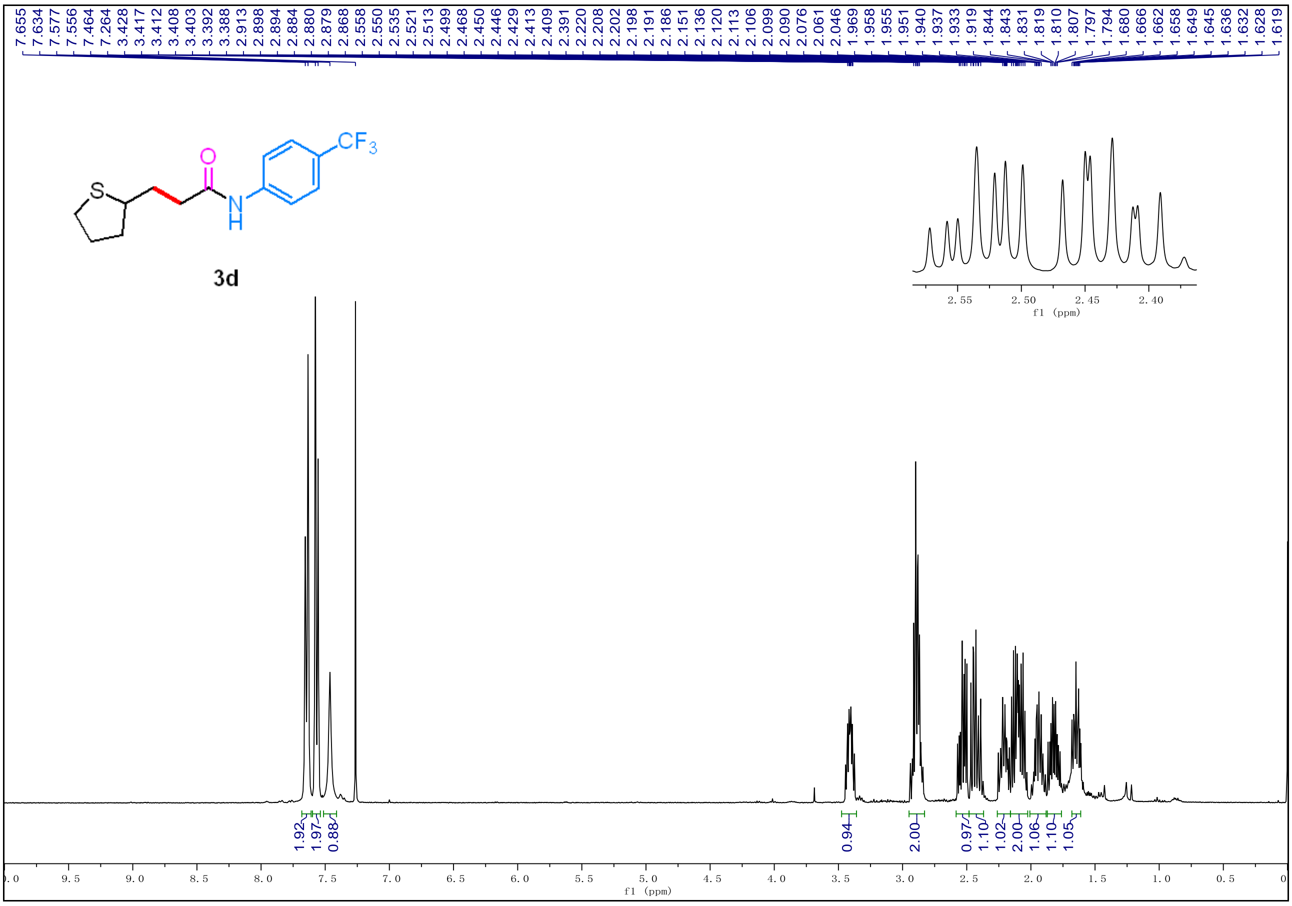

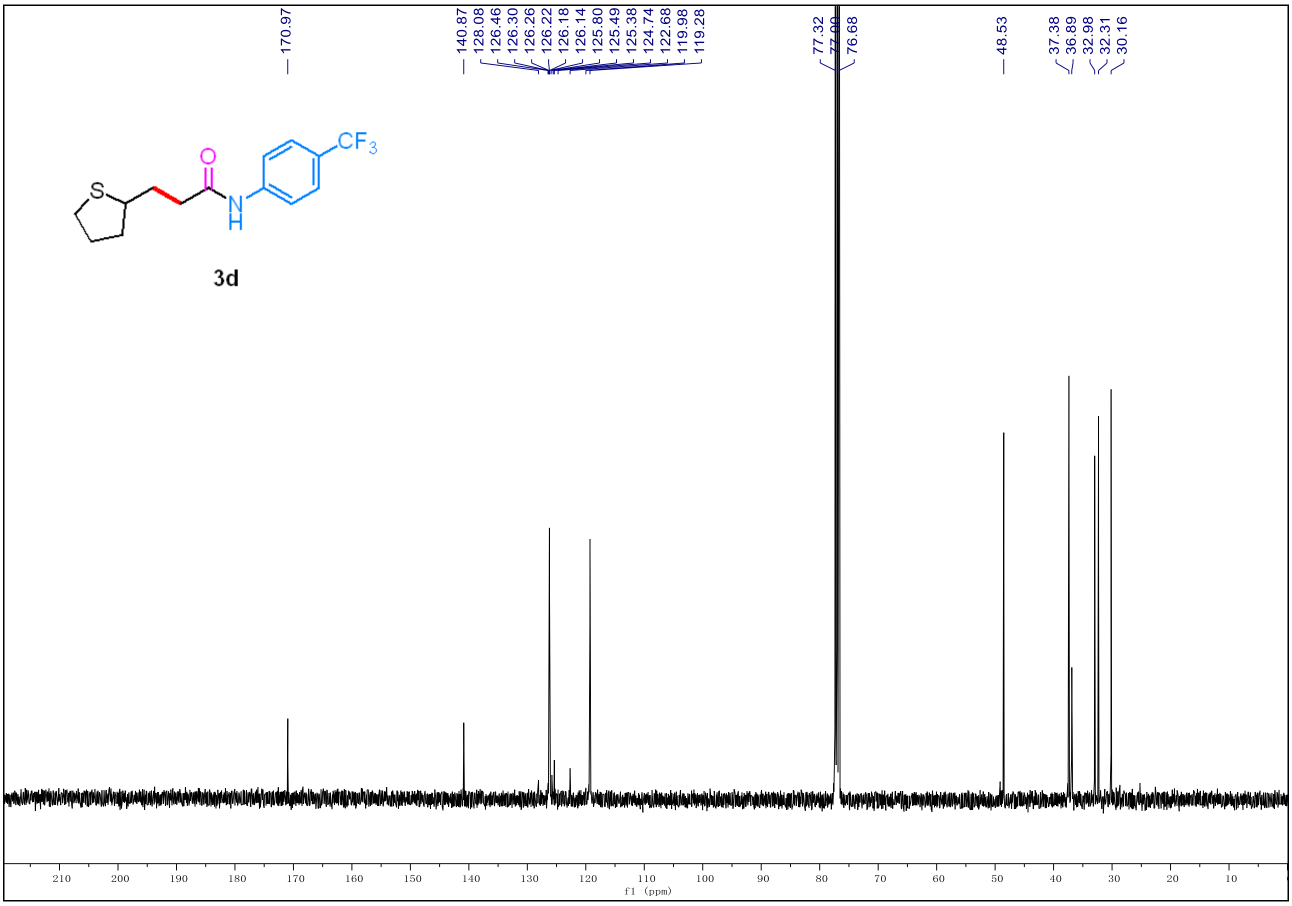

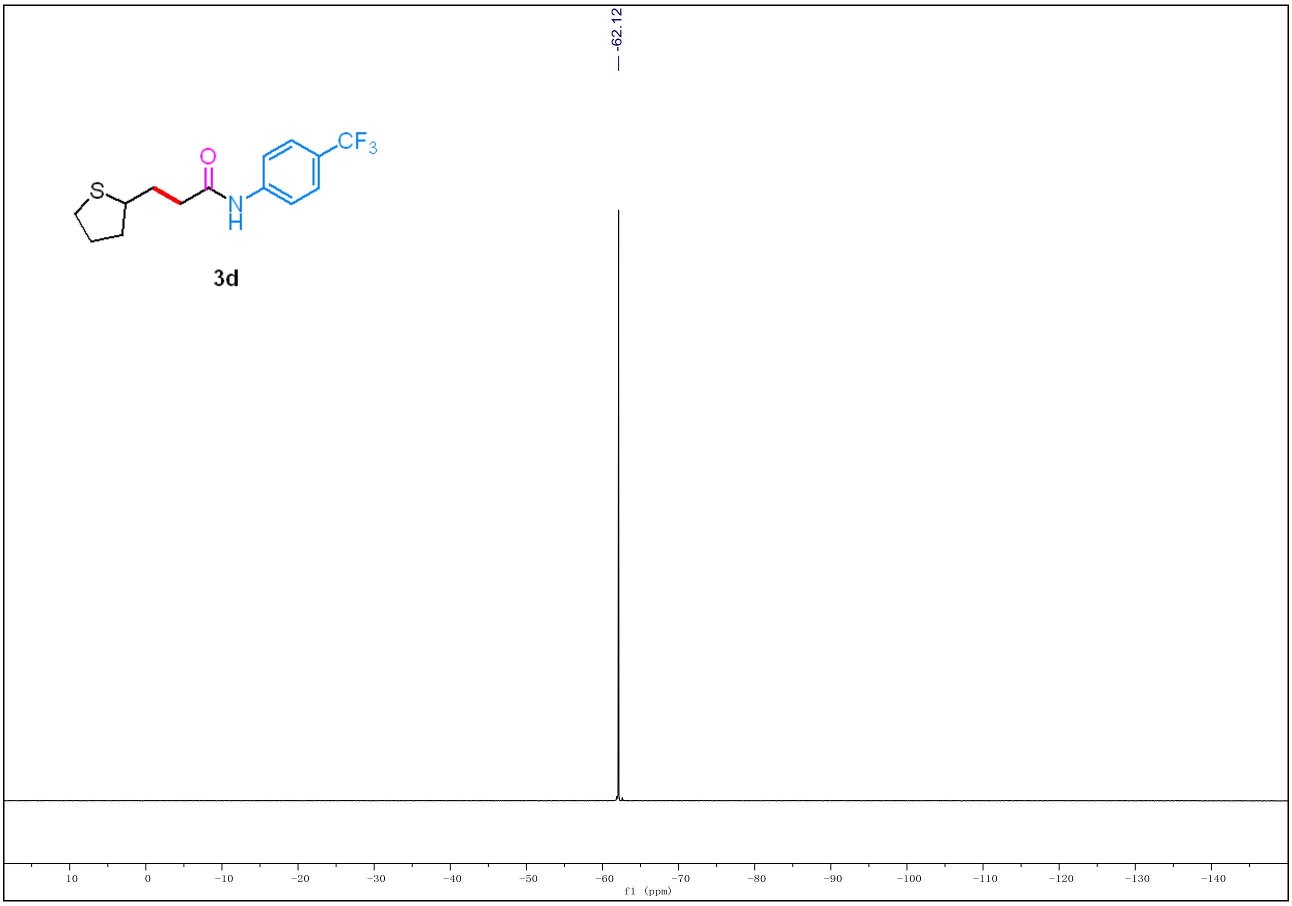
-62.12
S
O
N
H
CF3
3d
10
0
-10
-20
-30
-40
-50
-60
-70
-80
-90
-100
-110
-120
-130
-140
f1 (ppm)

^{1}H NMR (400 MHz, $CDCl_3$) and ^{13}C NMR (100 MHz, $CDCl_3$) spectrum of 3e

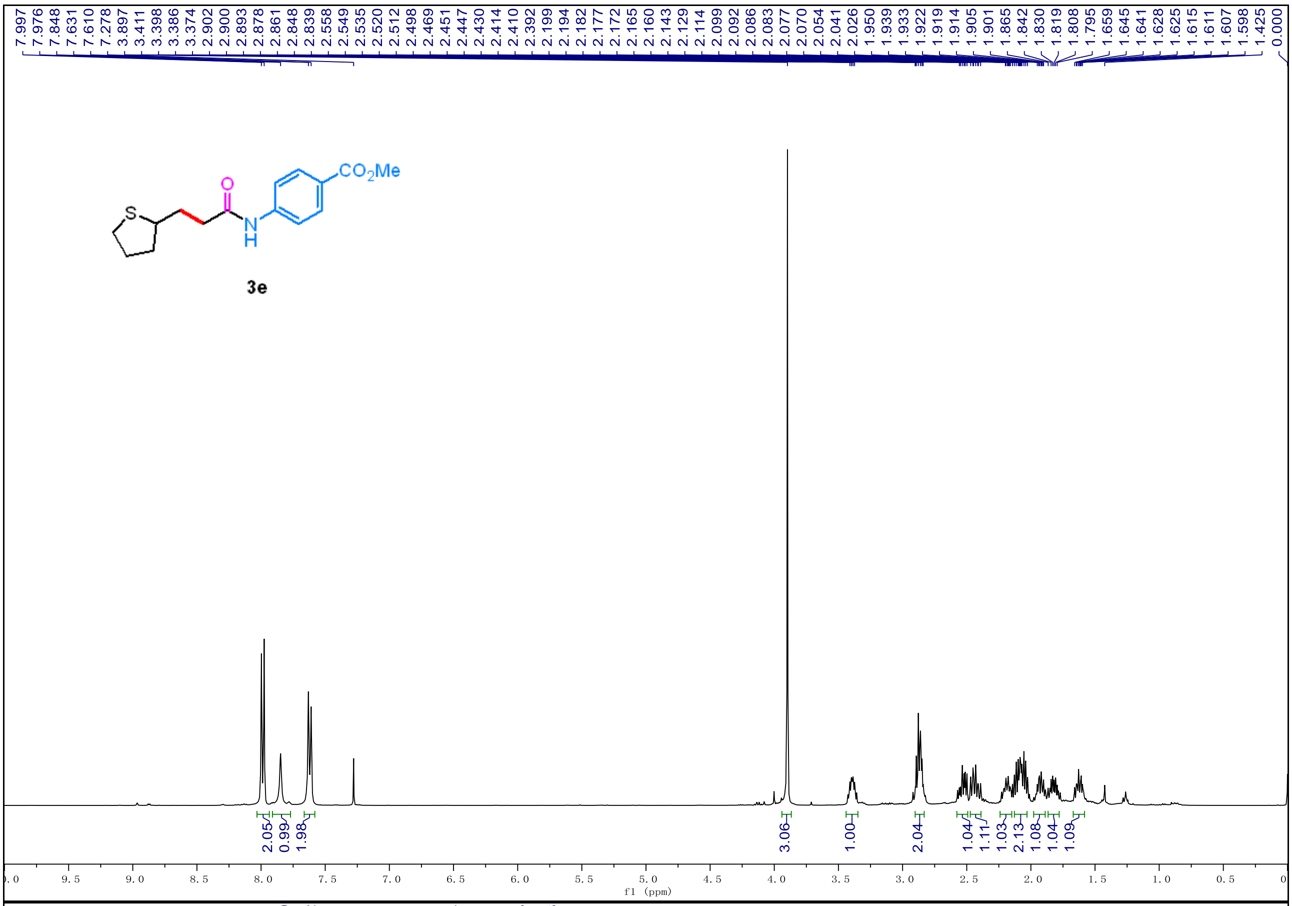

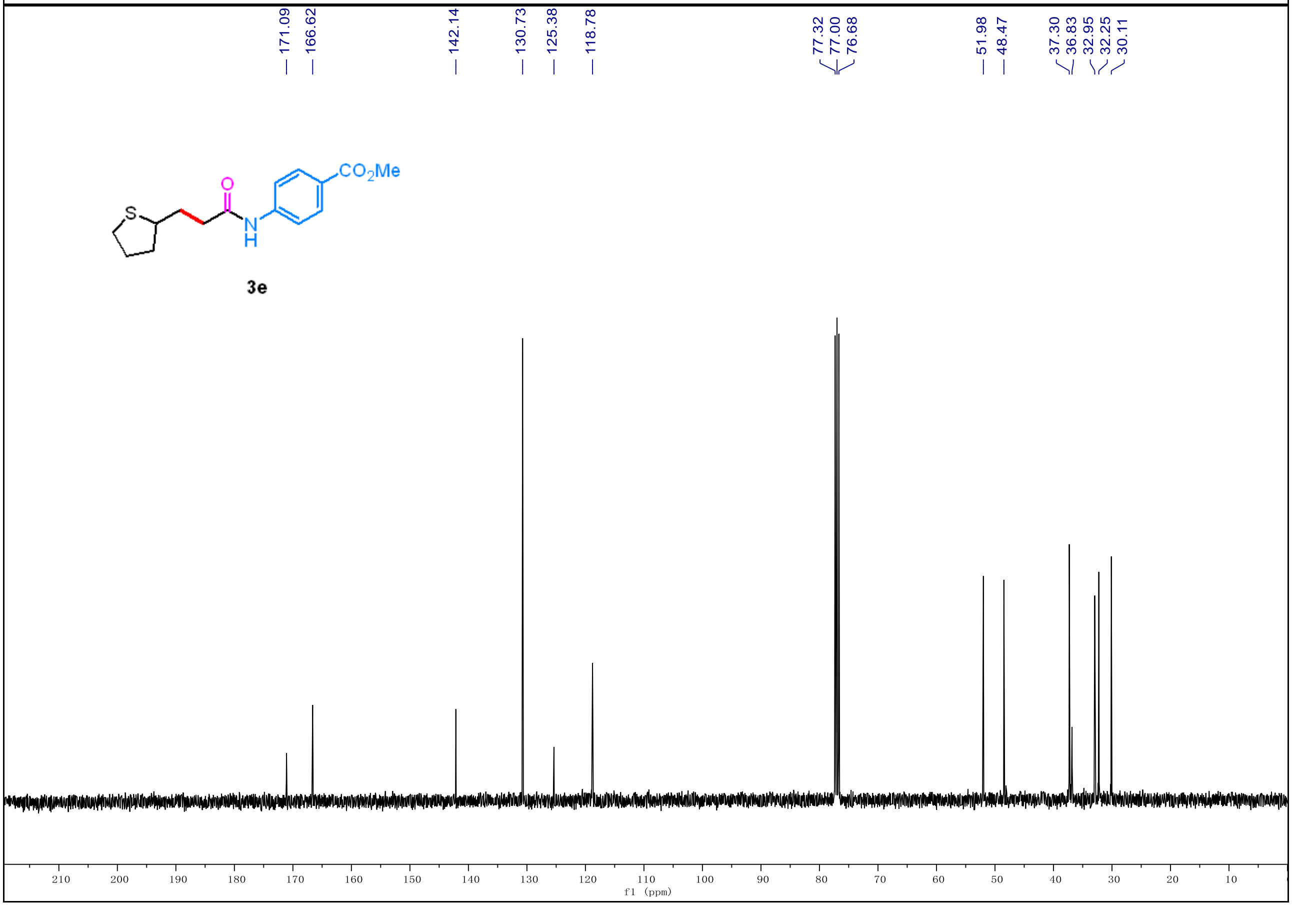

^{1}H NMR (400 MHz, $CDCl_3$) and ^{13}C NMR (100 MHz, $CDCl_3$) spectrum of 3f

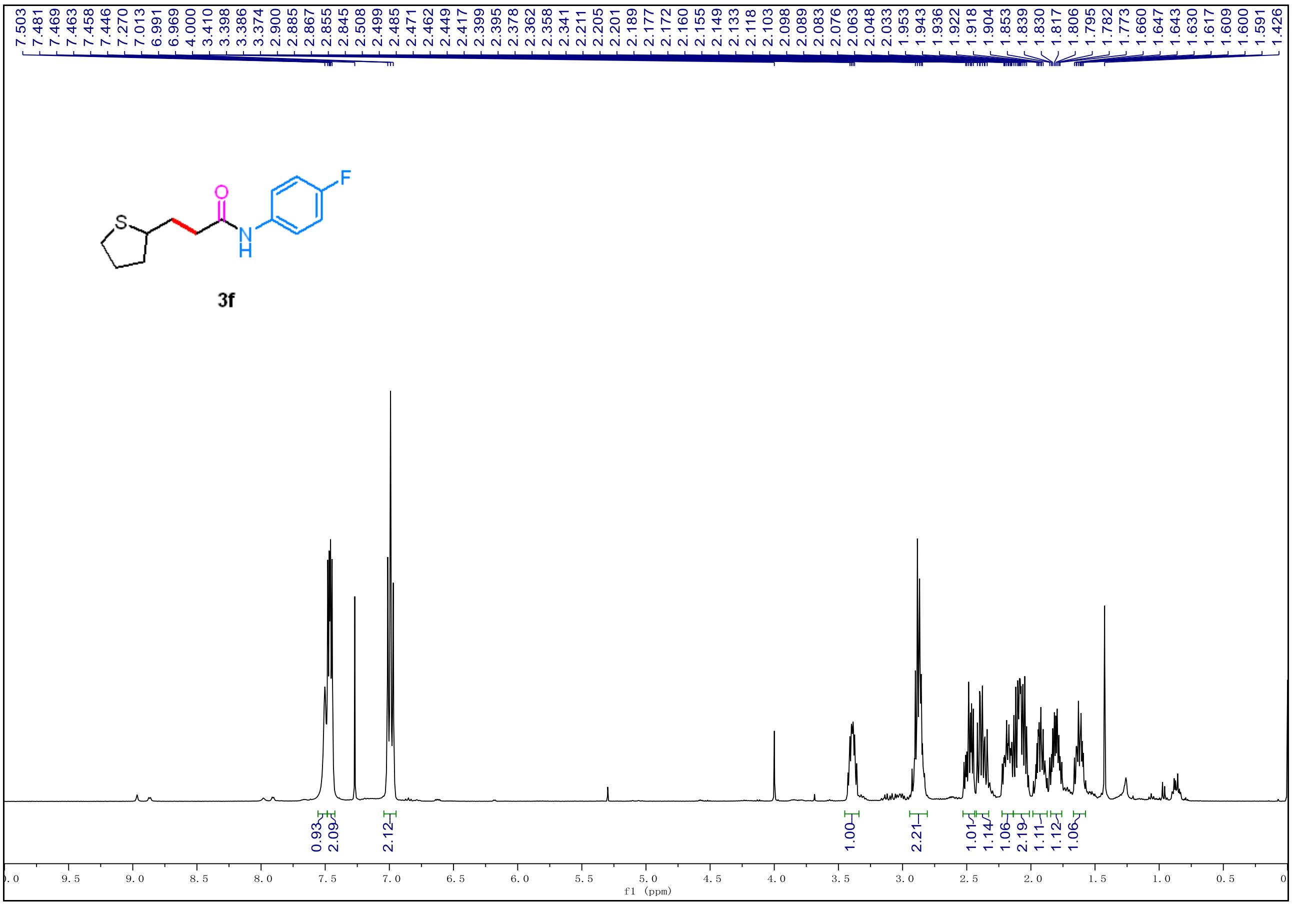

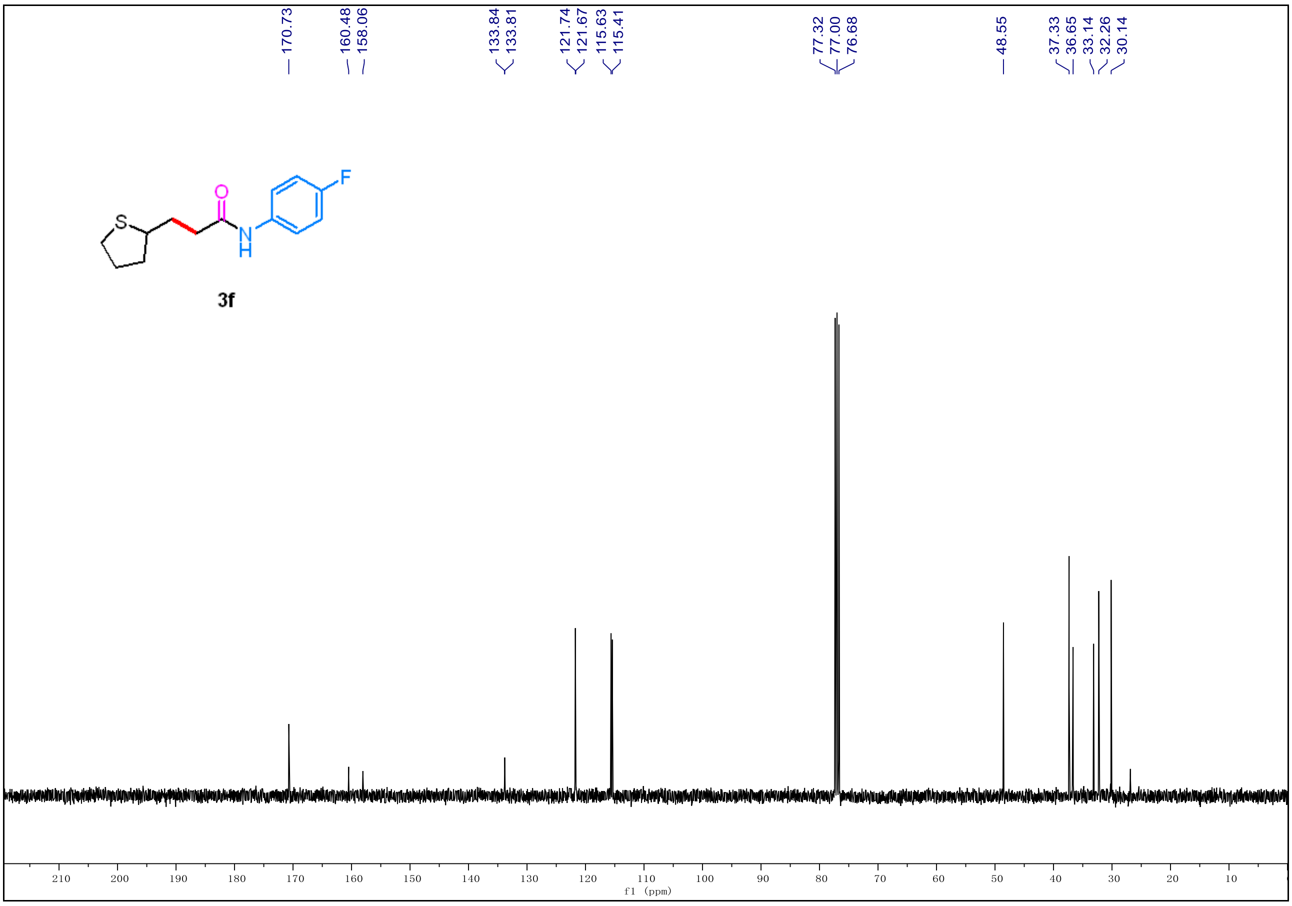

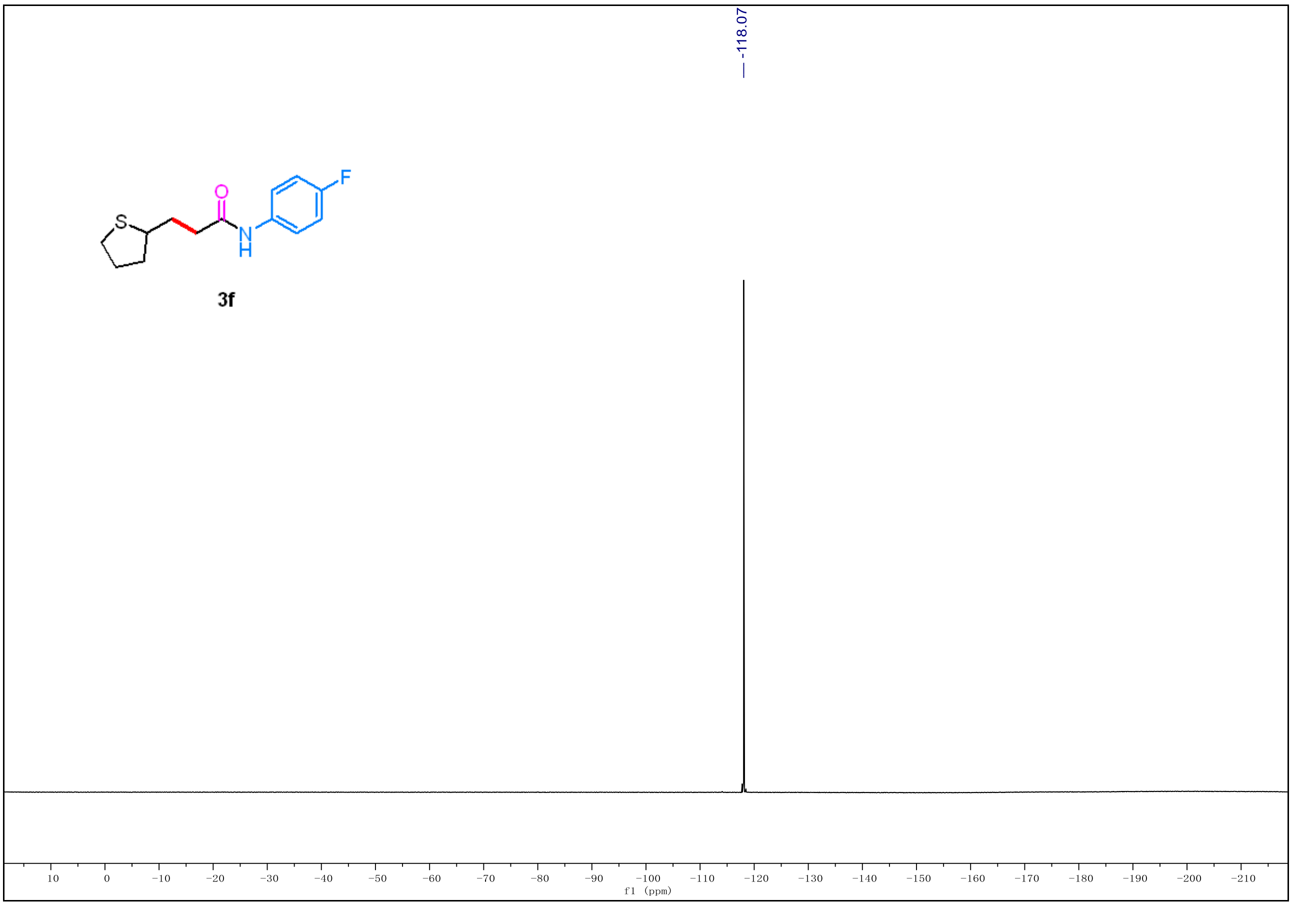

-118.07
3f
S
O
N
H
F
10
0
-10
-20
-30
-40
-50
-60
-70
-80
-90
-100
-110
-120
-130
-140
-150
-160
-170
-180
-190
-200
-210
f1 (ppm)

^{1}H NMR (400 MHz, CDCl₃) and ^{13}C NMR (100 MHz, CDCl₃) spectrum of 3g

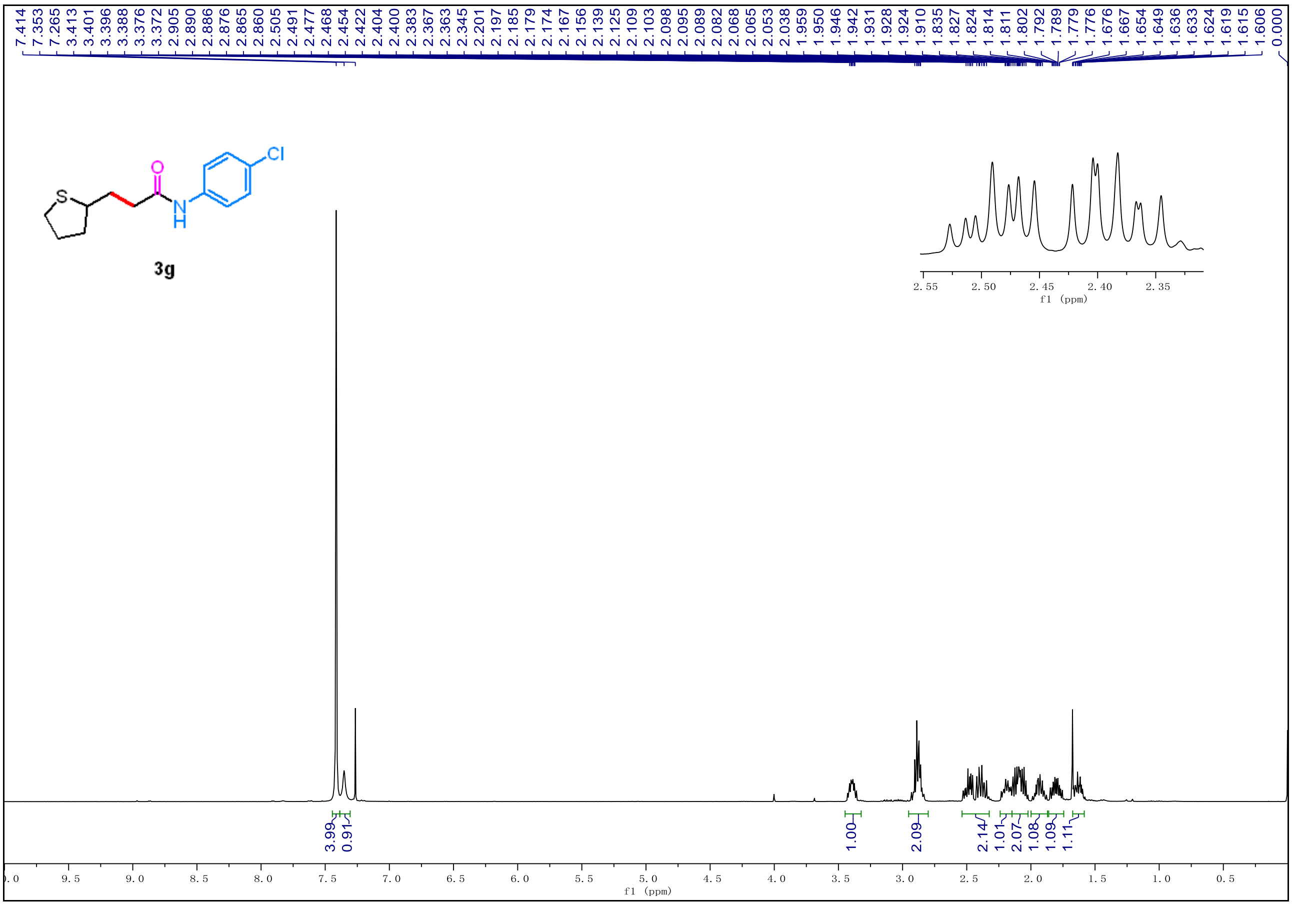

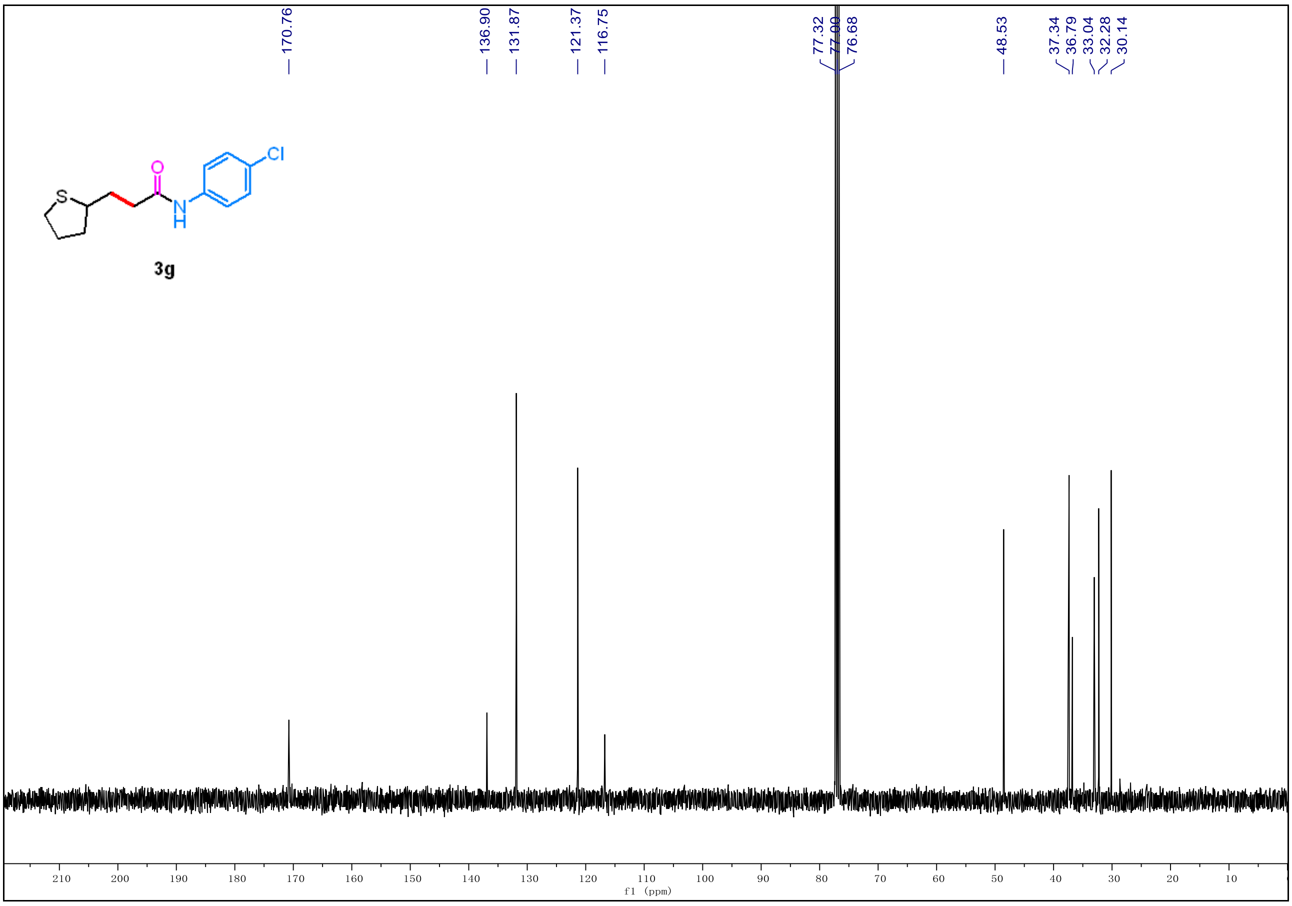

^{1}H NMR (400 MHz, CDCl$_3$) and ^{13}C NMR (100 MHz, CDCl$_3$) spectrum of 3h

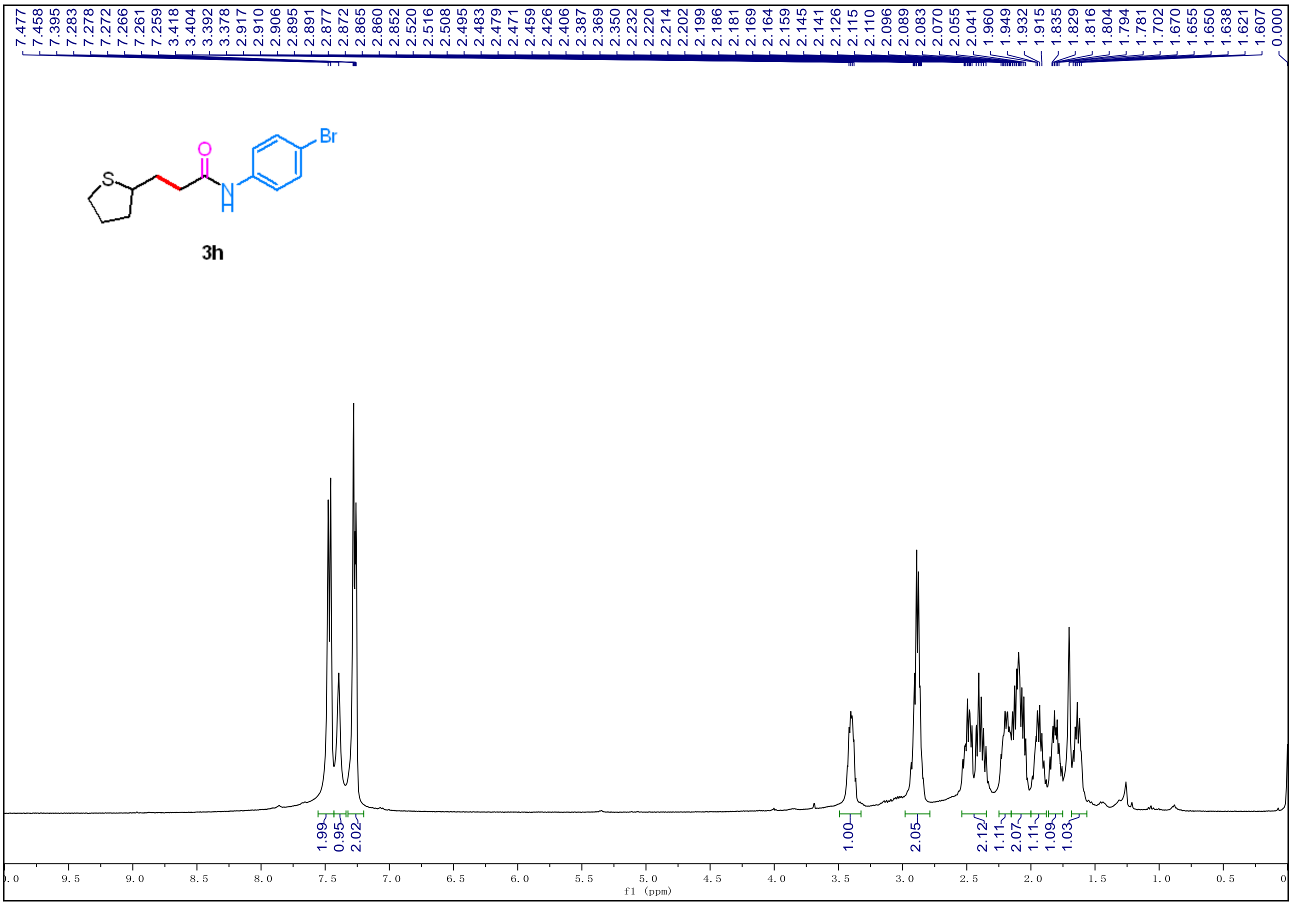

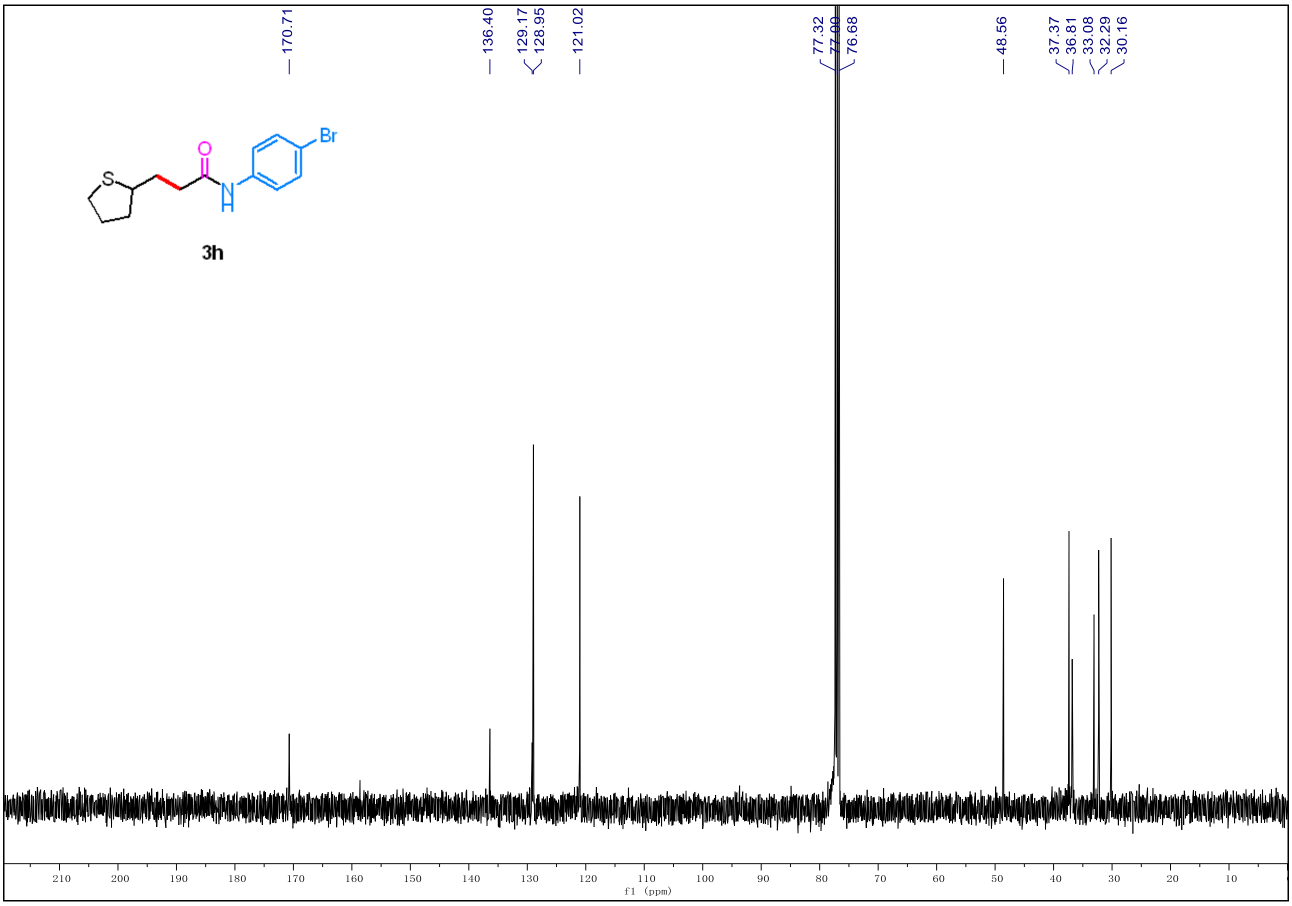

^{1}H NMR (400 MHz, $CDCl_3$) and ^{13}C NMR (100 MHz, $CDCl_3$) spectrum of 3i

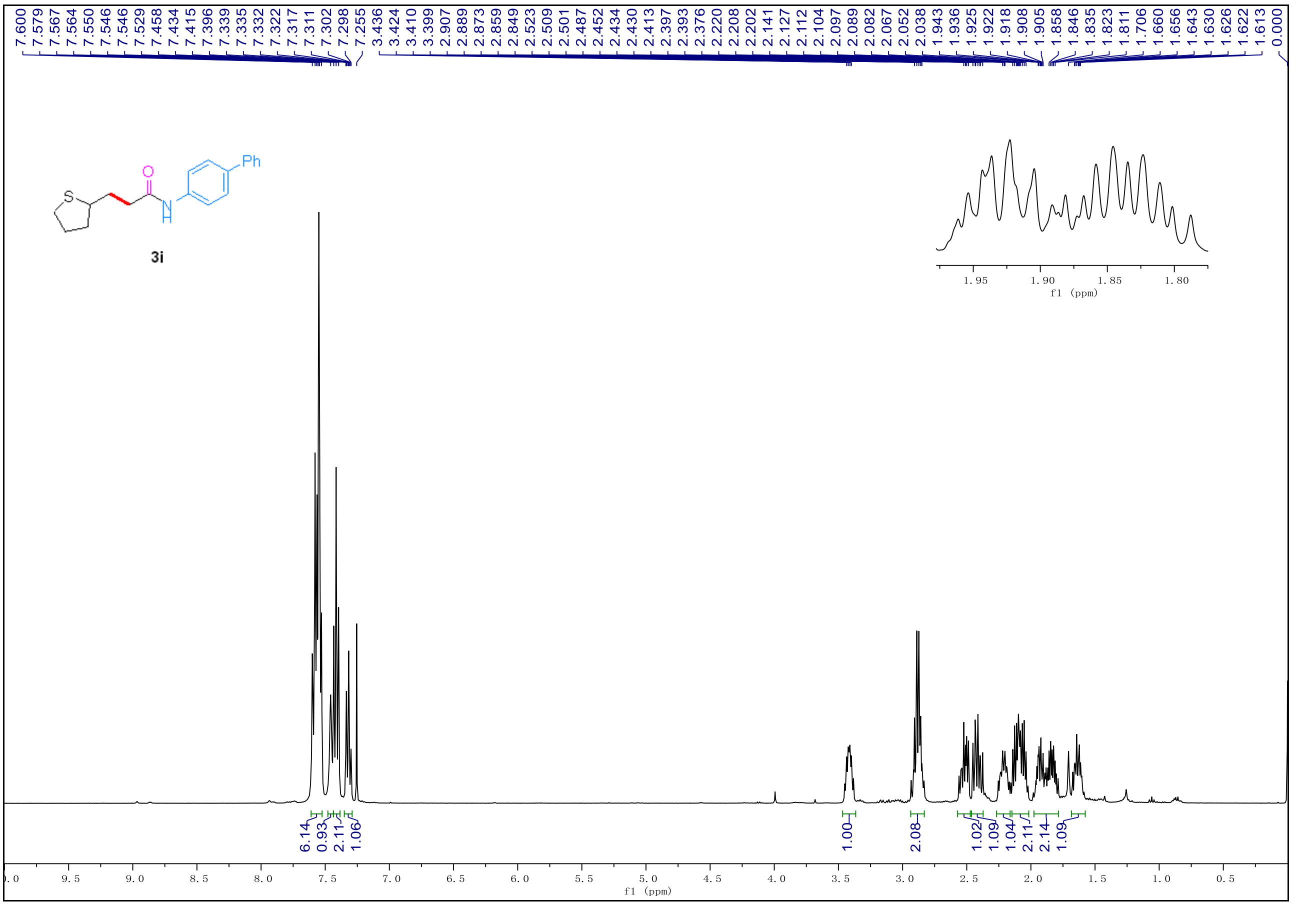

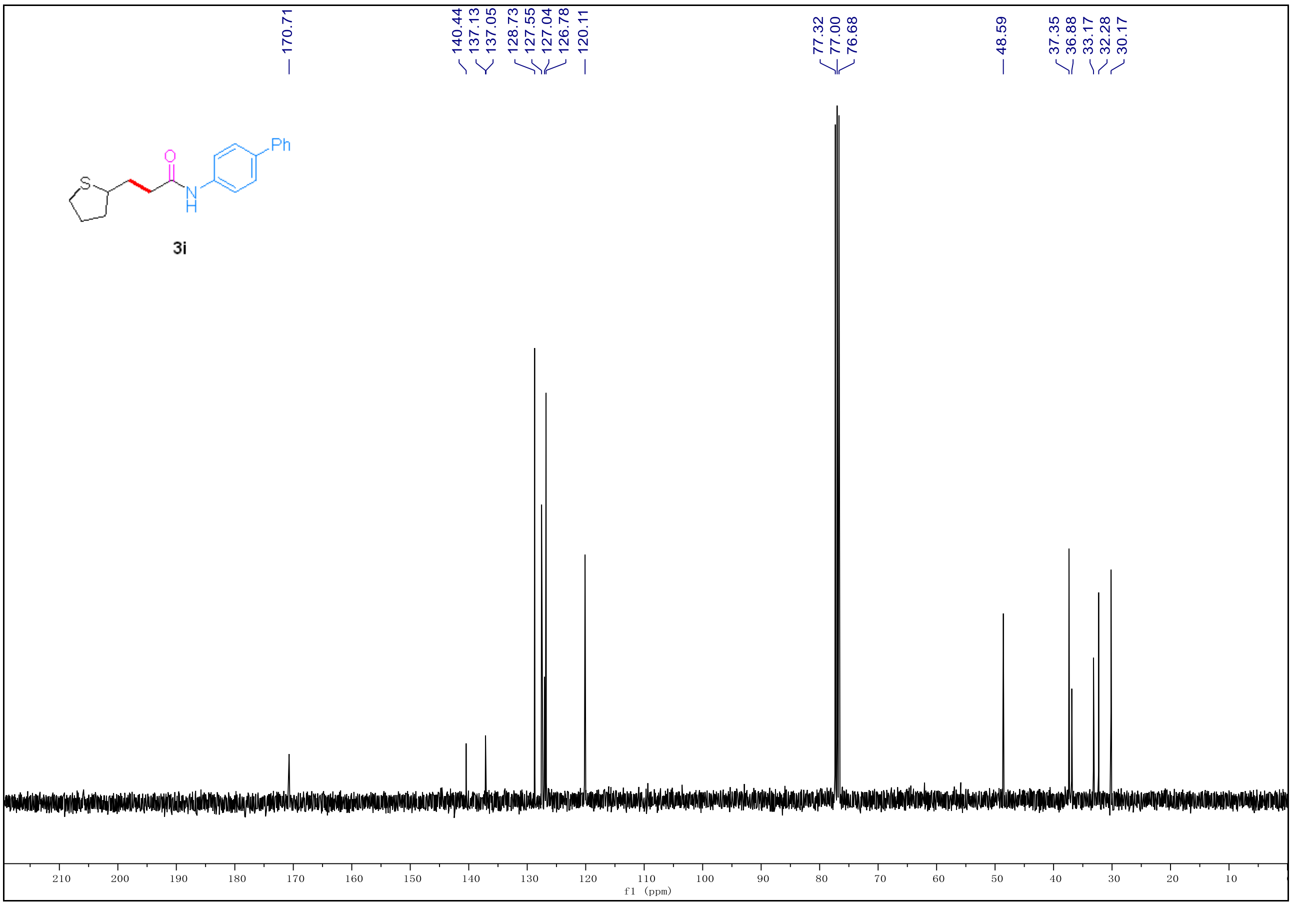

^{1}H NMR (400 MHz, $CDCl_3$) and ^{13}C NMR (100 MHz, $CDCl_3$) spectrum of 3j

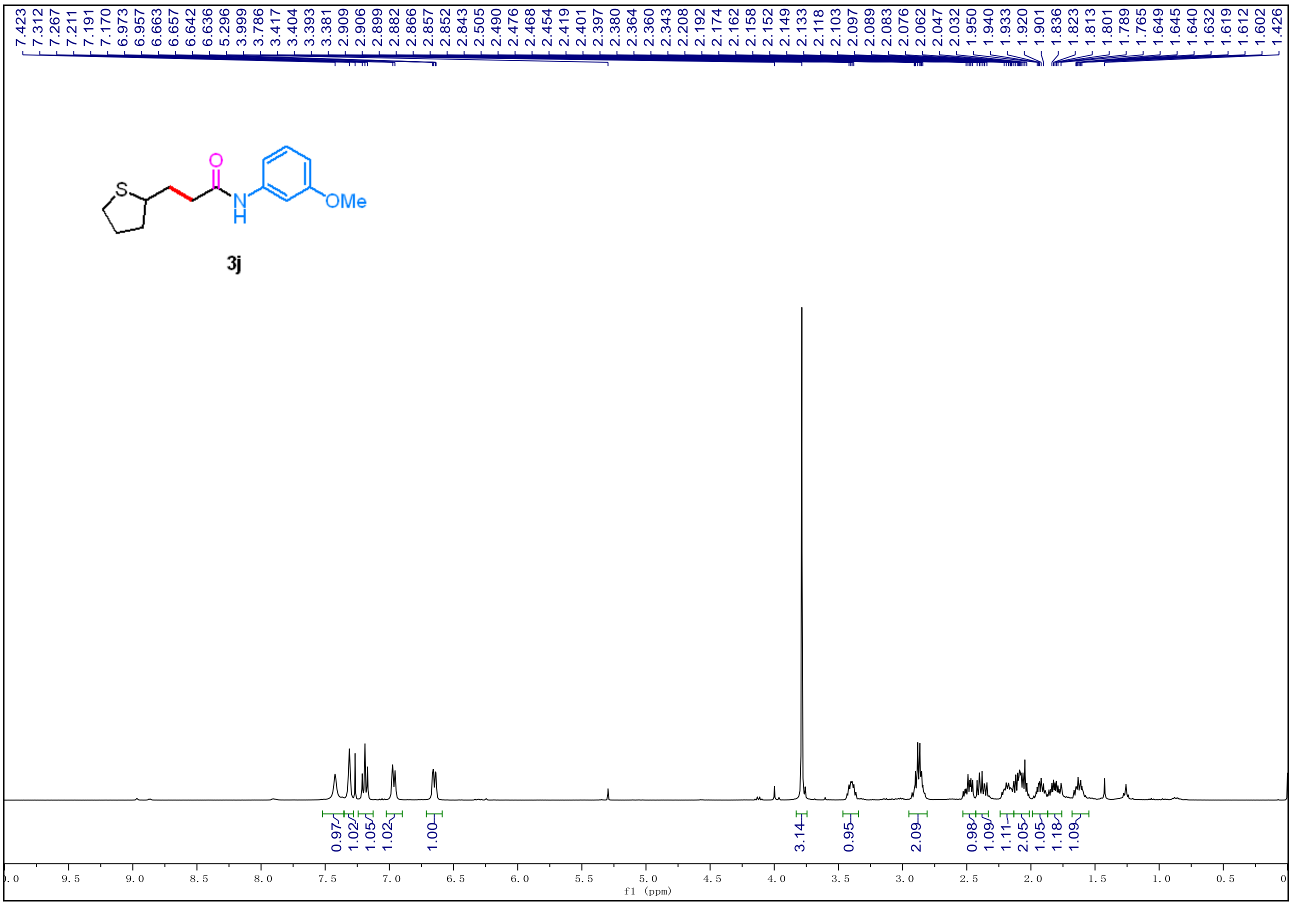

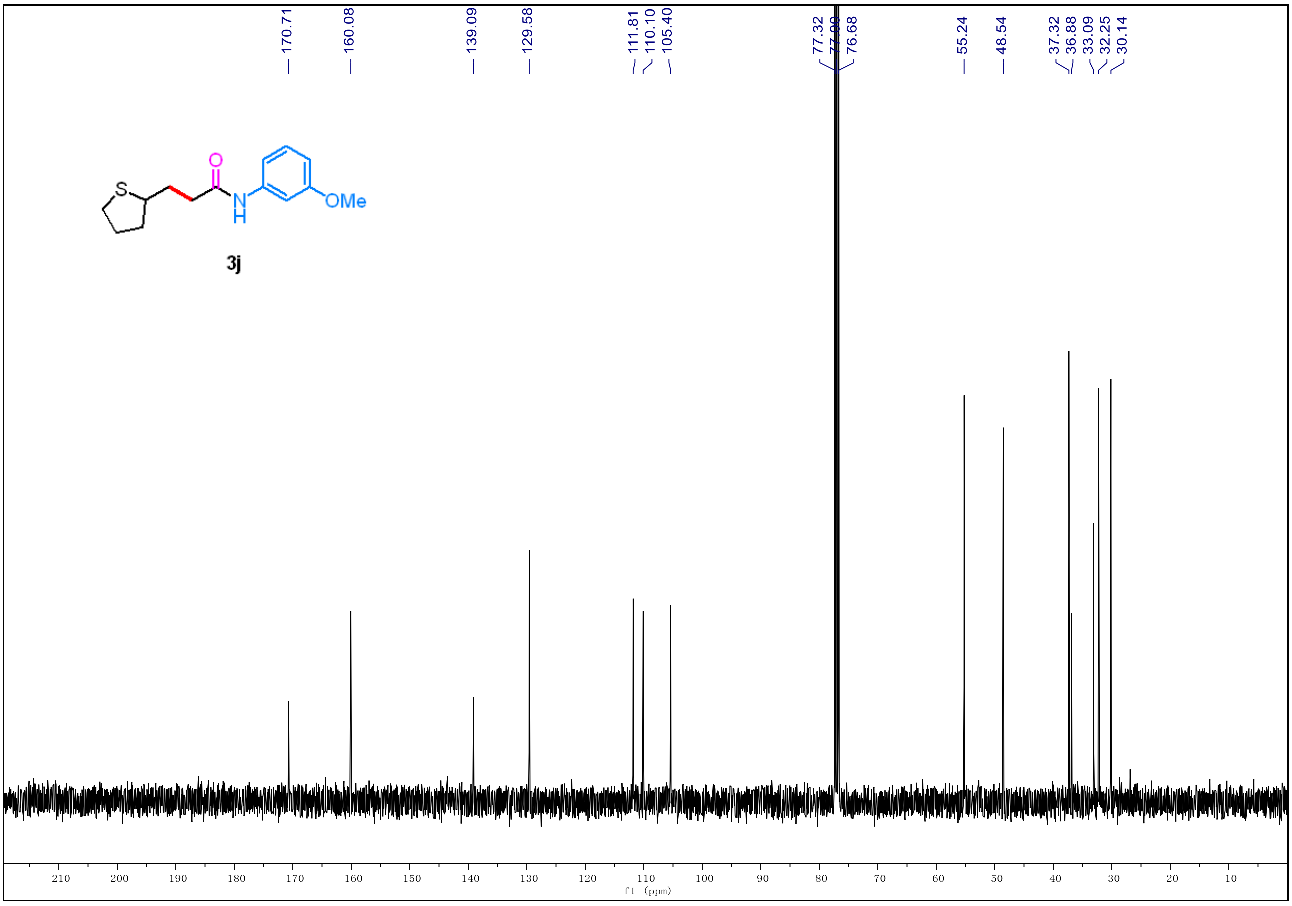

^{1}H NMR (400 MHz, $CDCl_3$) and ^{13}C NMR (100 MHz, $CDCl_3$) spectrum of 3k

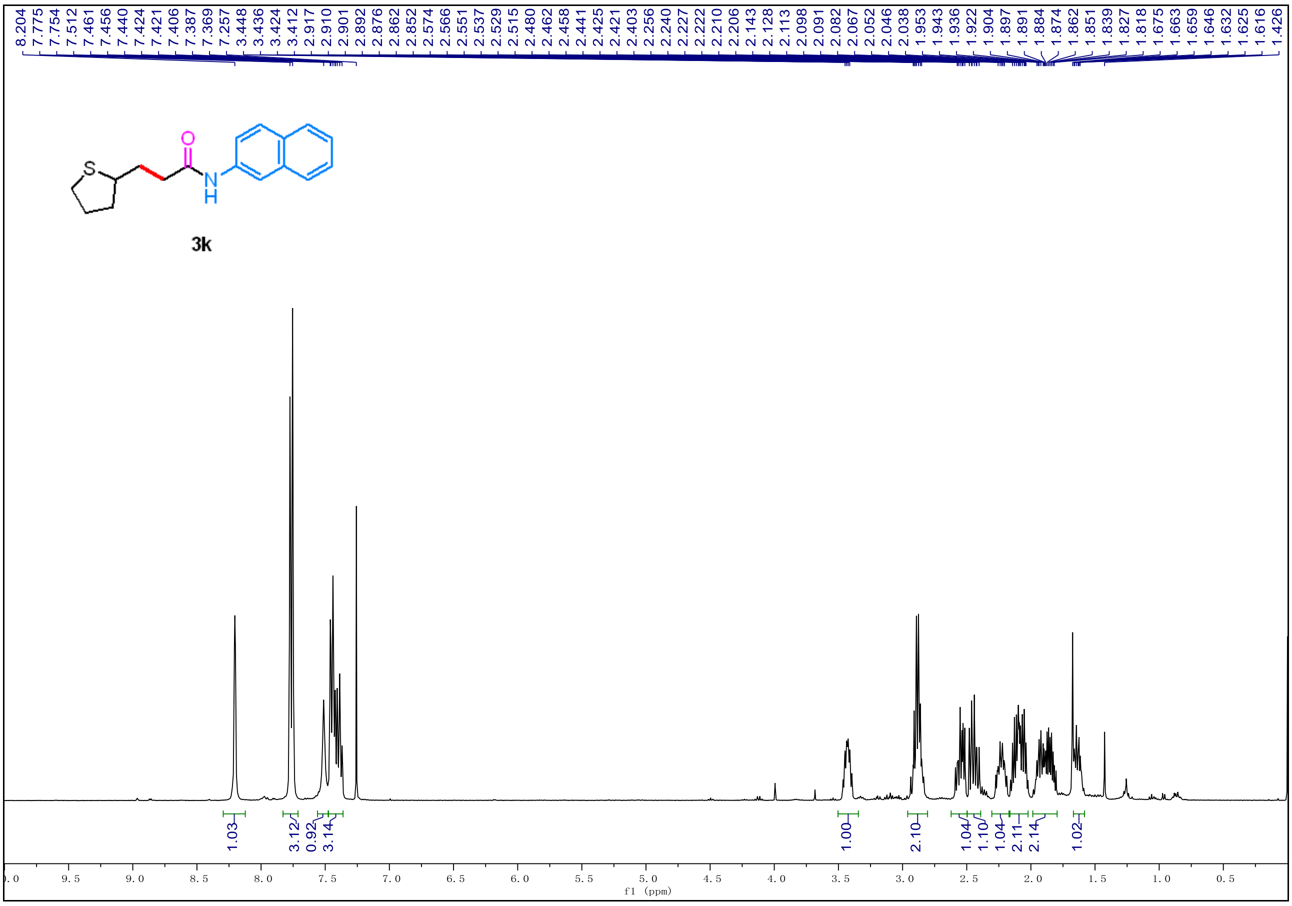

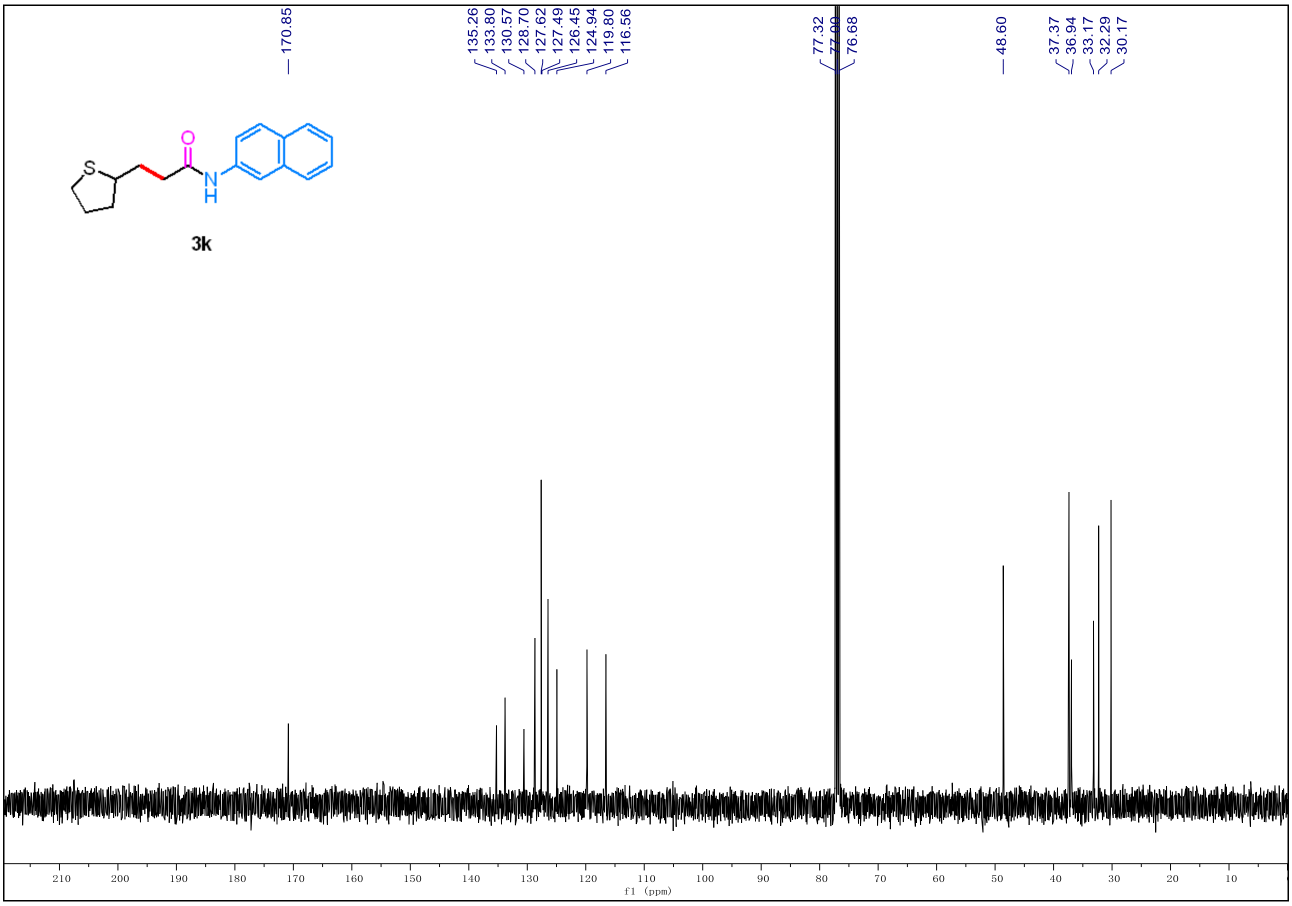

^{1}H NMR (400 MHz, CDCl$_3$) and ^{13}C NMR (100 MHz, CDCl$_3$) spectrum of 3l

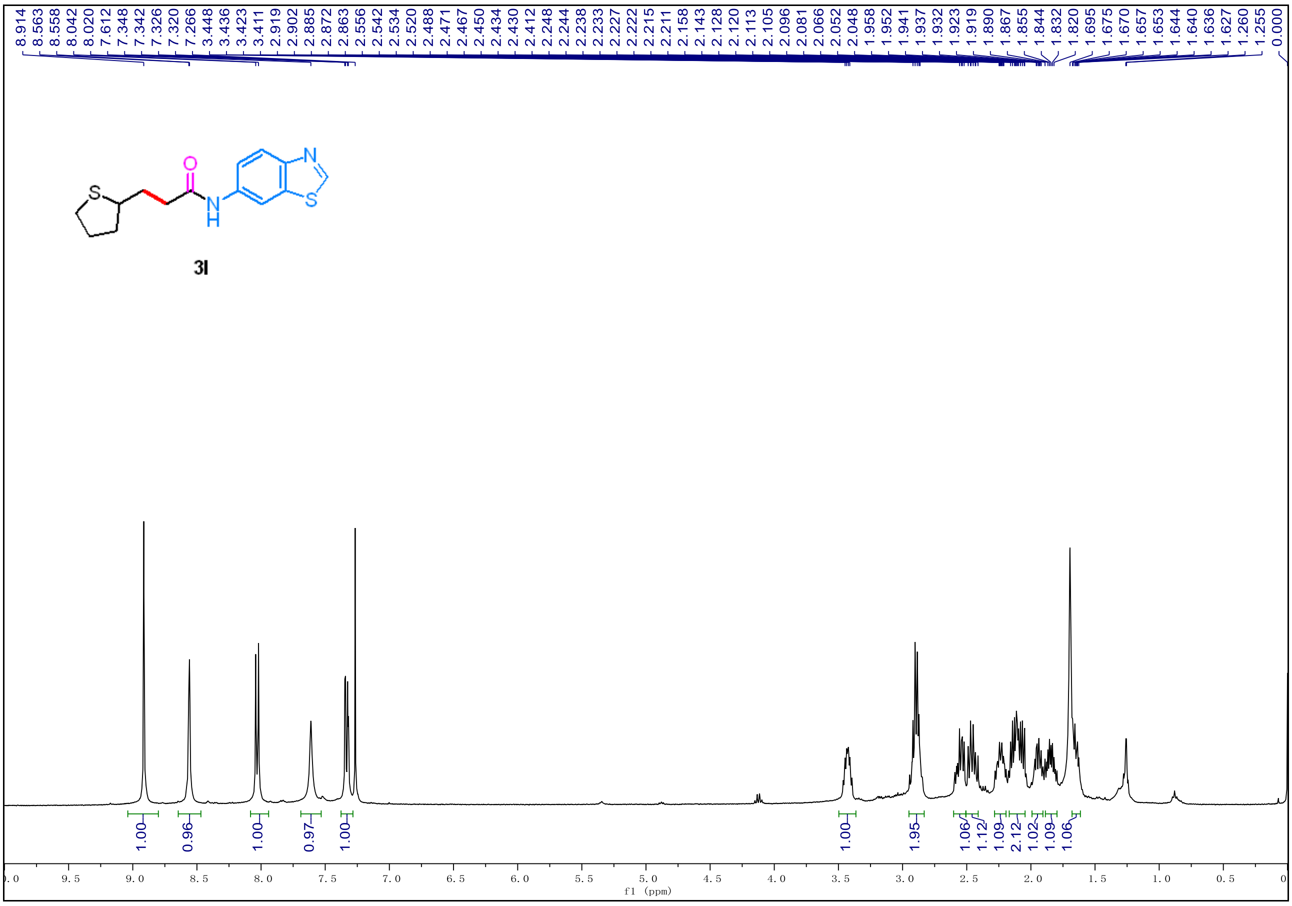

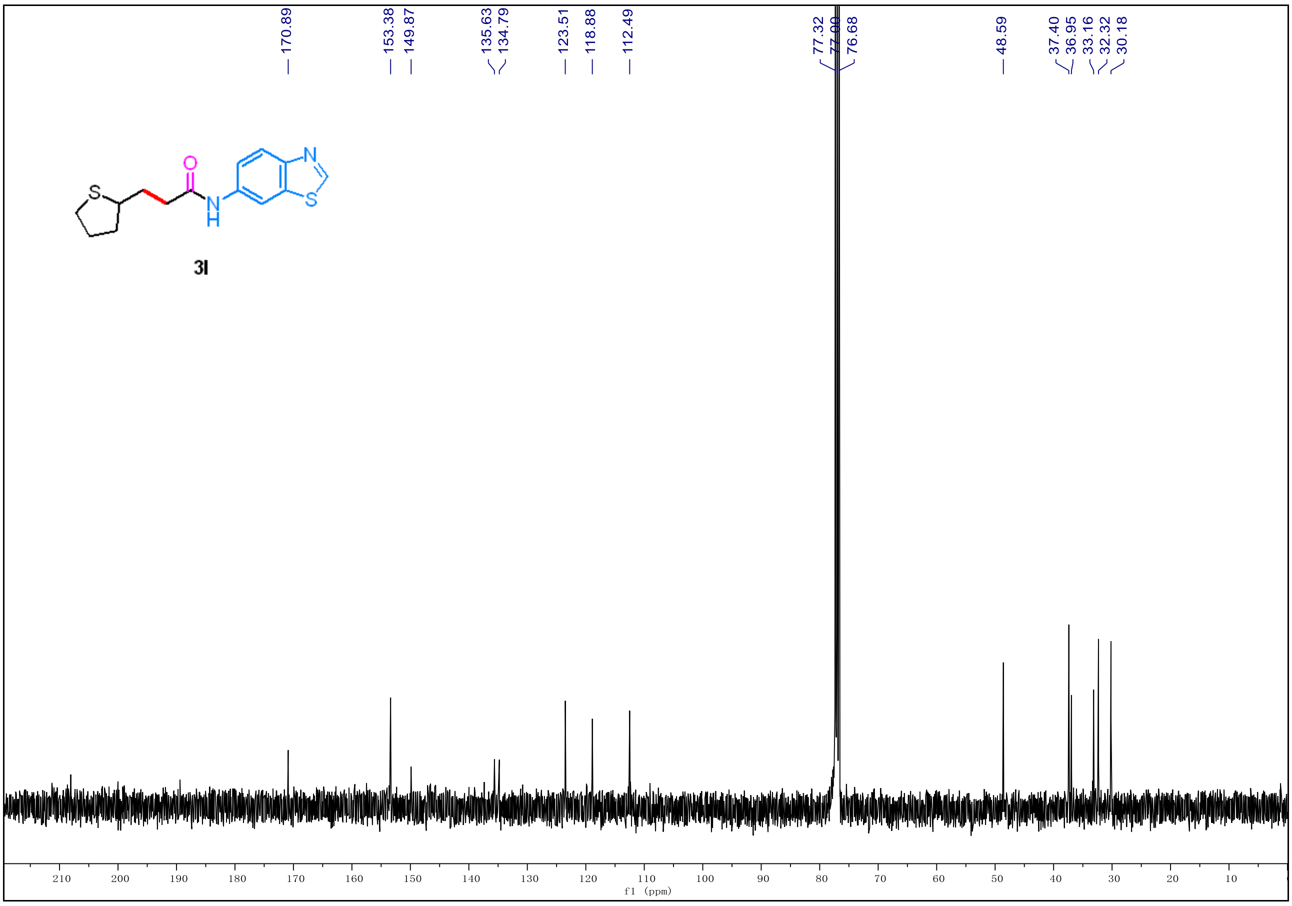

^{1}H NMR (400 MHz, $CDCl_3$) and ^{13}C NMR (100 MHz, $CDCl_3$) spectrum of 3m

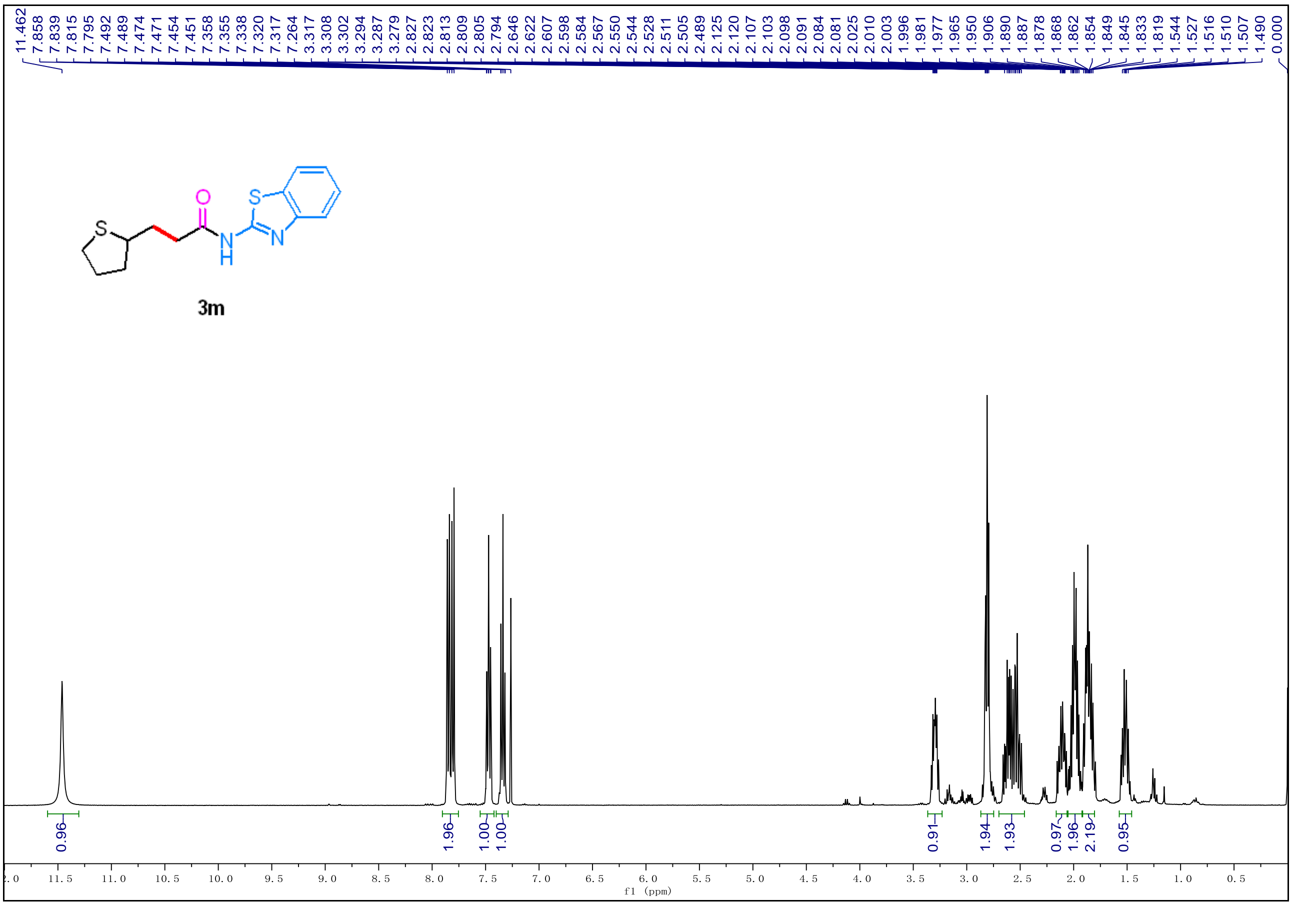

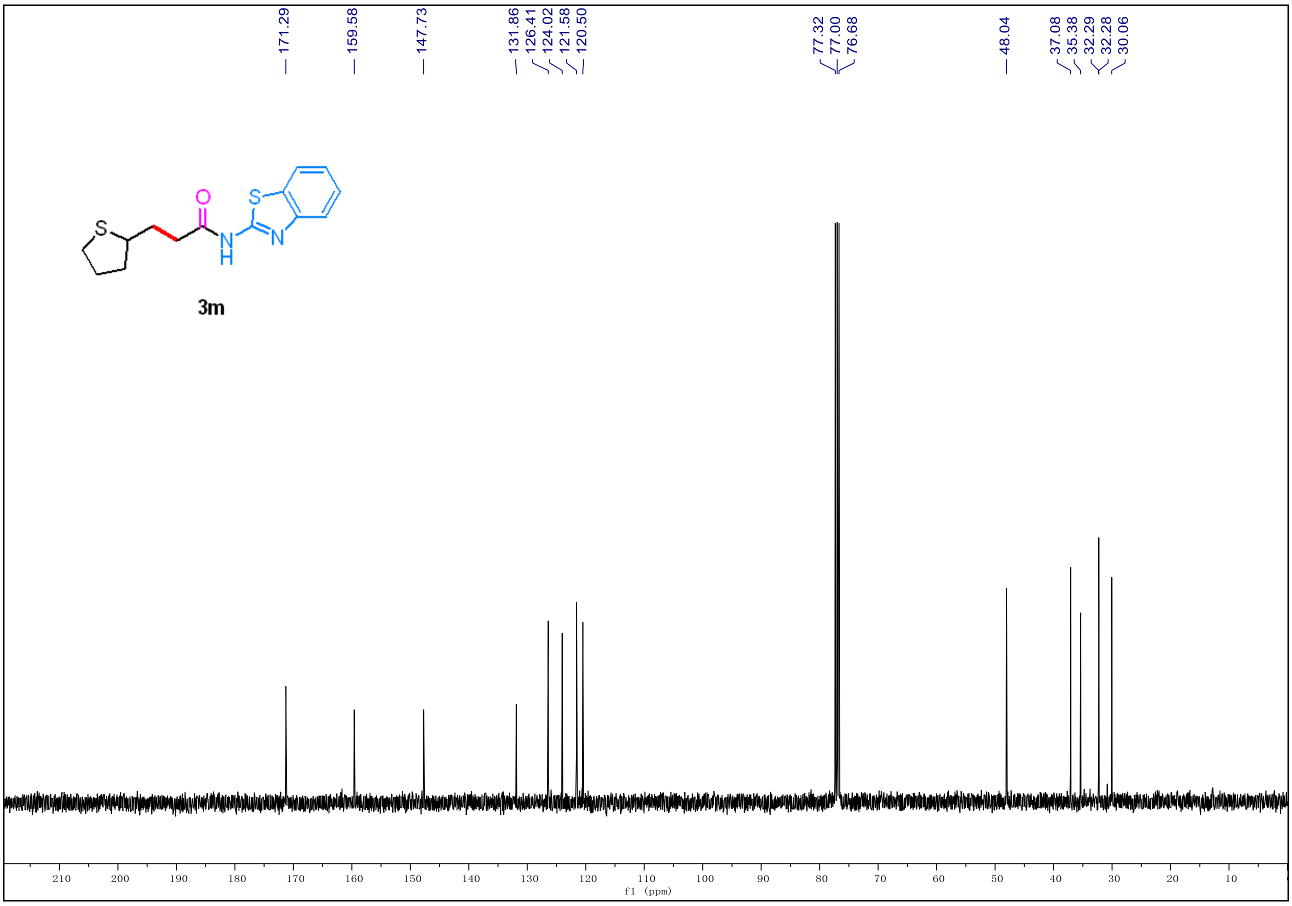

^{1}H NMR (400 MHz, $CDCl_3$) and ^{13}C NMR (100 MHz, $CDCl_3$) spectrum of 3n

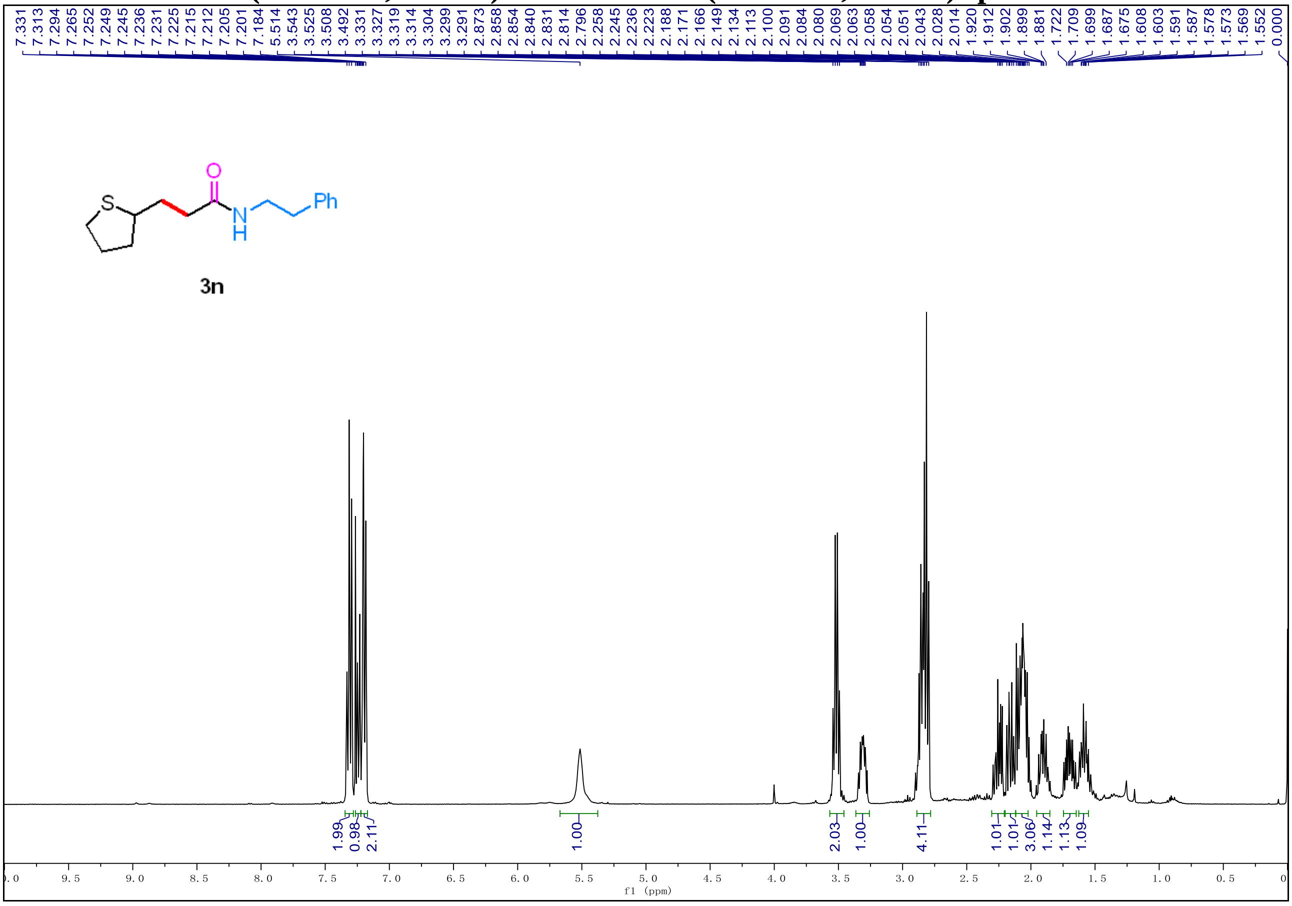

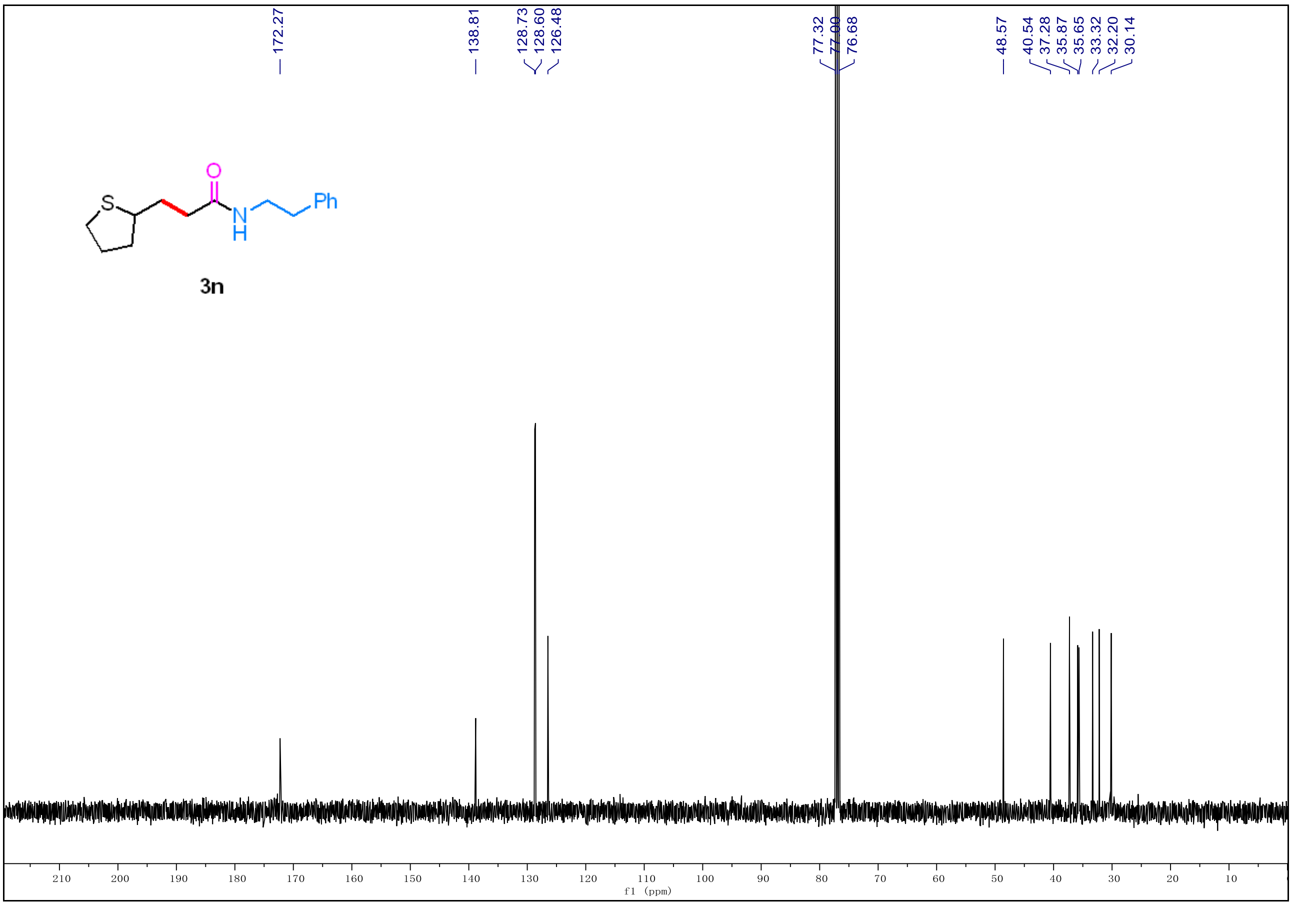

^{1}H NMR (400 MHz, $CDCl_3$) and ^{13}C NMR (100 MHz, $CDCl_3$) spectrum of 3o

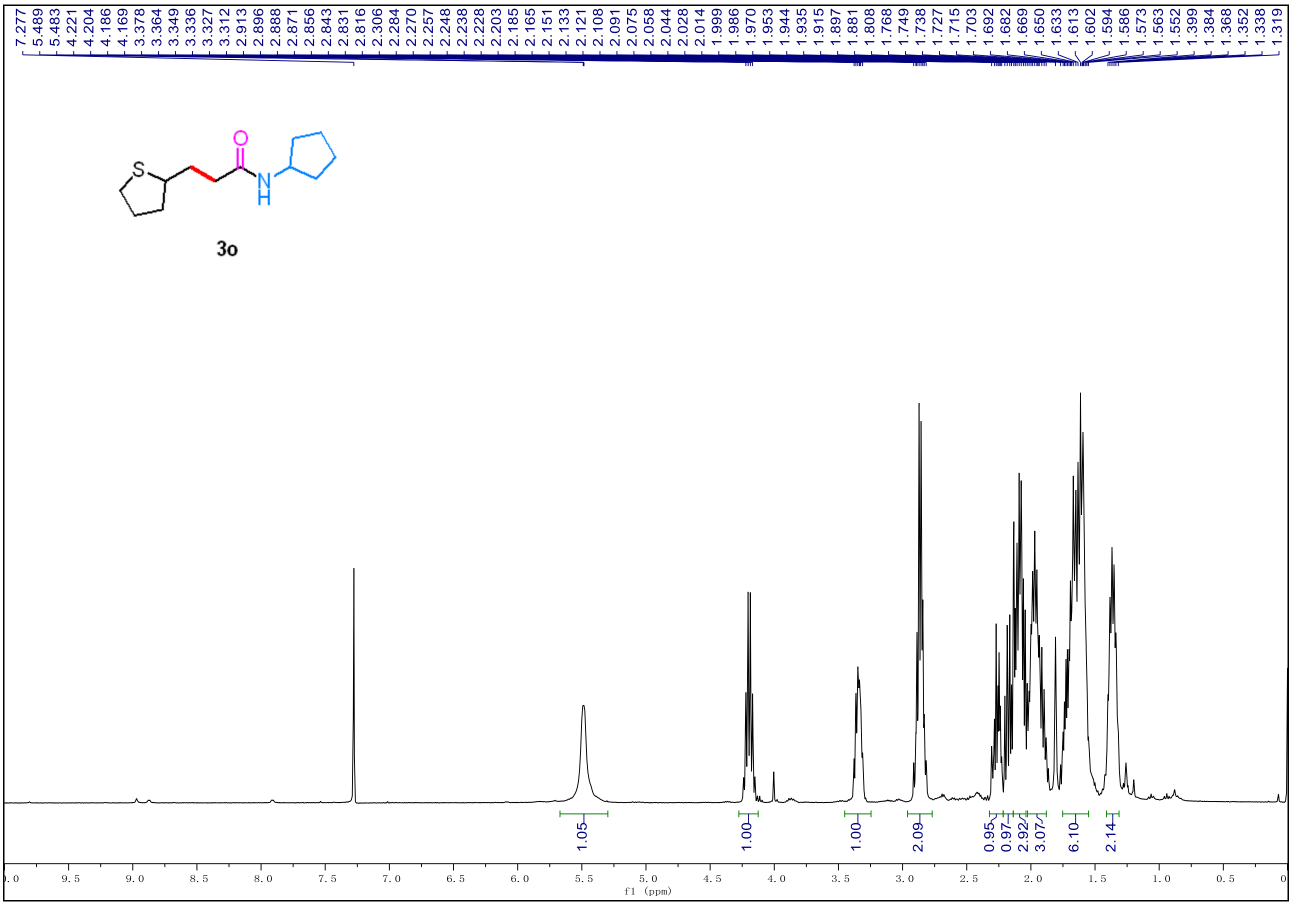

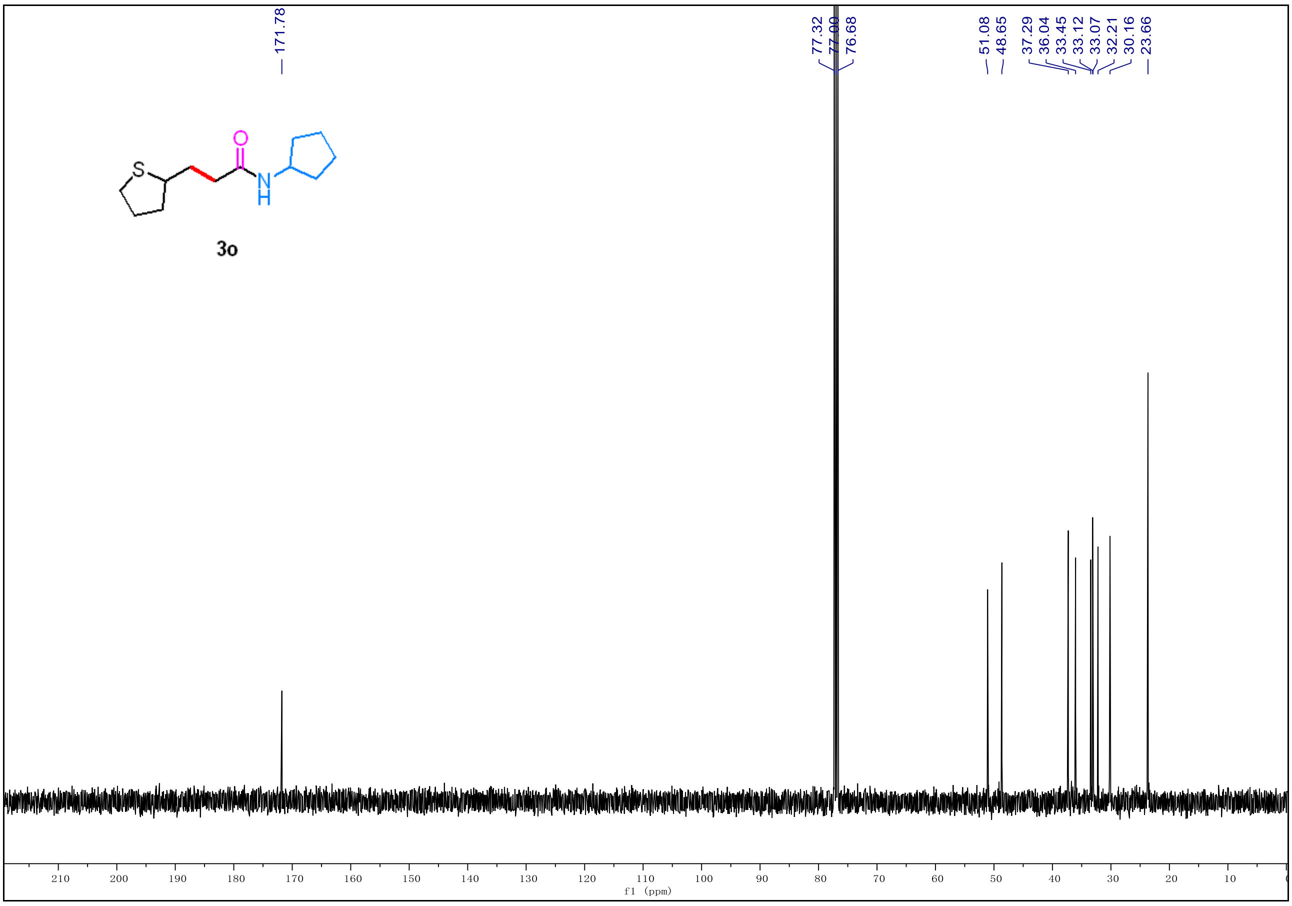

^{1}H NMR (400 MHz, $CDCl_3$) and ^{13}C NMR (100 MHz, $CDCl_3$) spectrum of 3p

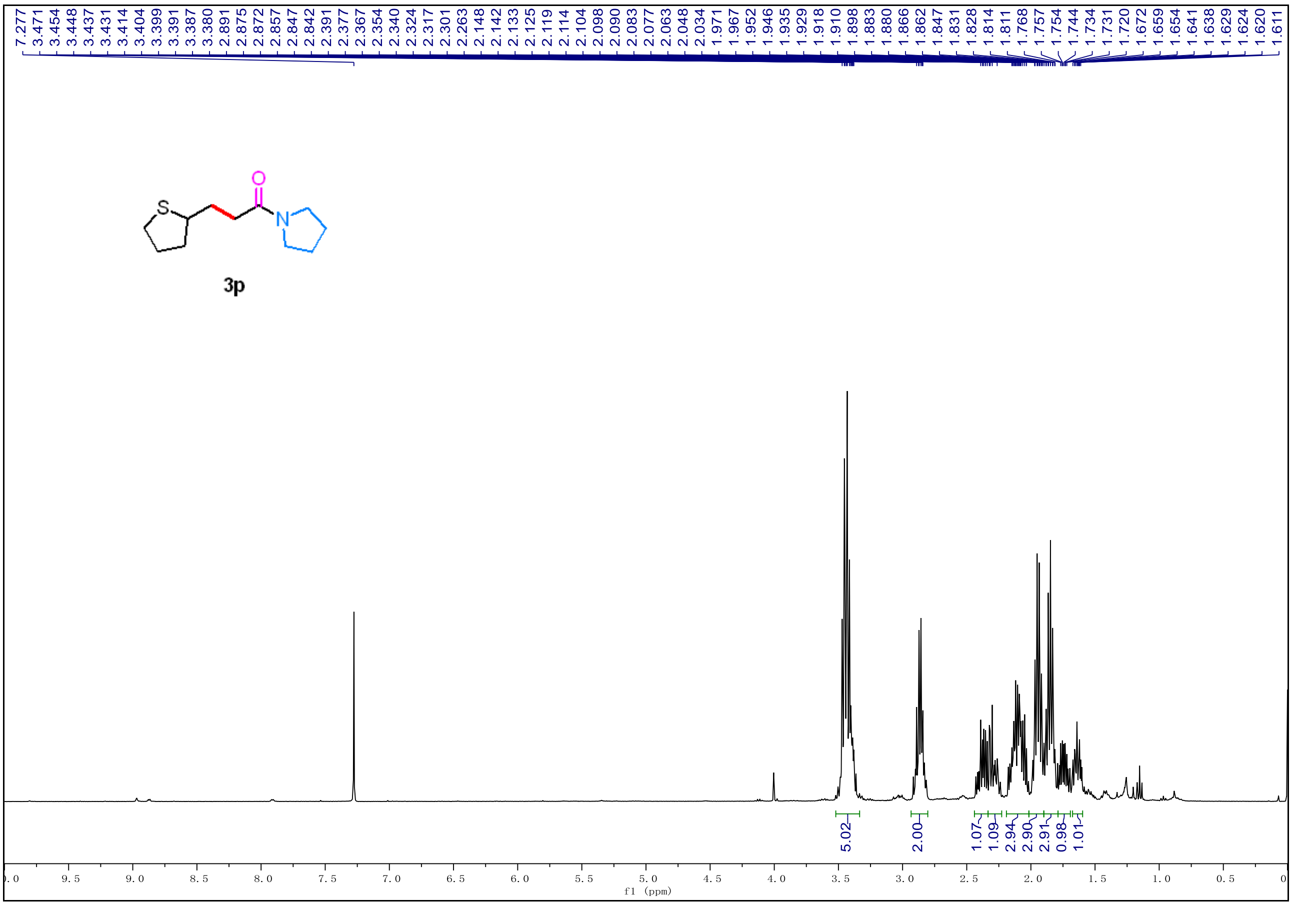

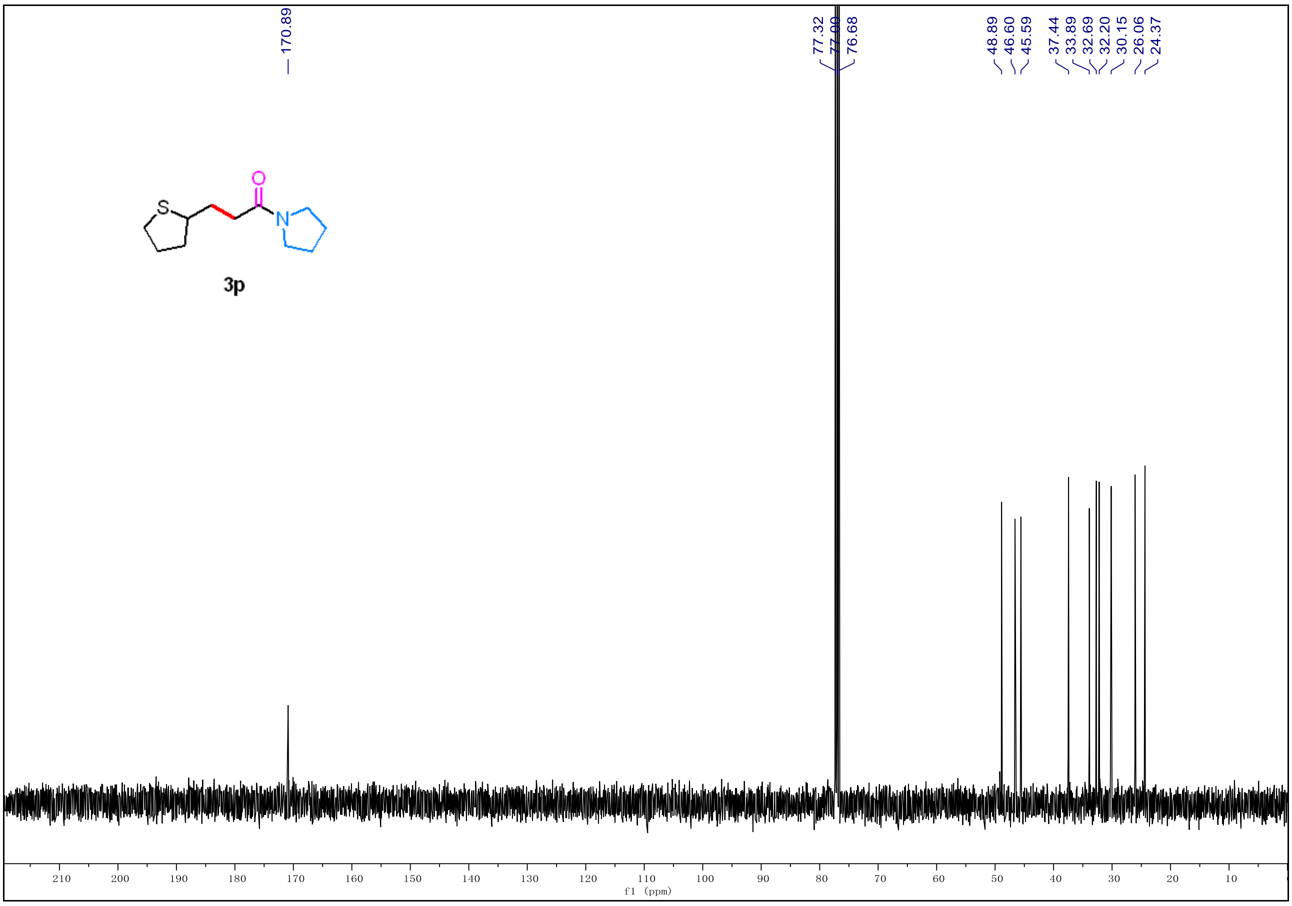

^{1}H NMR (400 MHz, $CDCl_3$) and ^{13}C NMR (100 MHz, $CDCl_3$) spectrum of 3q

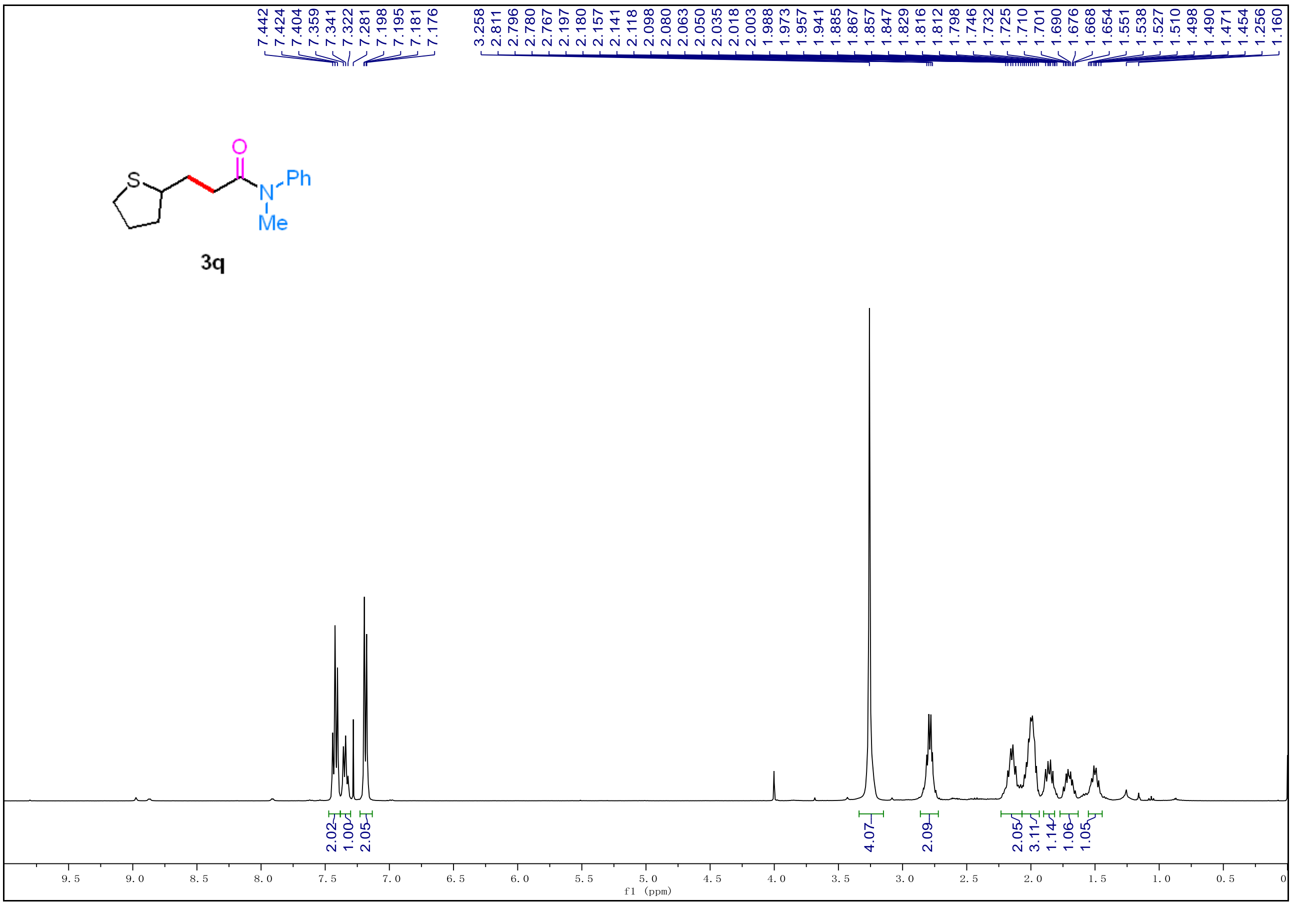

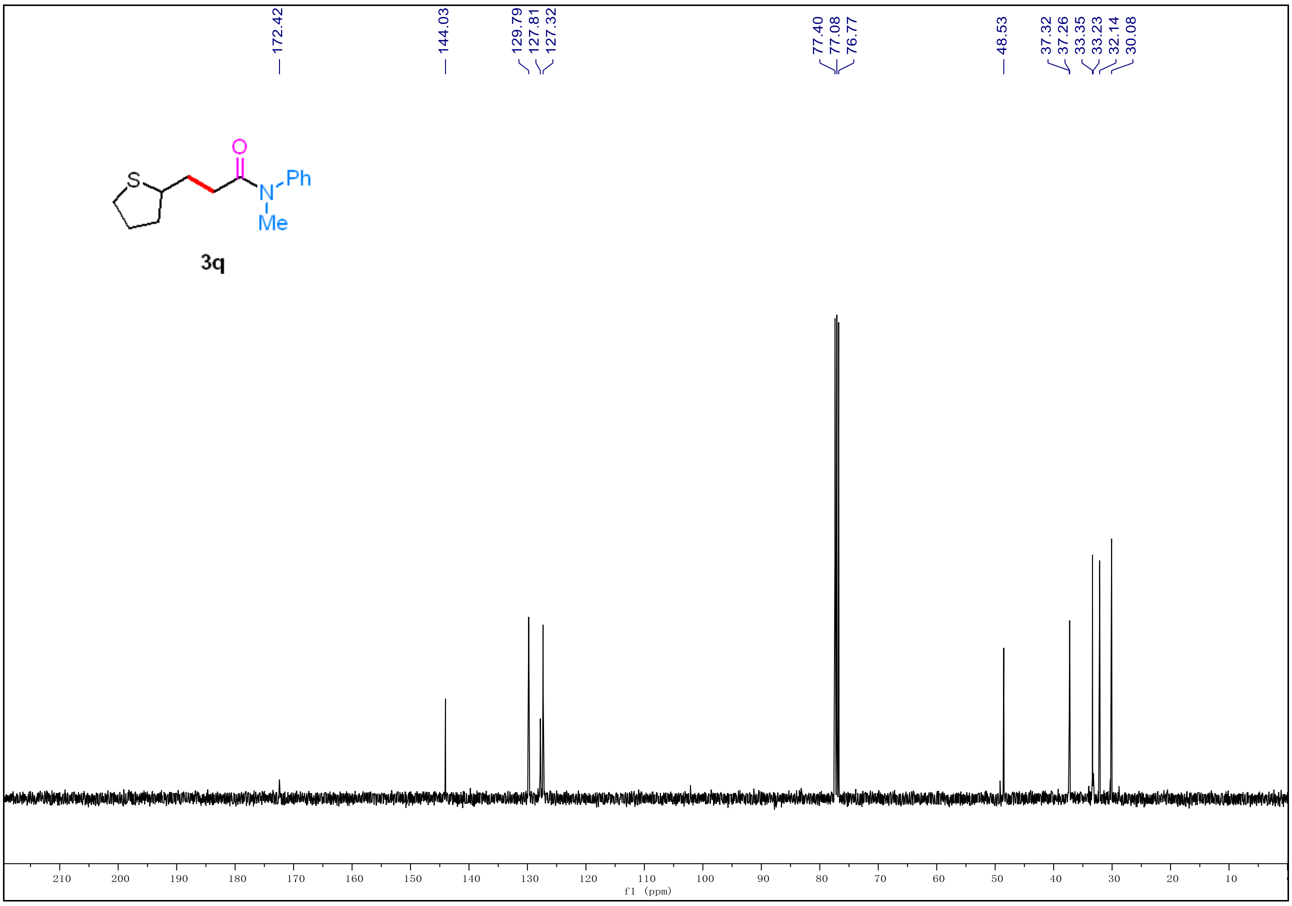

^{1}H NMR (400 MHz, CDCl3) and ^{13}C NMR (100 MHz, CDCl3) spectrum of 3r

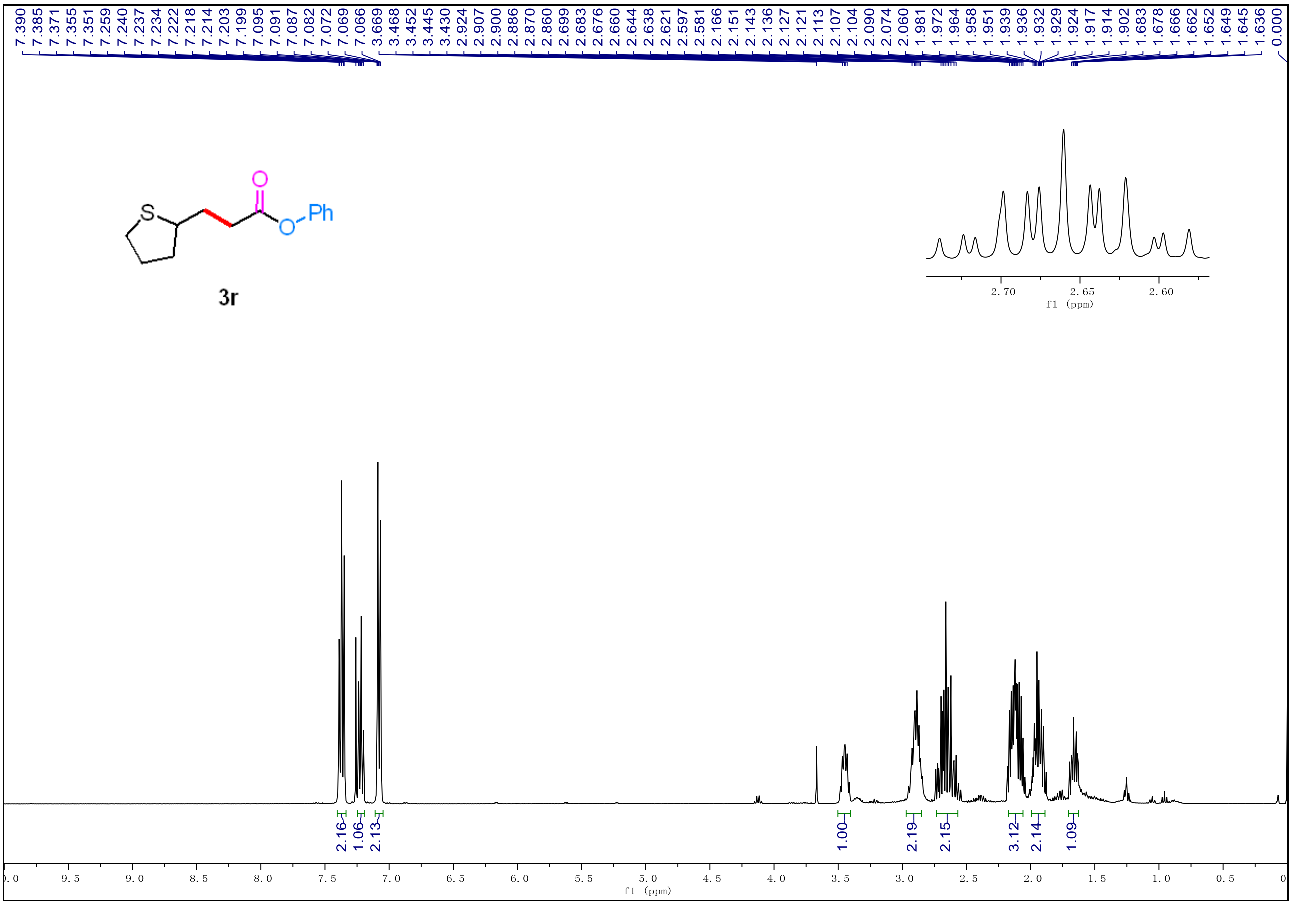

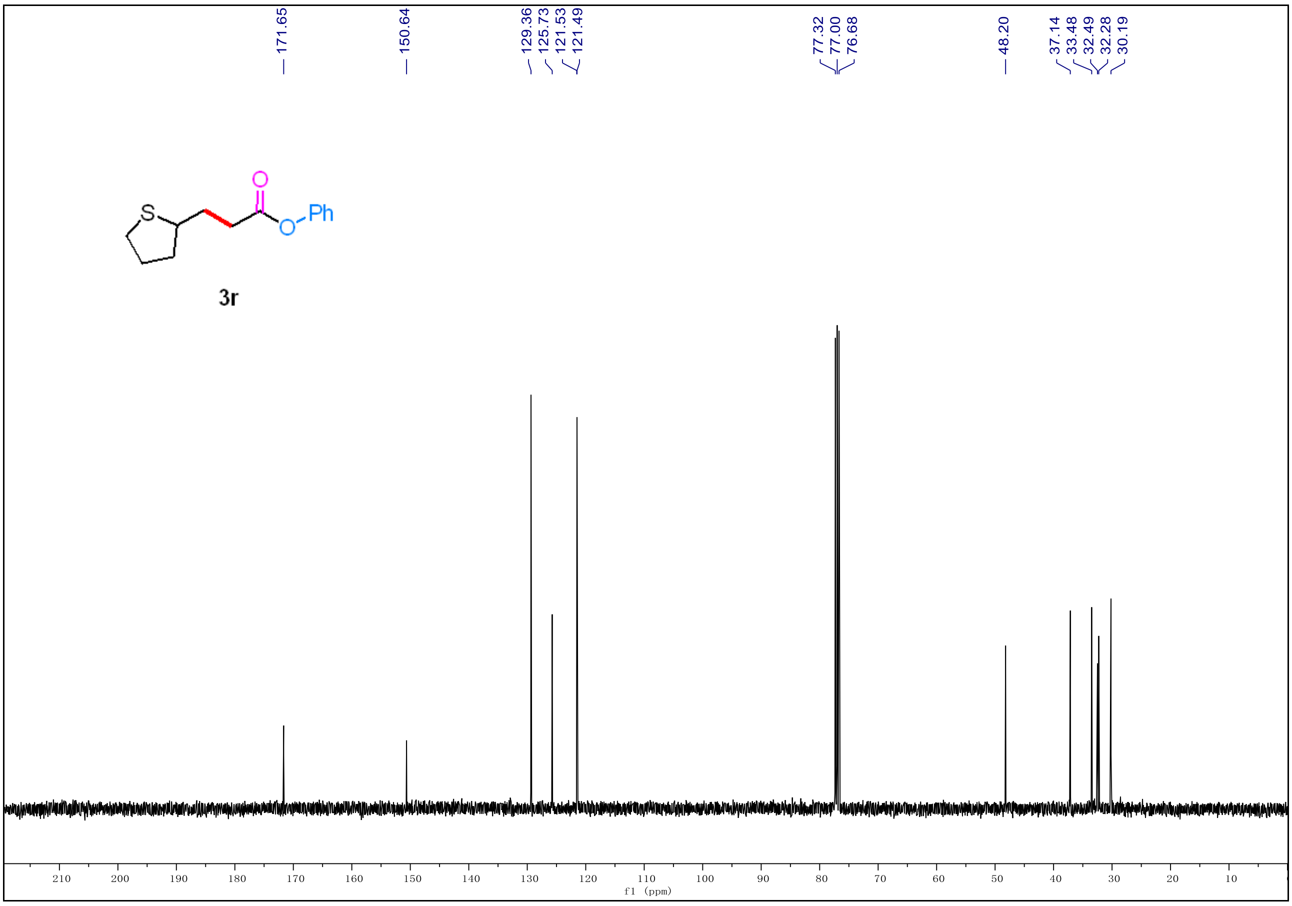

^{1}H NMR (400 MHz, $CDCl_3$) and ^{13}C NMR (100 MHz, $CDCl_3$) spectrum of 3s

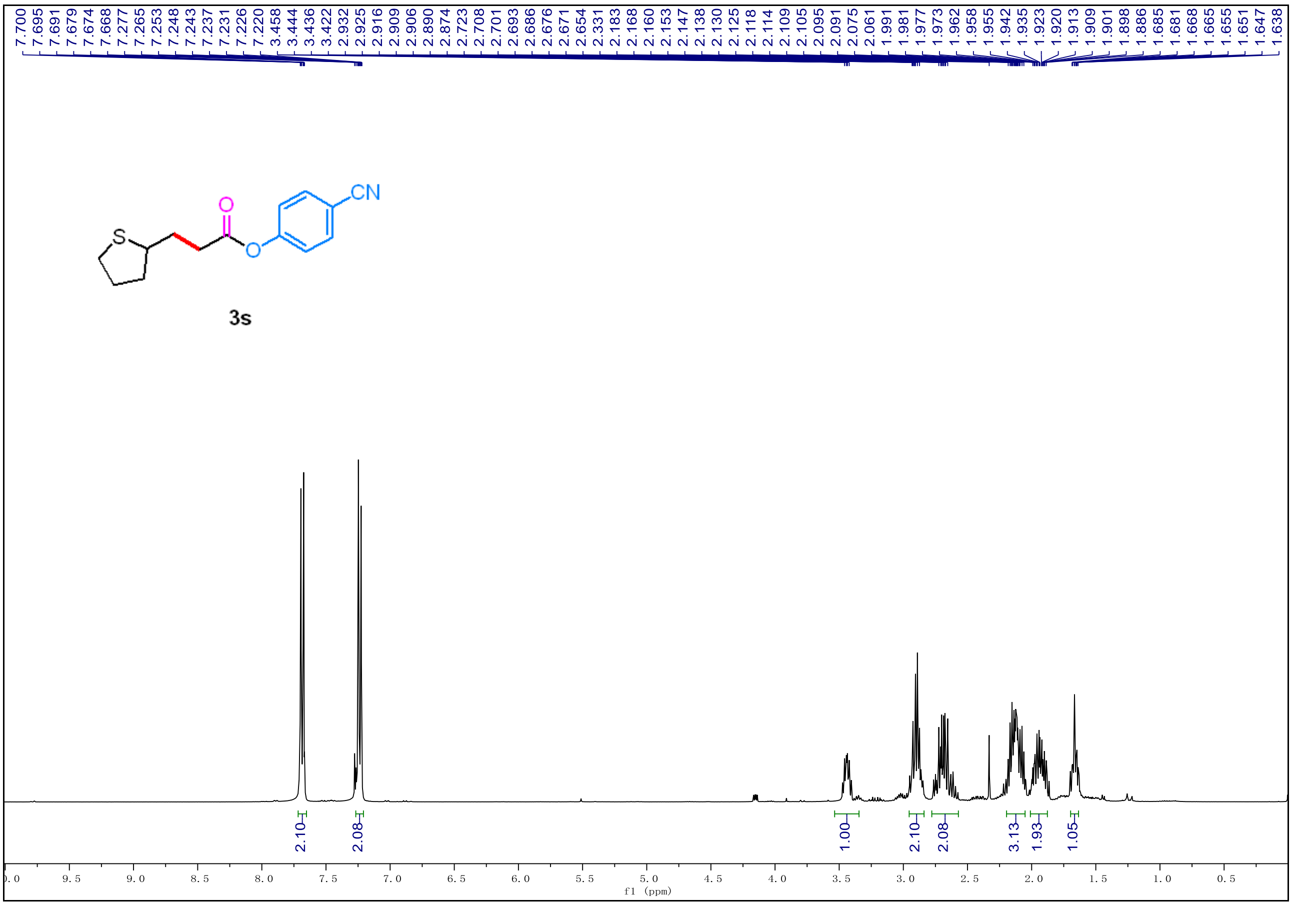

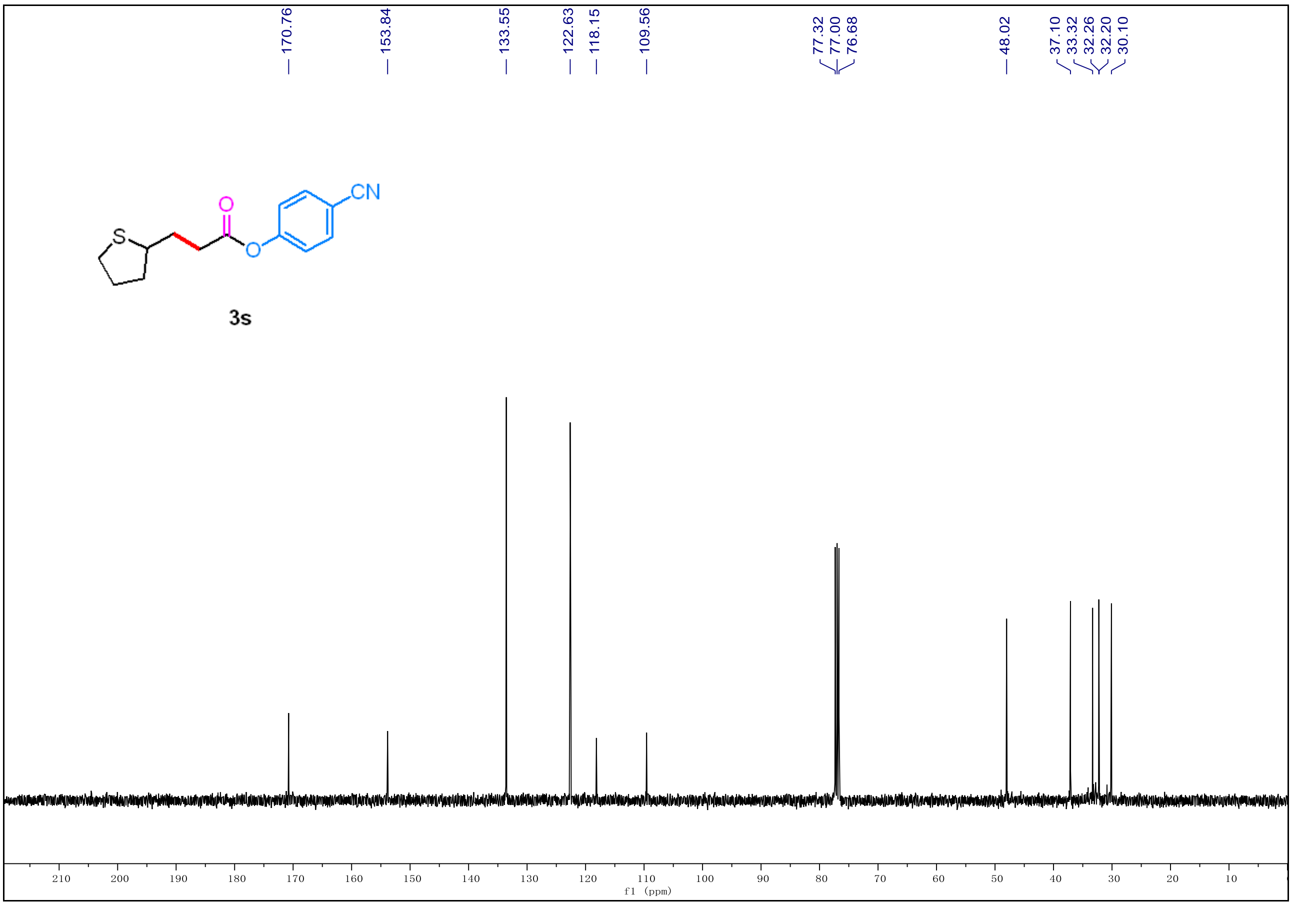

^{1}H NMR (400 MHz, CDCl$_3$) and ^{13}C NMR (100 MHz, CDCl$_3$) spectrum of 3t

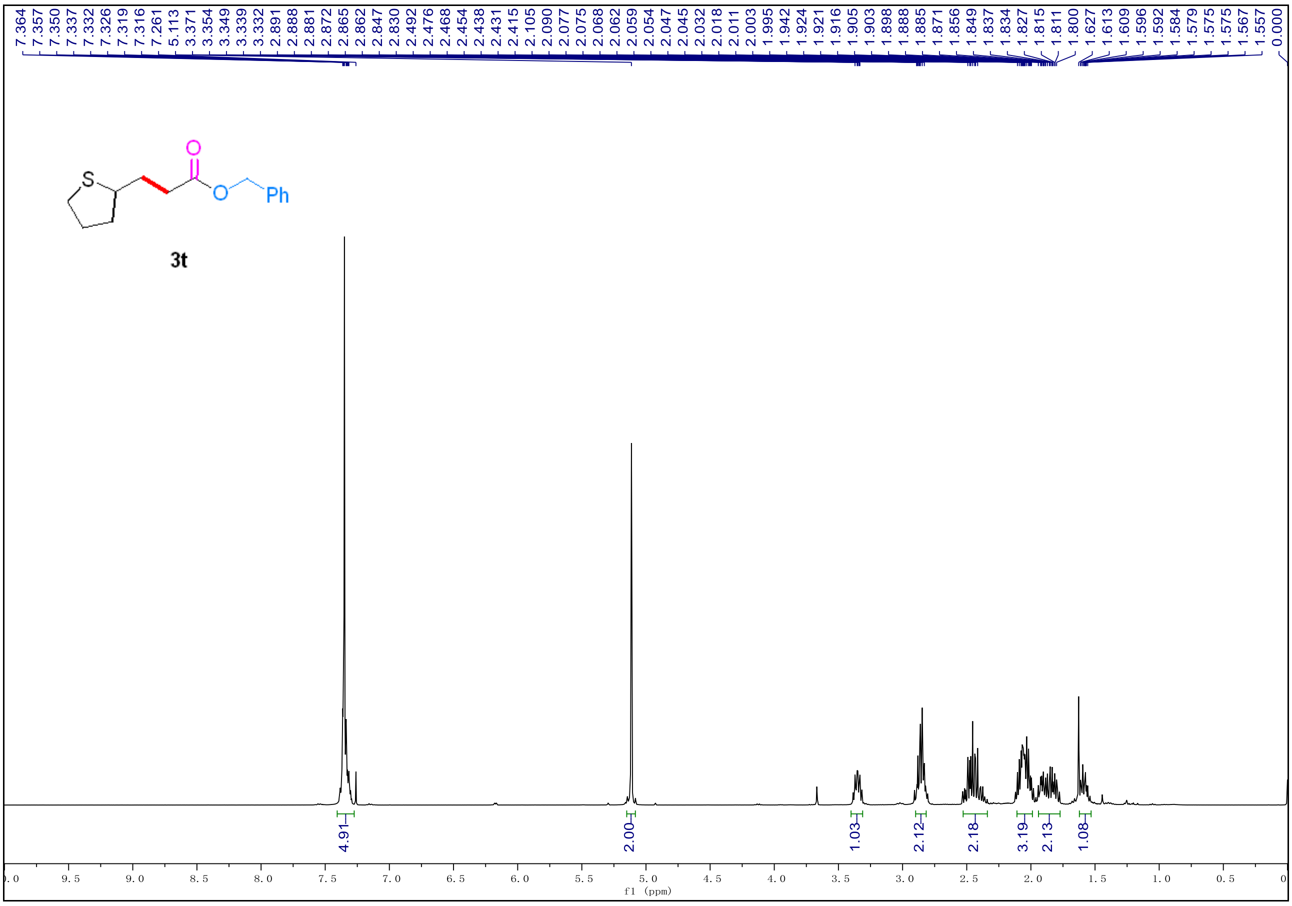

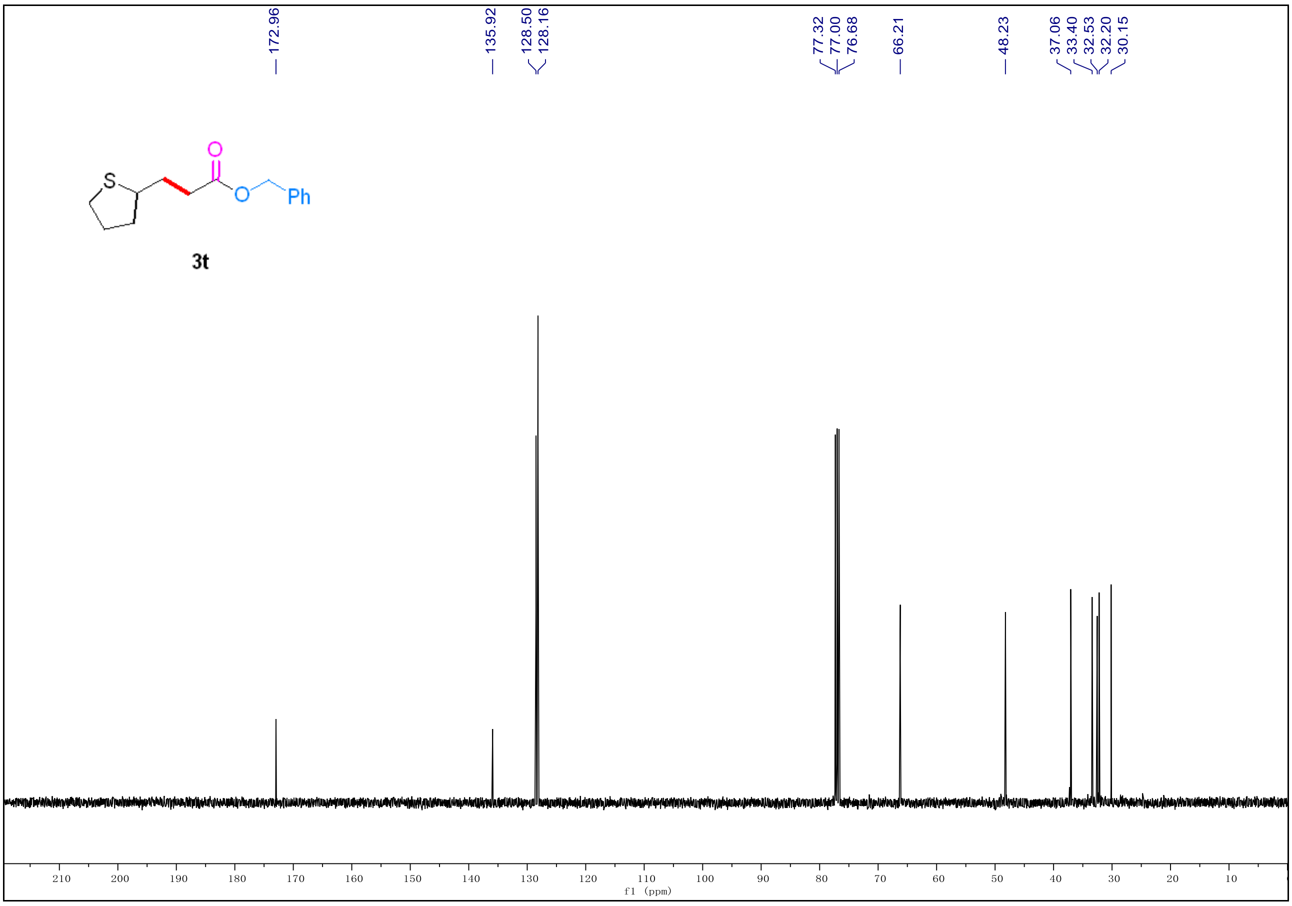

^{1}H NMR (400 MHz, $CDCl_3$) and ^{13}C NMR (100 MHz, $CDCl_3$) spectrum of 3u

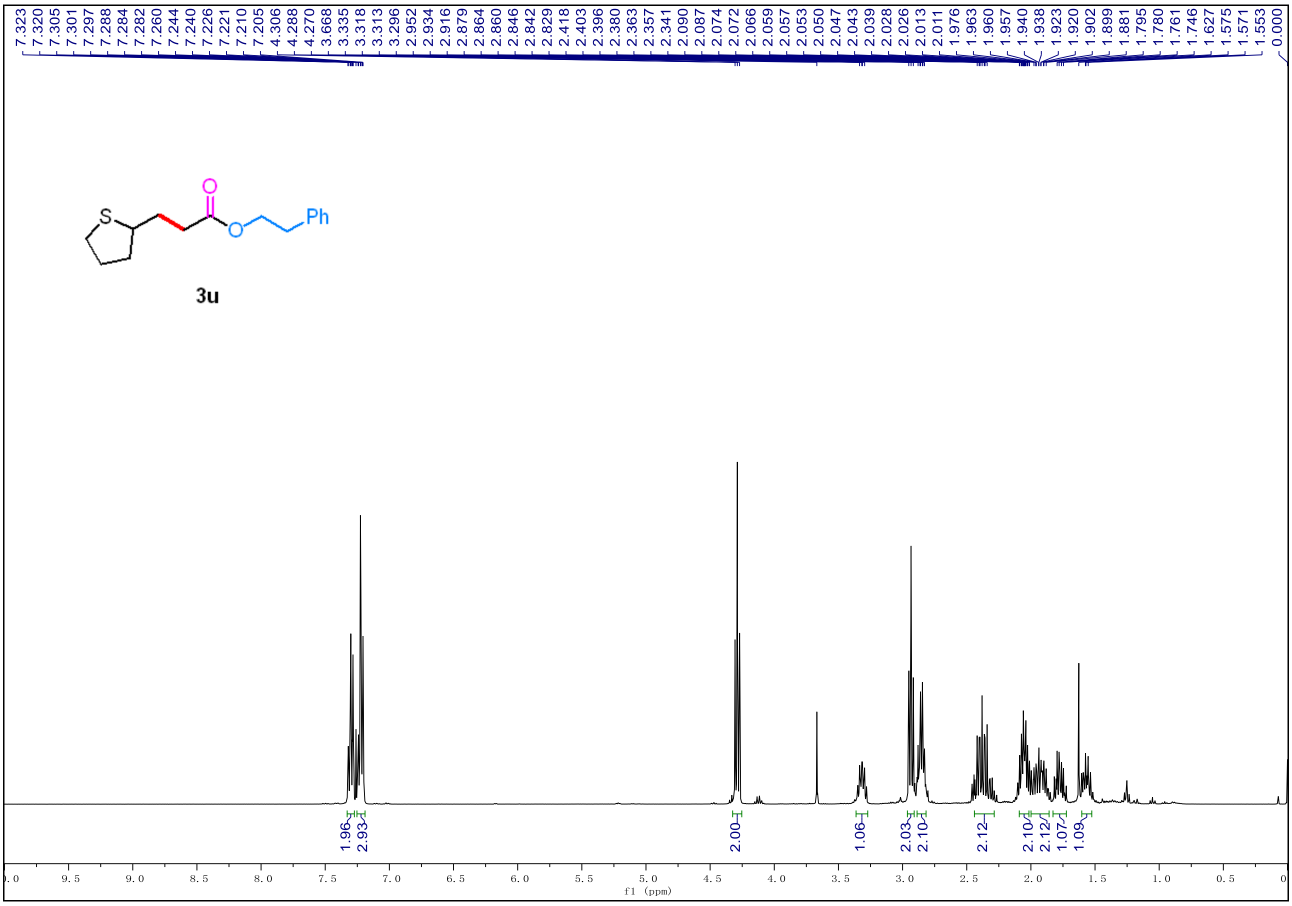

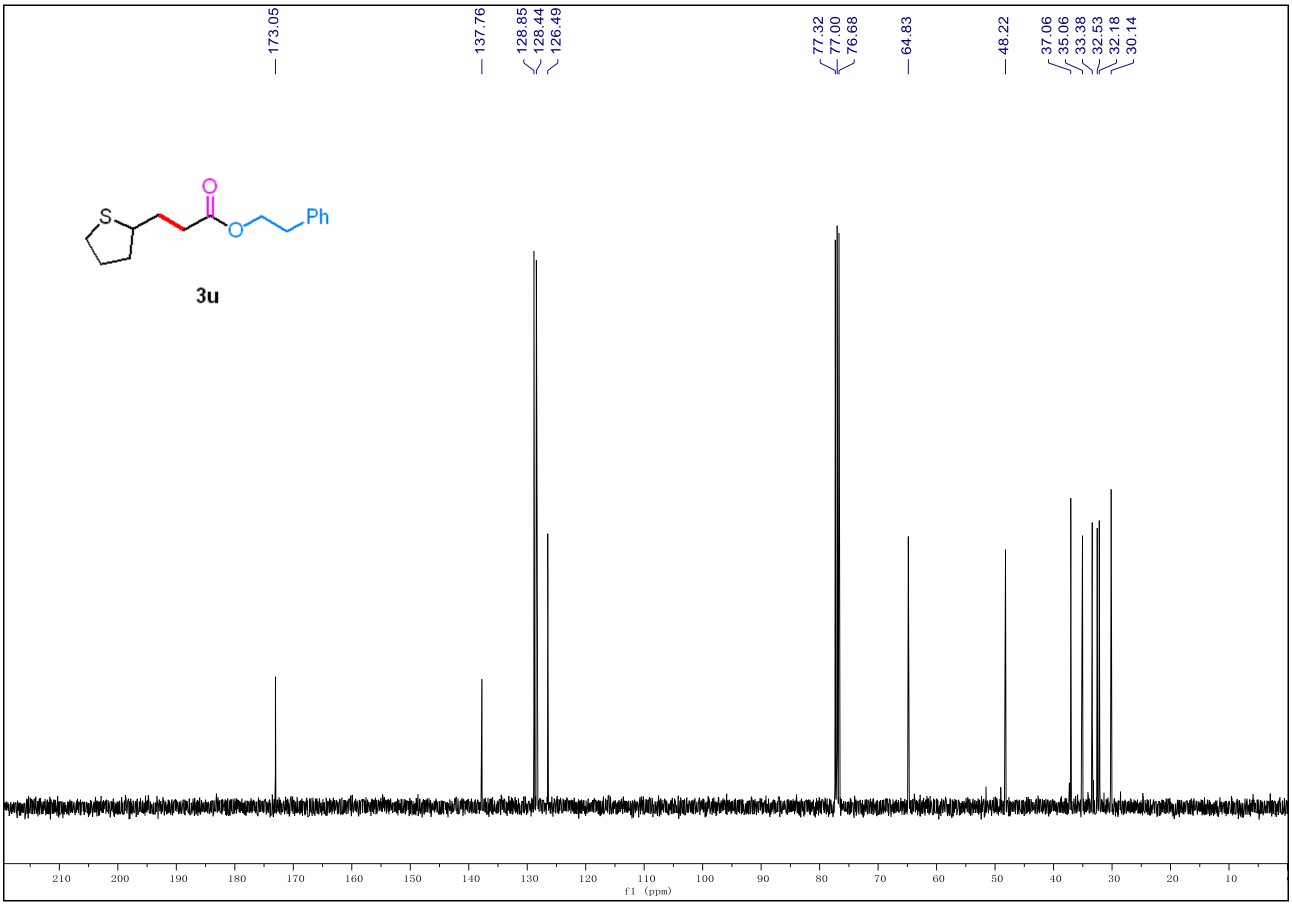

^{1}H NMR (400 MHz, $CDCl_3$) and ^{13}C NMR (100 MHz, $CDCl_3$) spectrum of 3v

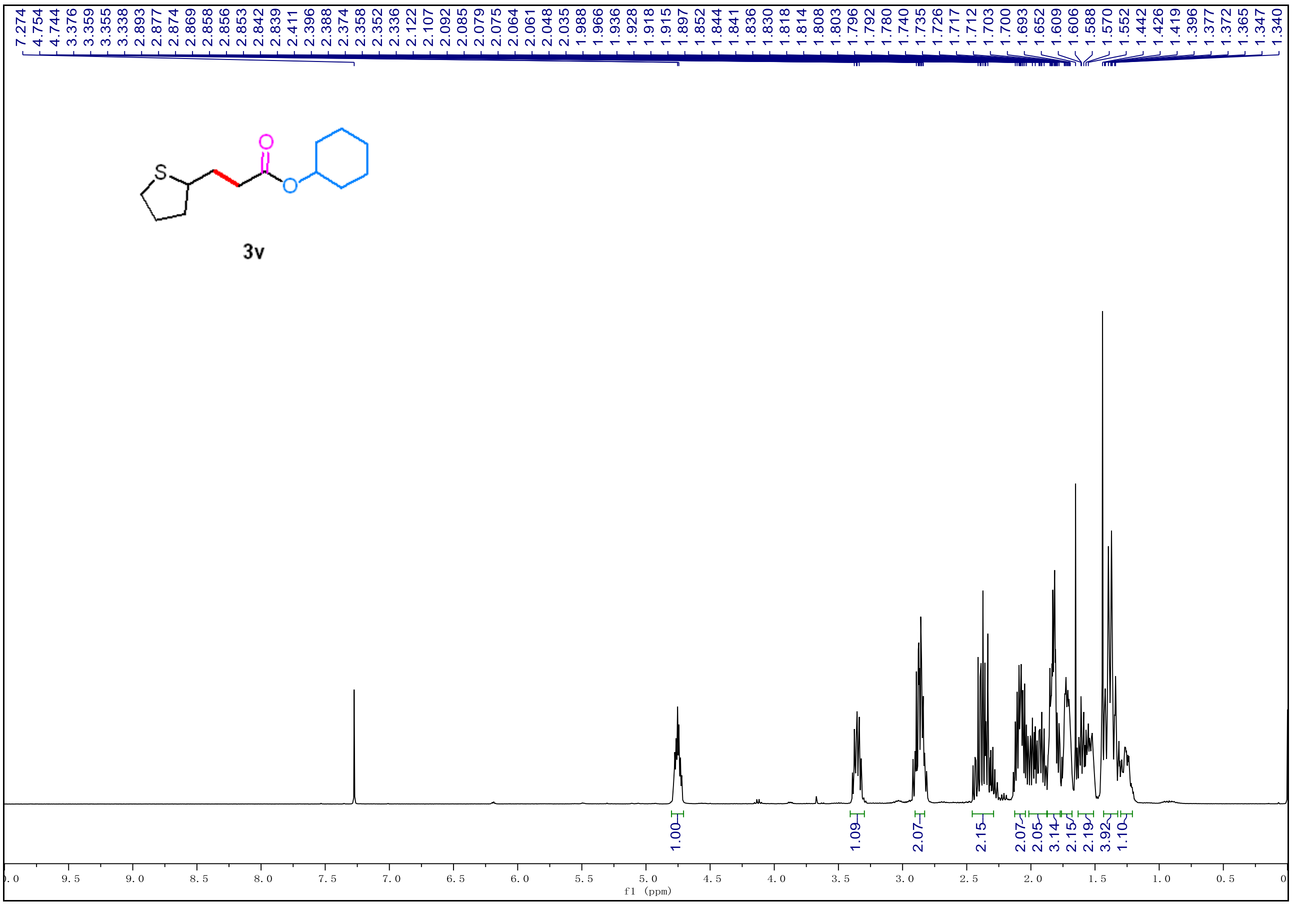

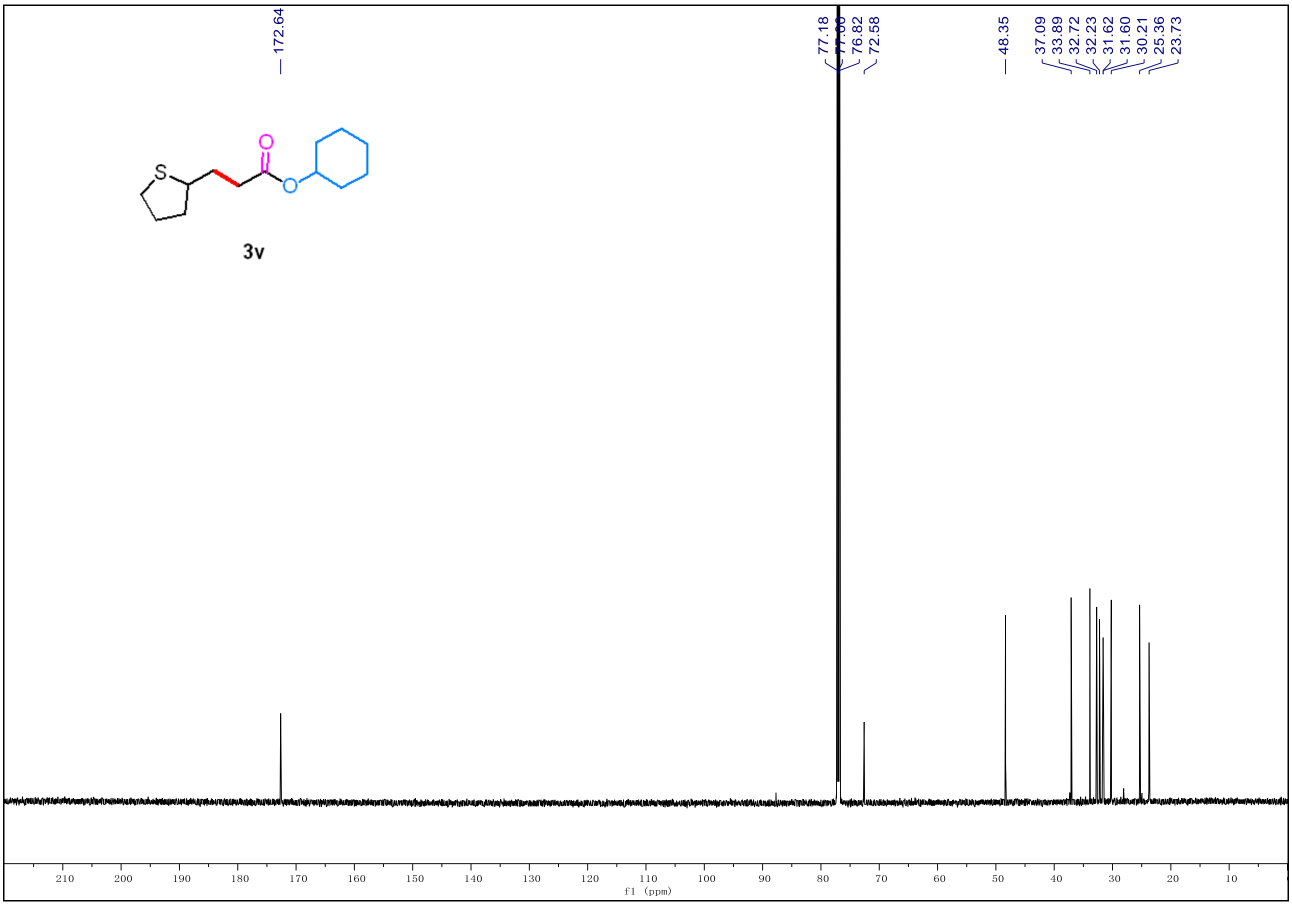

^{1}H NMR (400 MHz, $CDCl_3$) and ^{13}C NMR (100 MHz, $CDCl_3$) spectrum of 3w

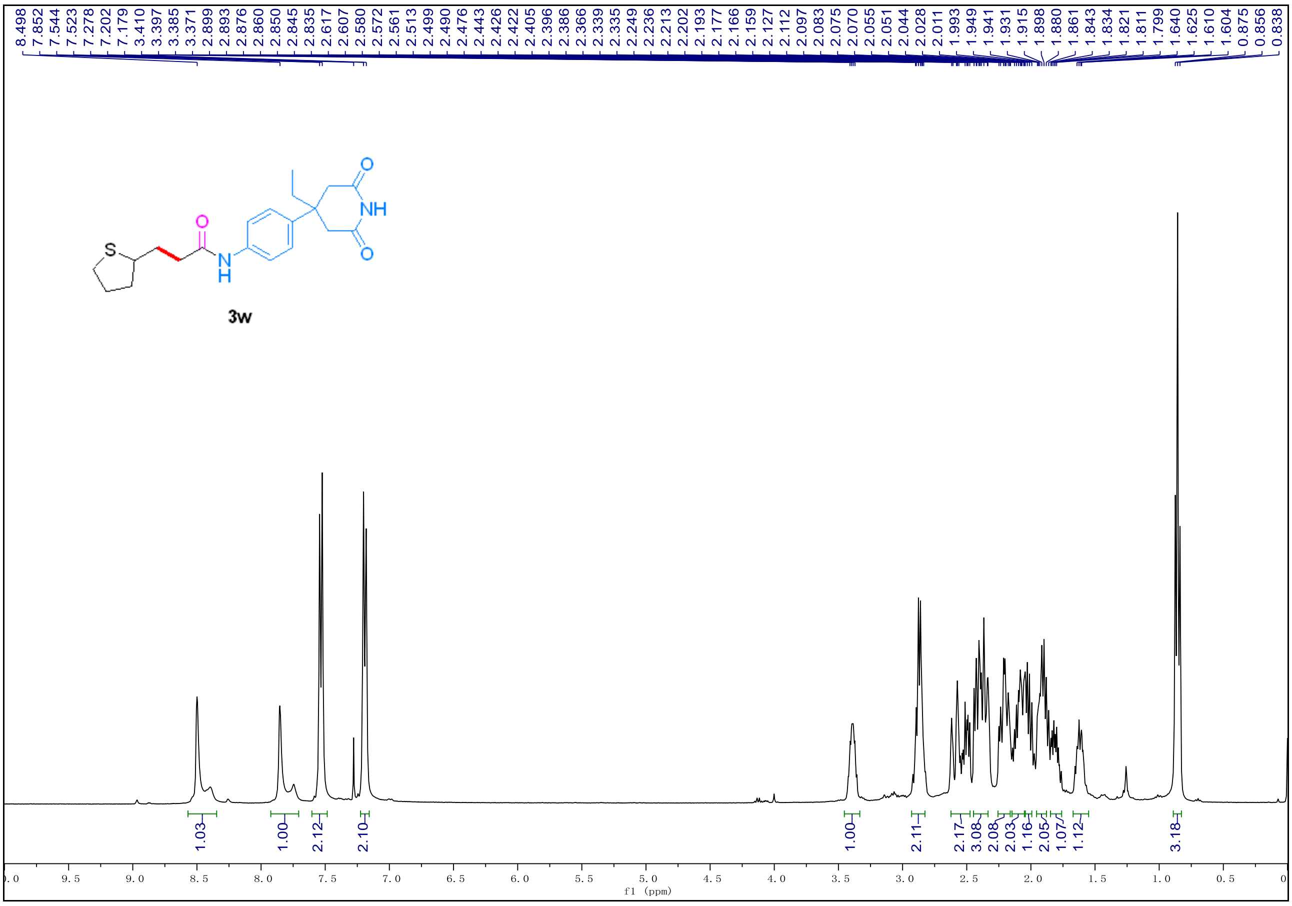

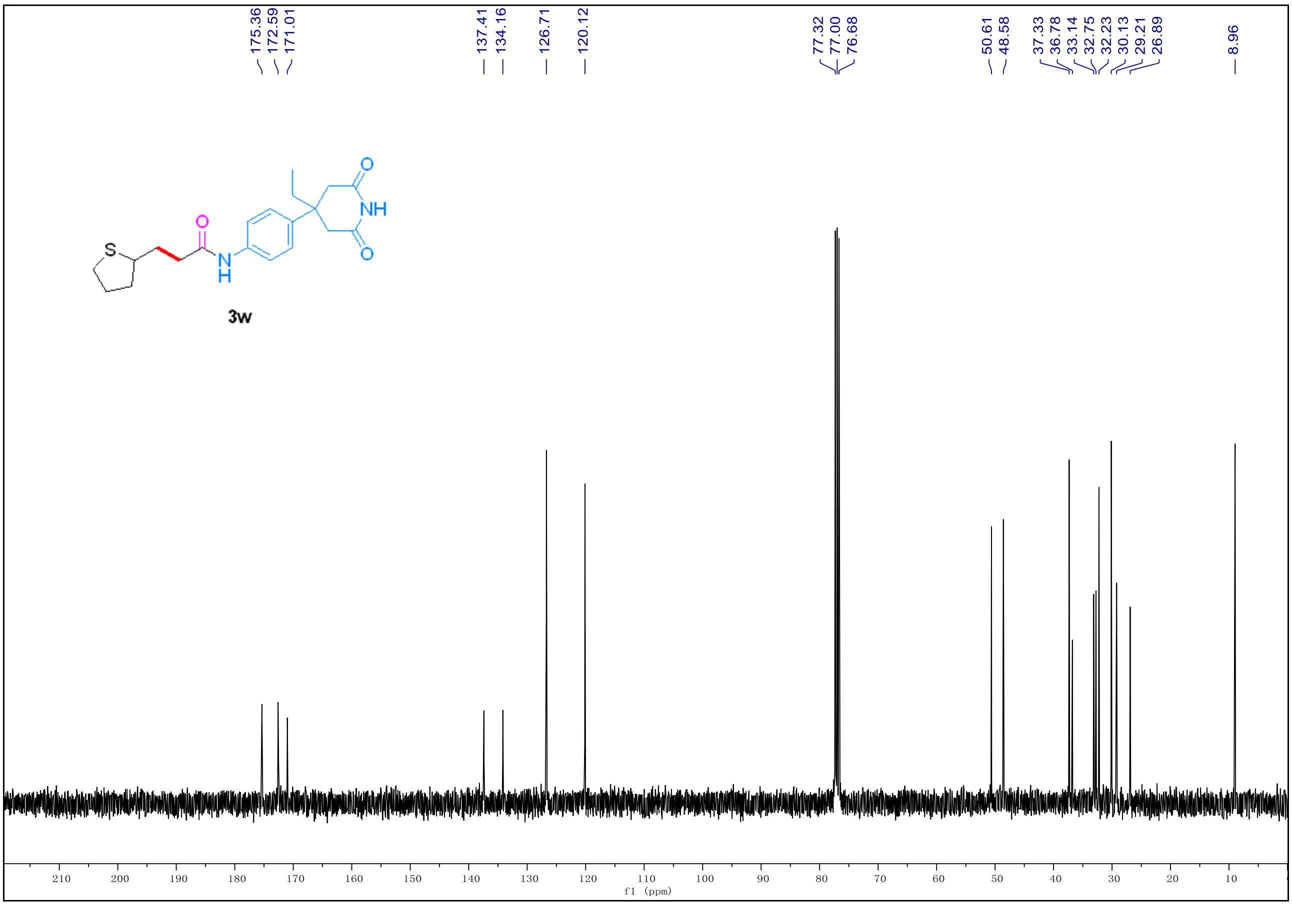

^{1}H NMR (400 MHz, CDCl3) and ^{13}C NMR (100 MHz, CDCl3) spectrum of 3x

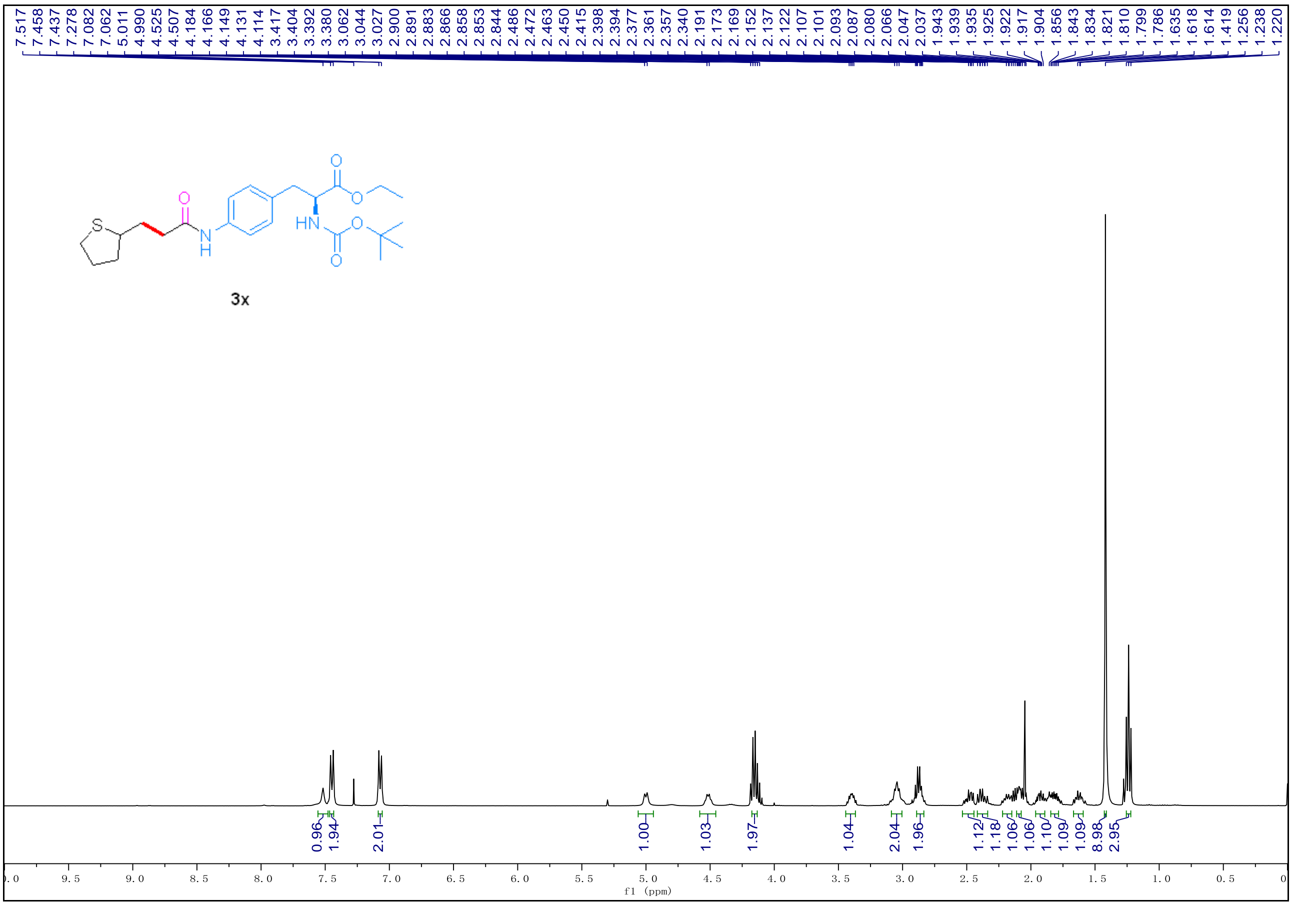

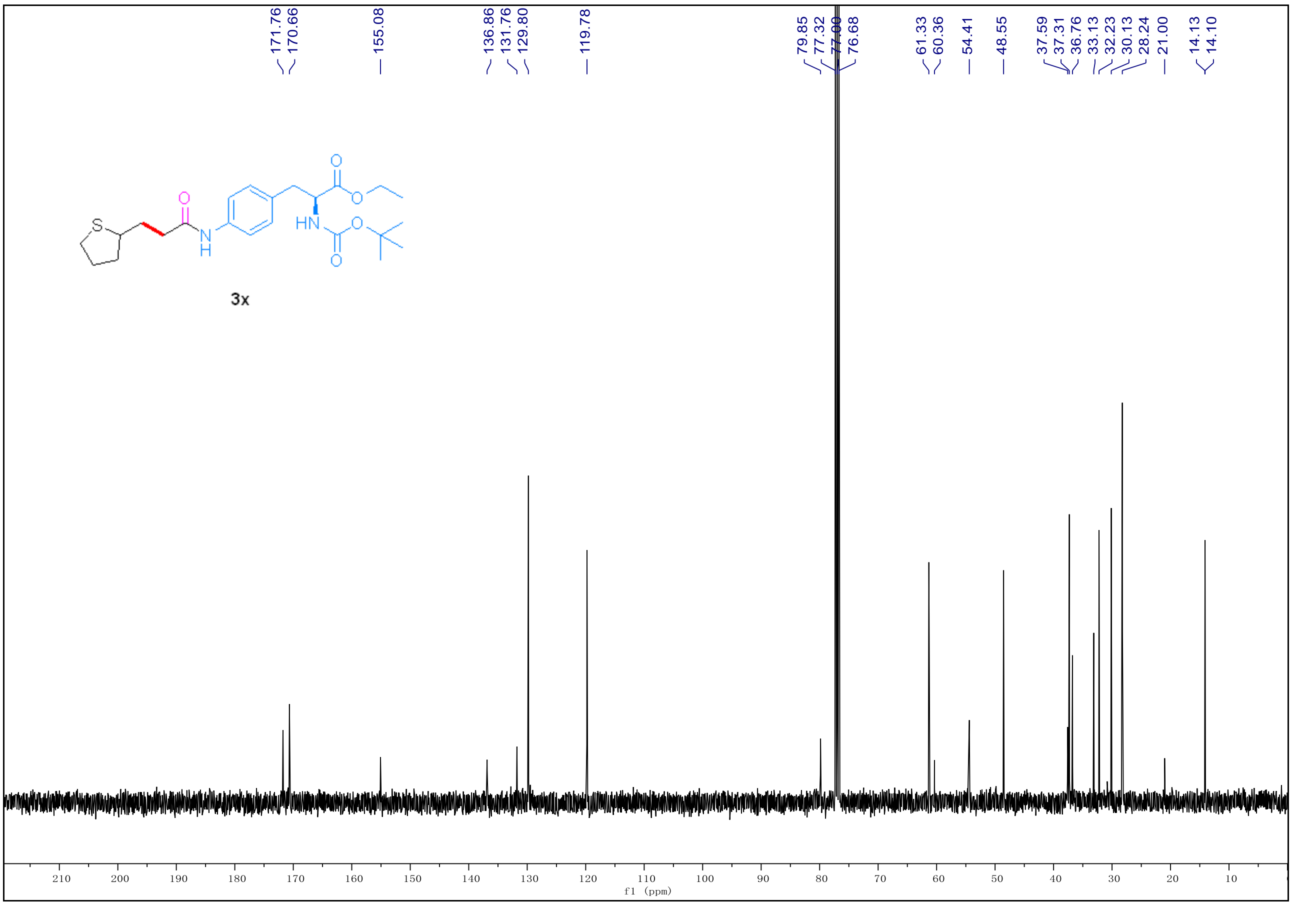

^{1}H NMR (700 MHz, $CDCl_3$) and ^{13}C NMR (175 MHz, $CDCl_3$) spectrum of 3y

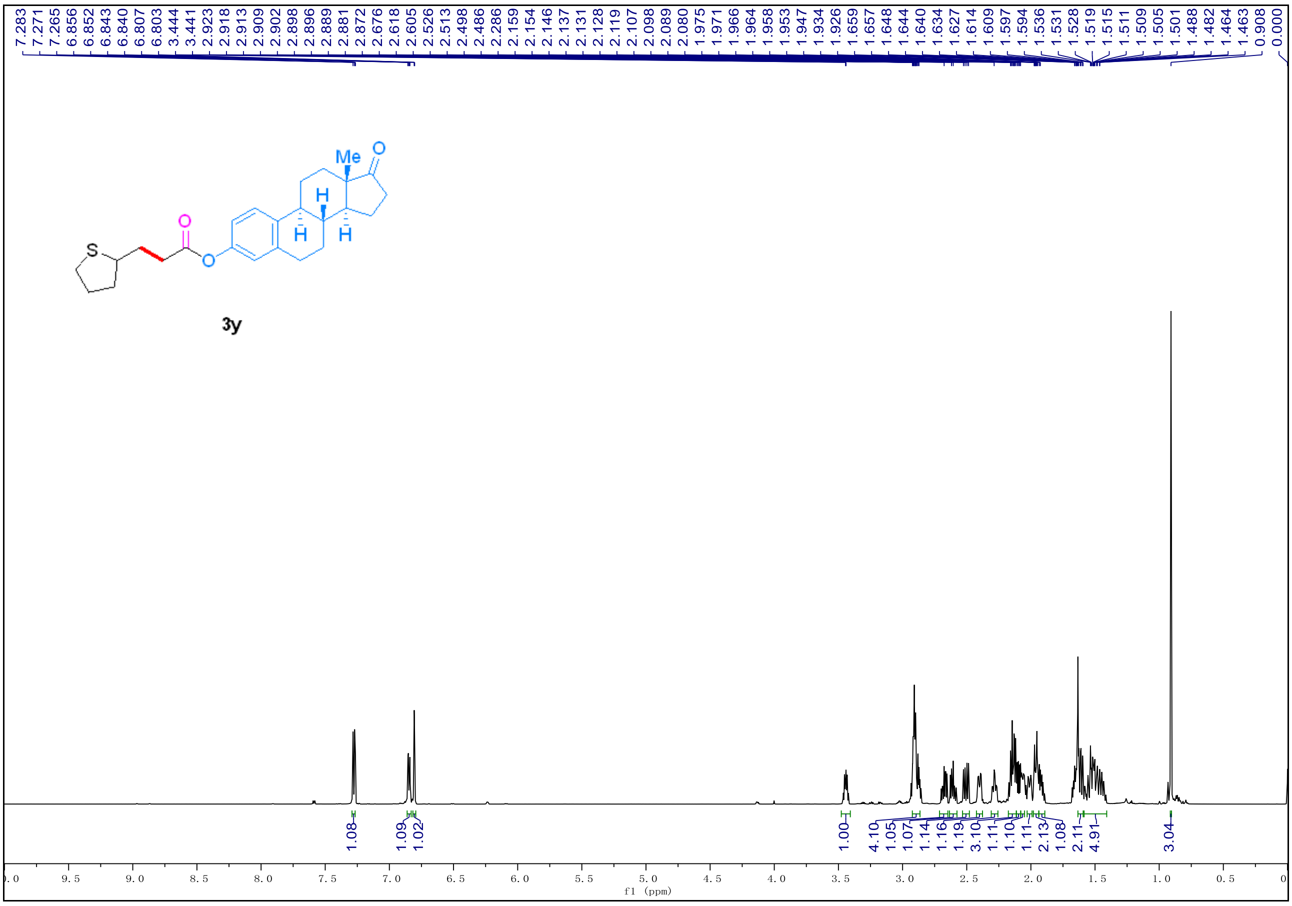

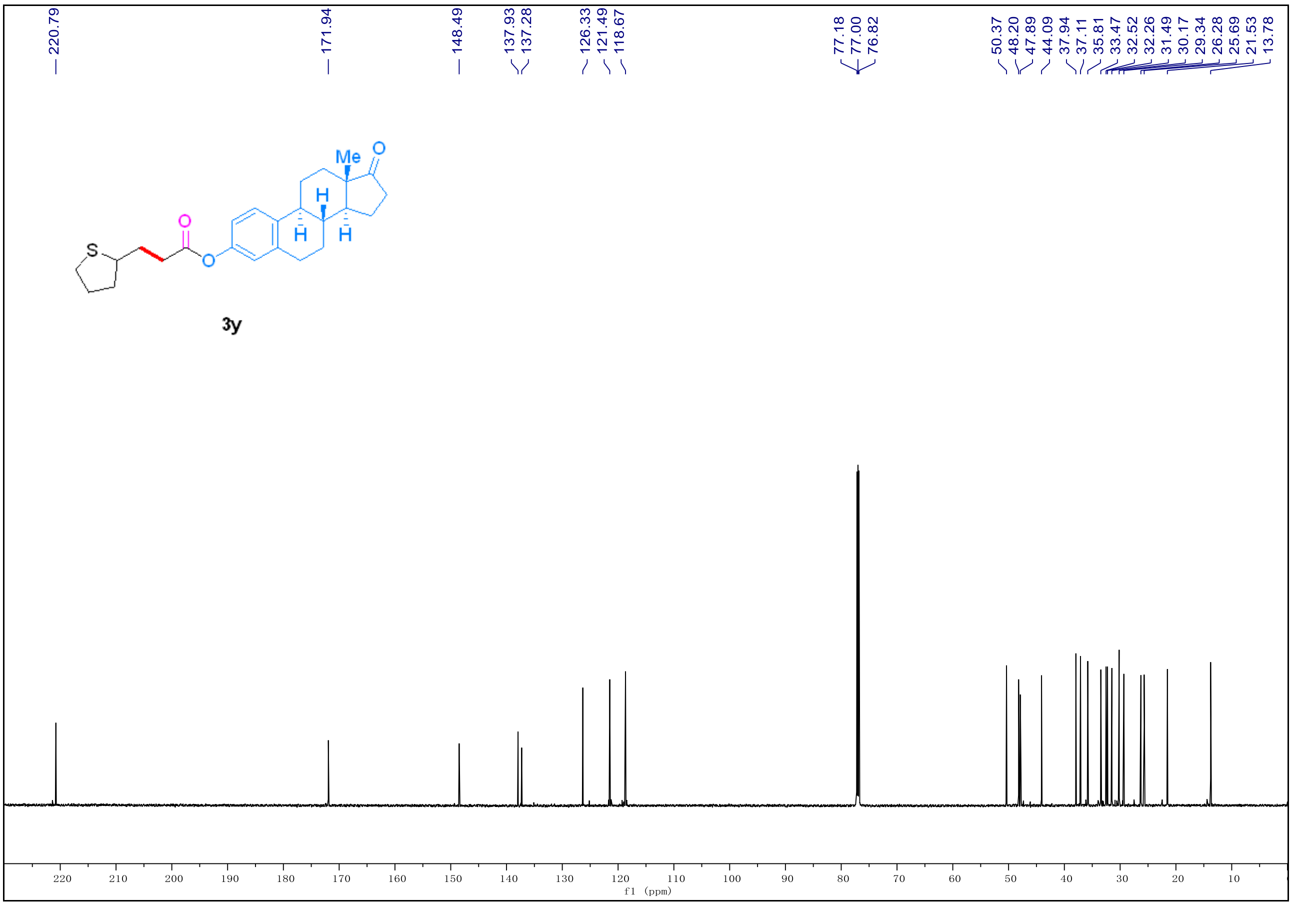

^{1}H NMR (400 MHz, $CDCl_3$) and ^{13}C NMR (175 MHz, $CDCl_3$) spectrum of 3z

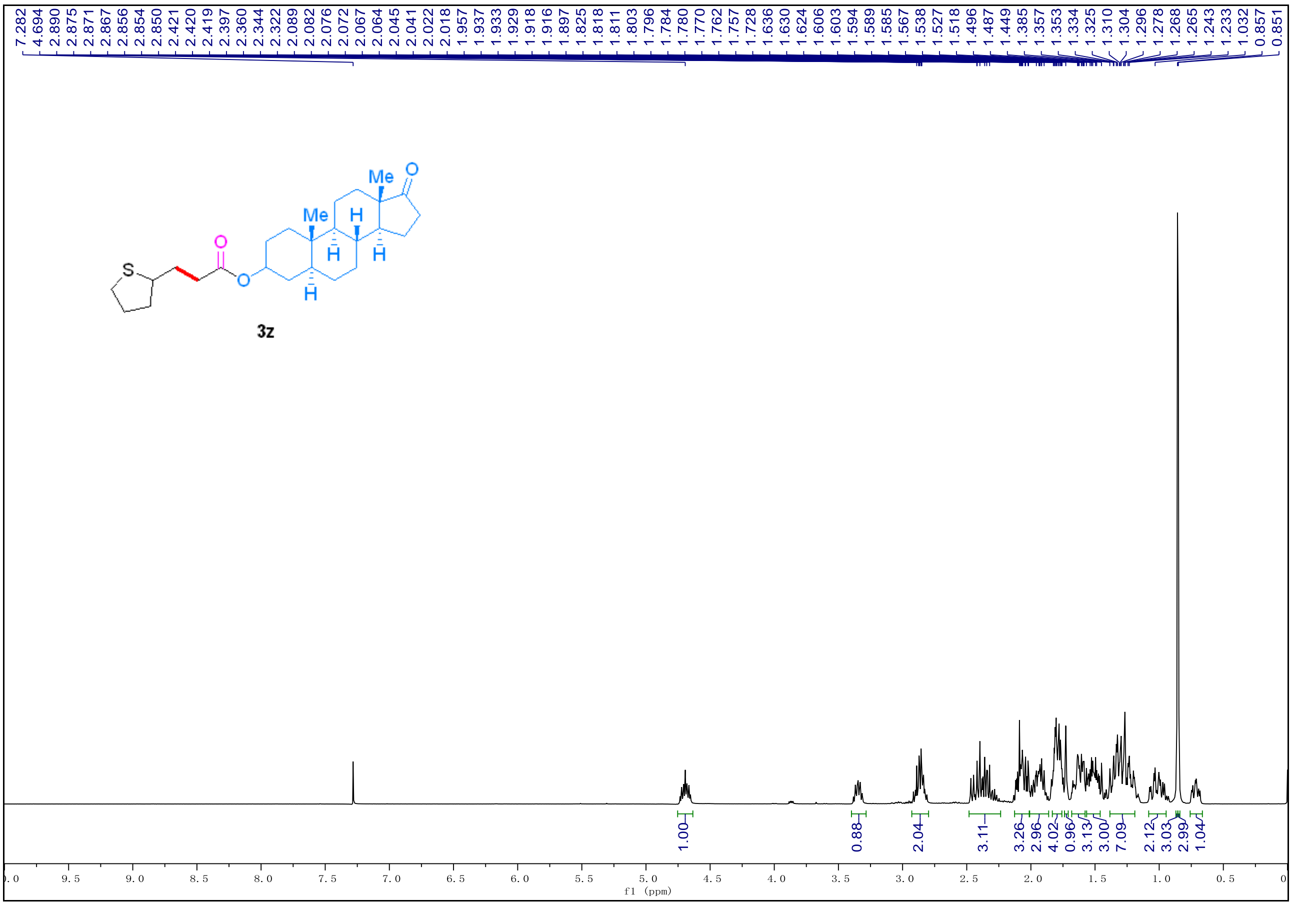

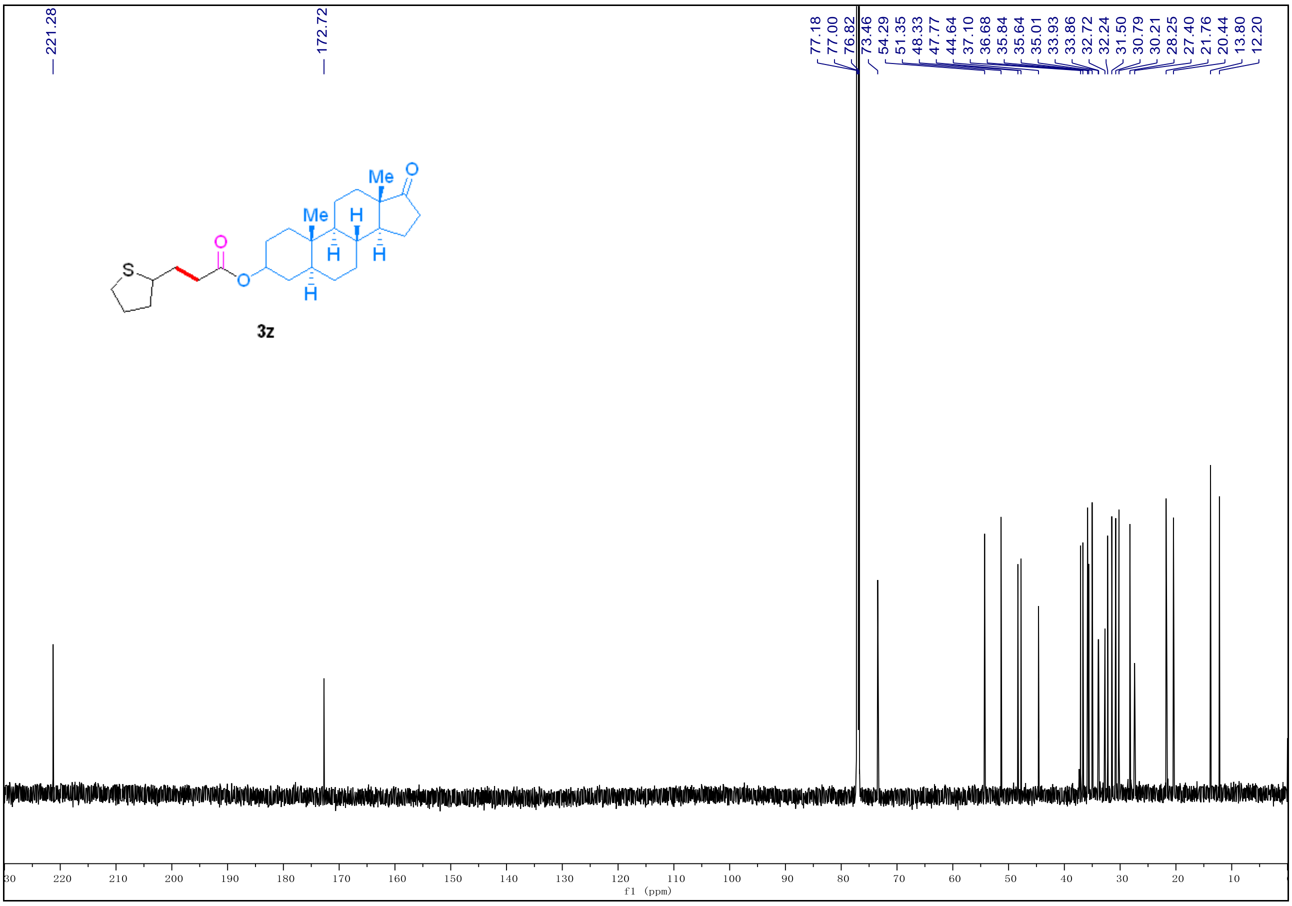

^{1}H NMR (400 MHz, $CDCl_3$) and ^{13}C NMR (100 MHz, $CDCl_3$) spectrum of 5a

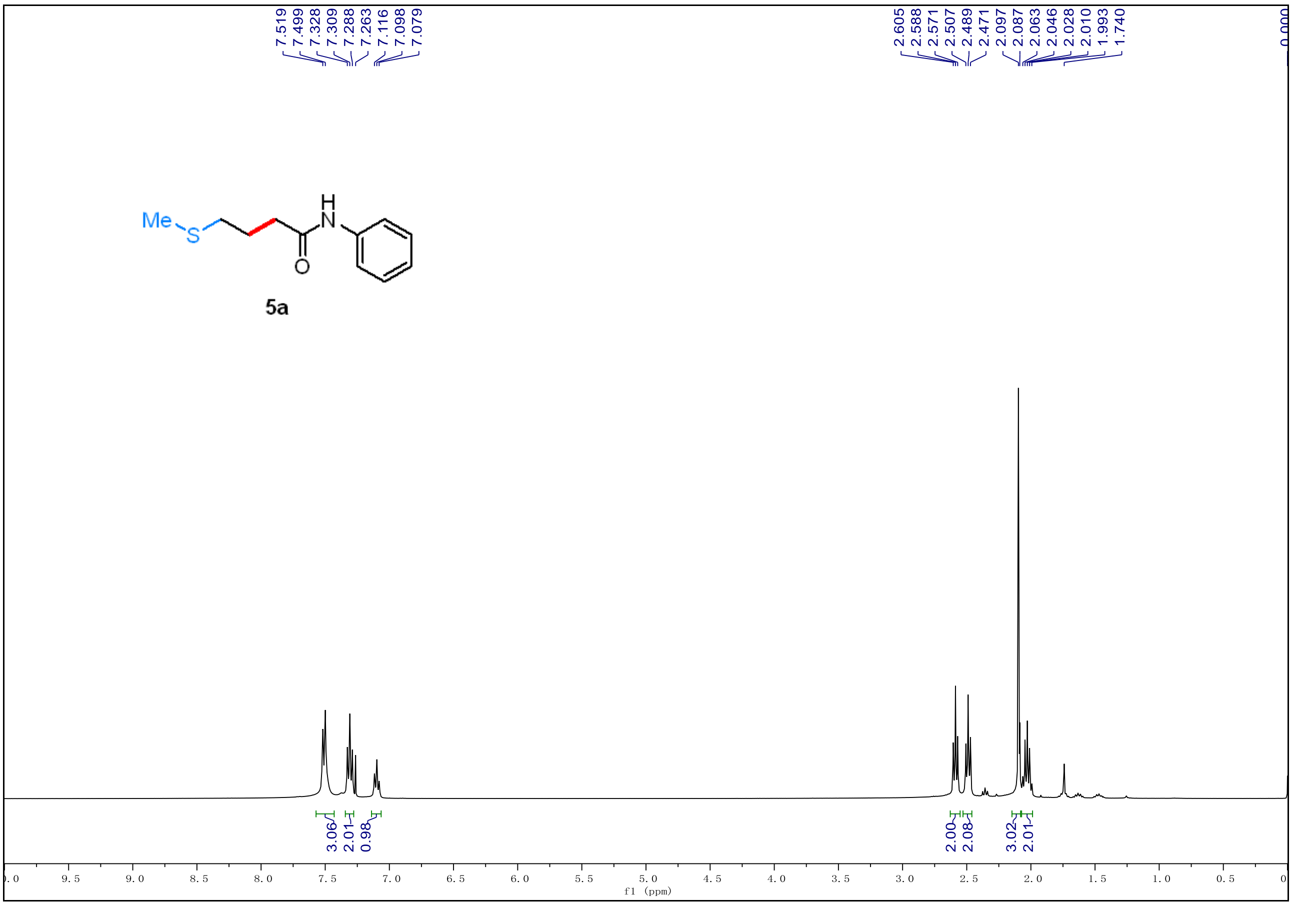

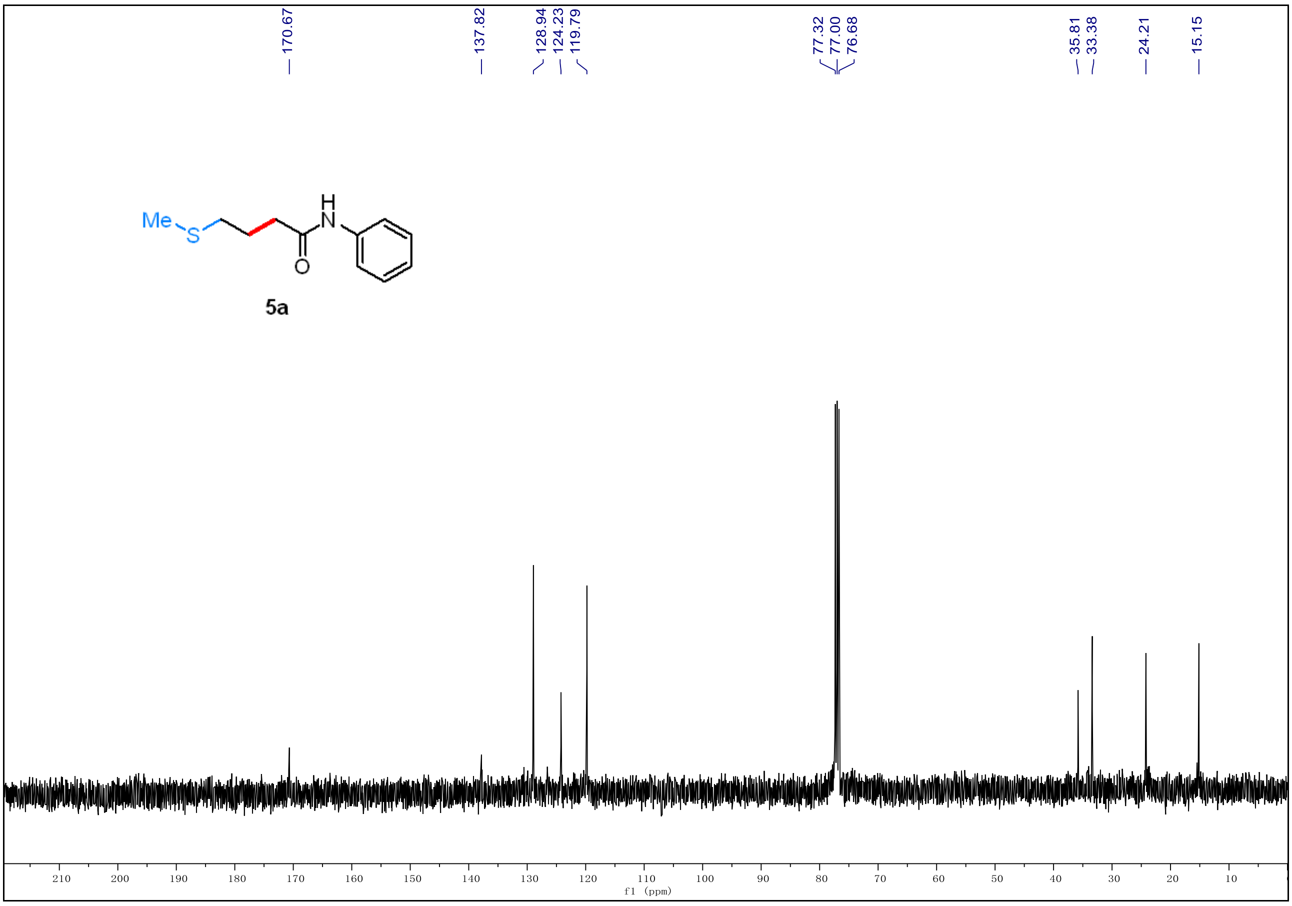

^{1}H NMR (400 MHz, CDCl₃) and ^{13}C NMR (100 MHz, CDCl₃) spectrum of 5b

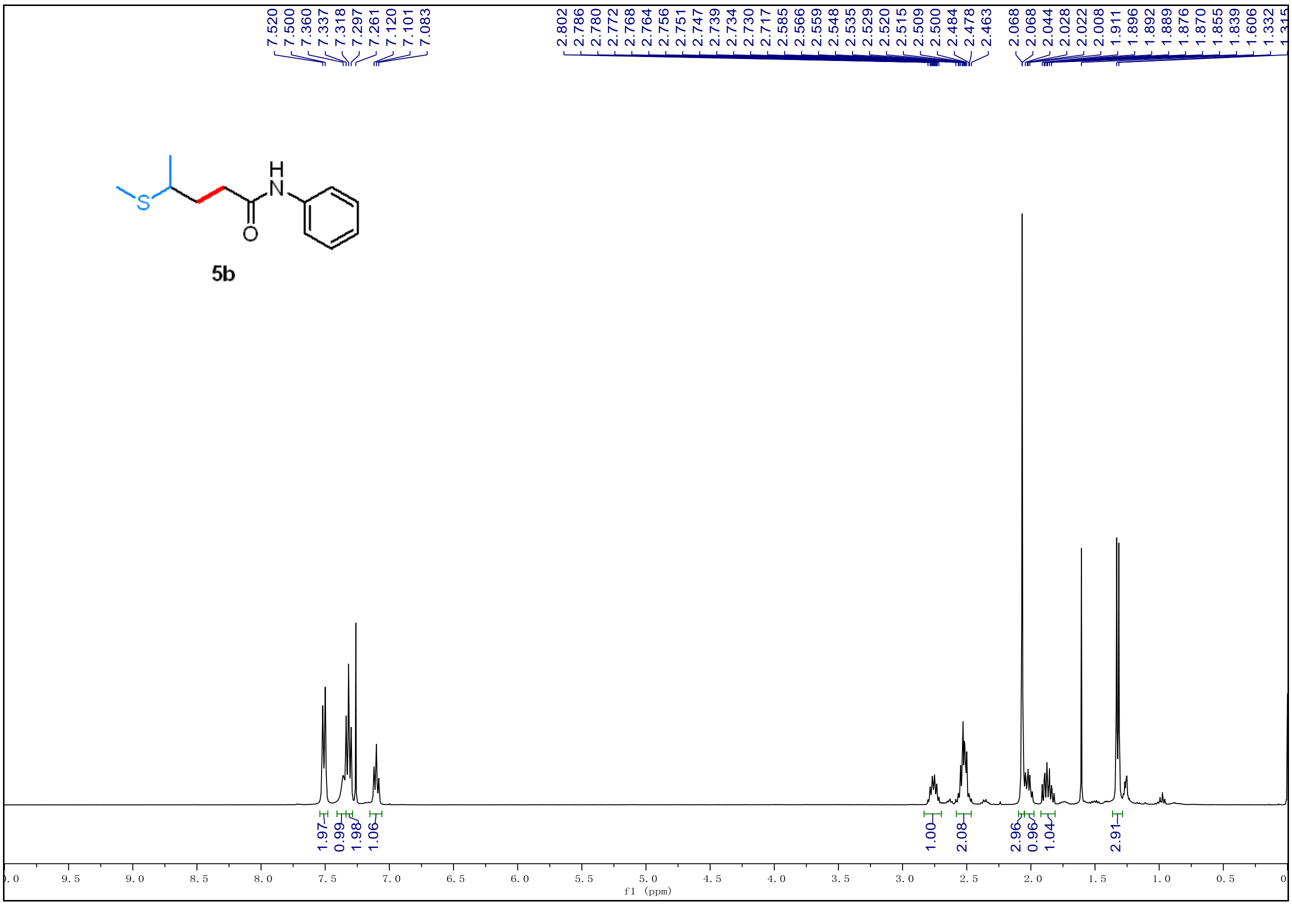

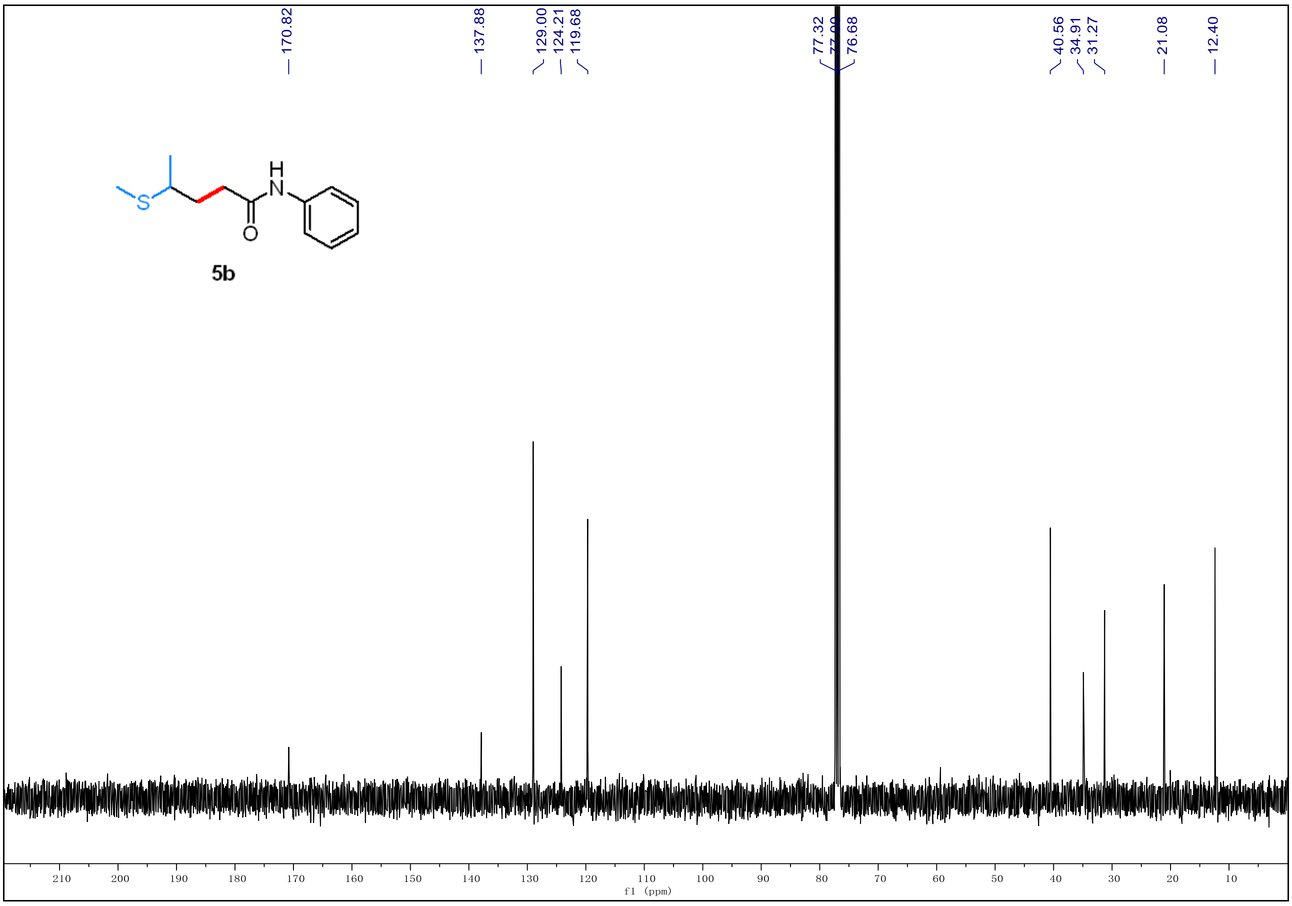

^{1}H NMR (400 MHz, $CDCl_3$) and ^{13}C NMR (100 MHz, $CDCl_3$) spectrum of 5c

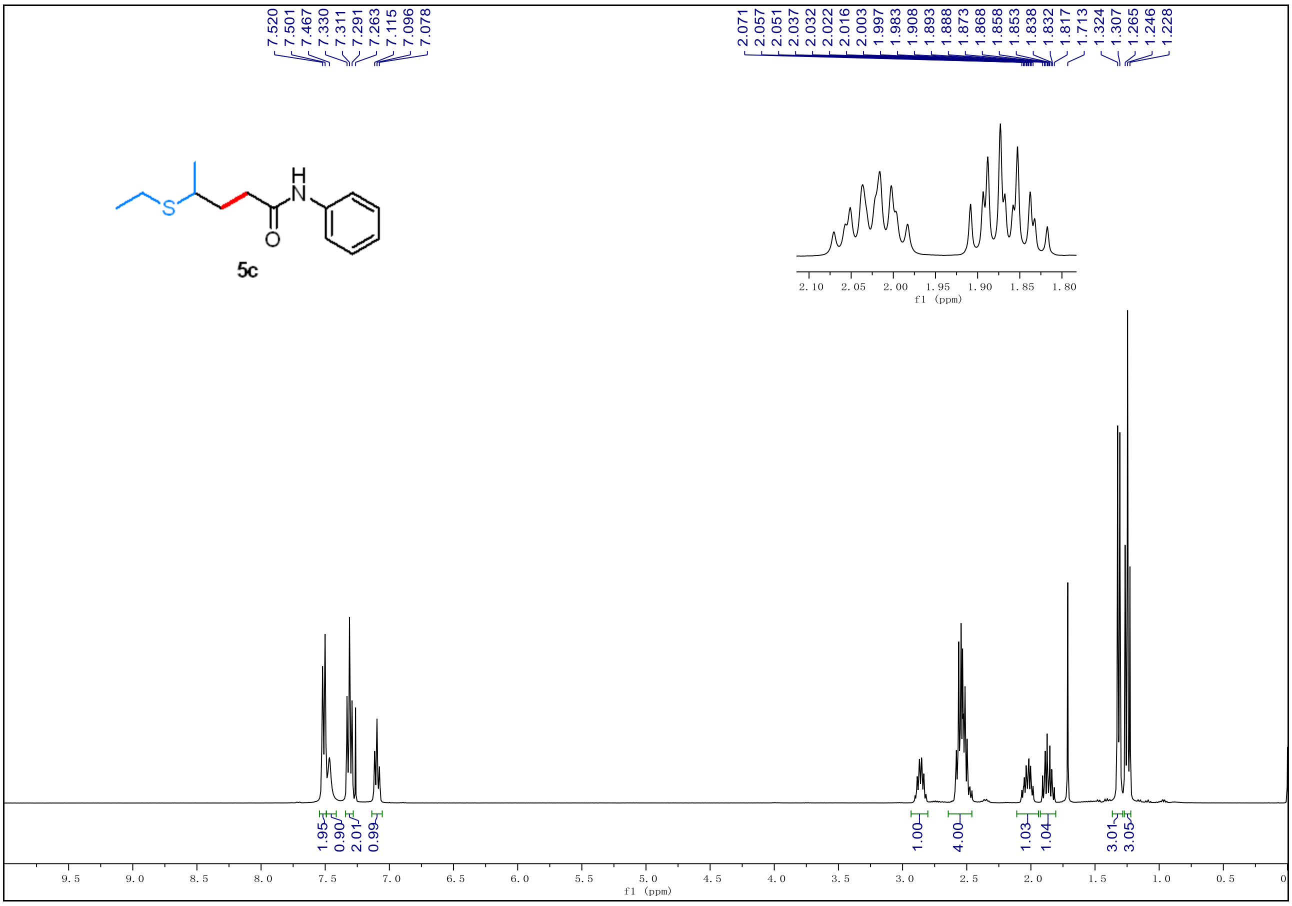

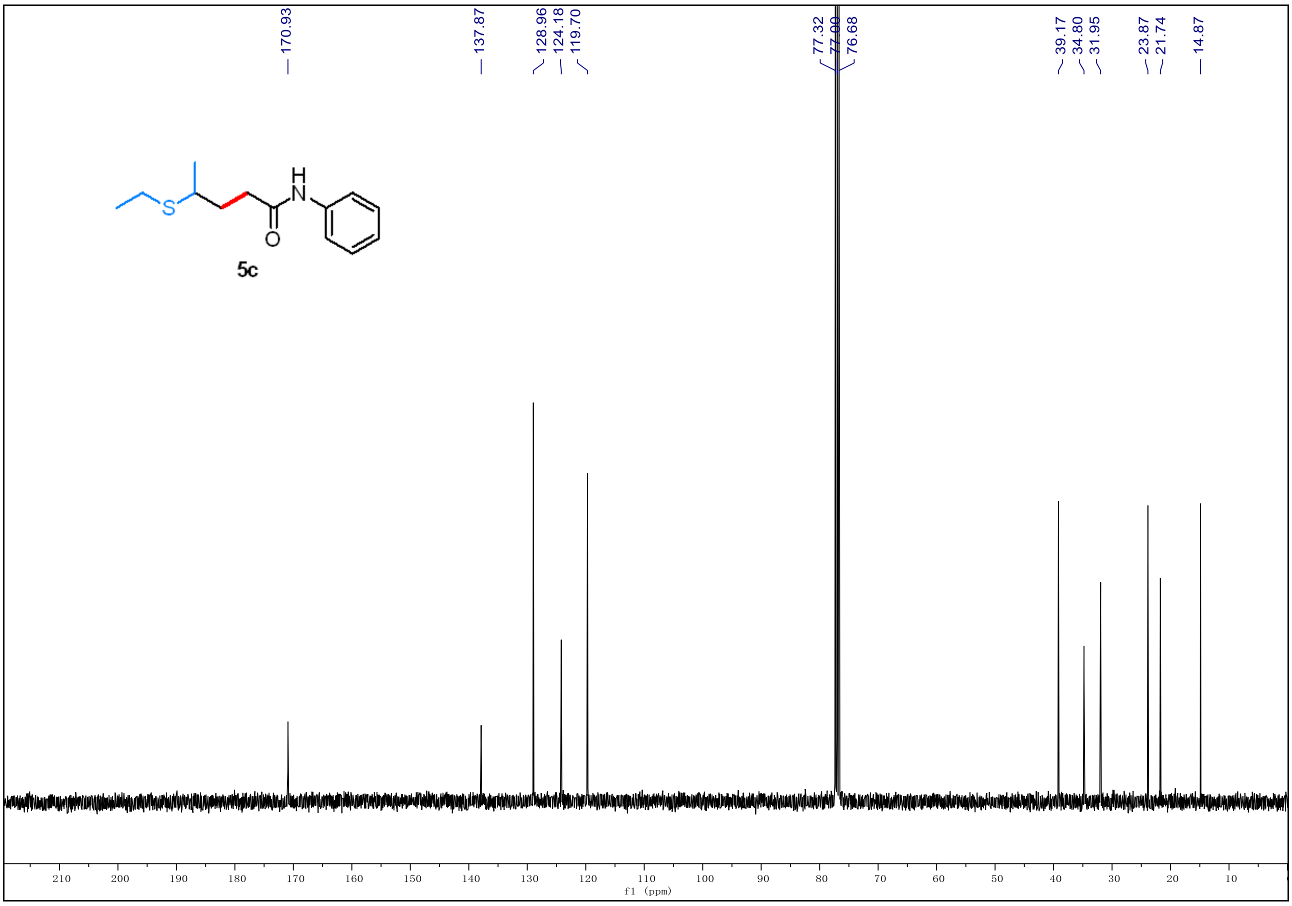

^{1}H NMR (400 MHz, $CDCl_3$) and ^{13}C NMR (100 MHz, $CDCl_3$) spectrum of 5d

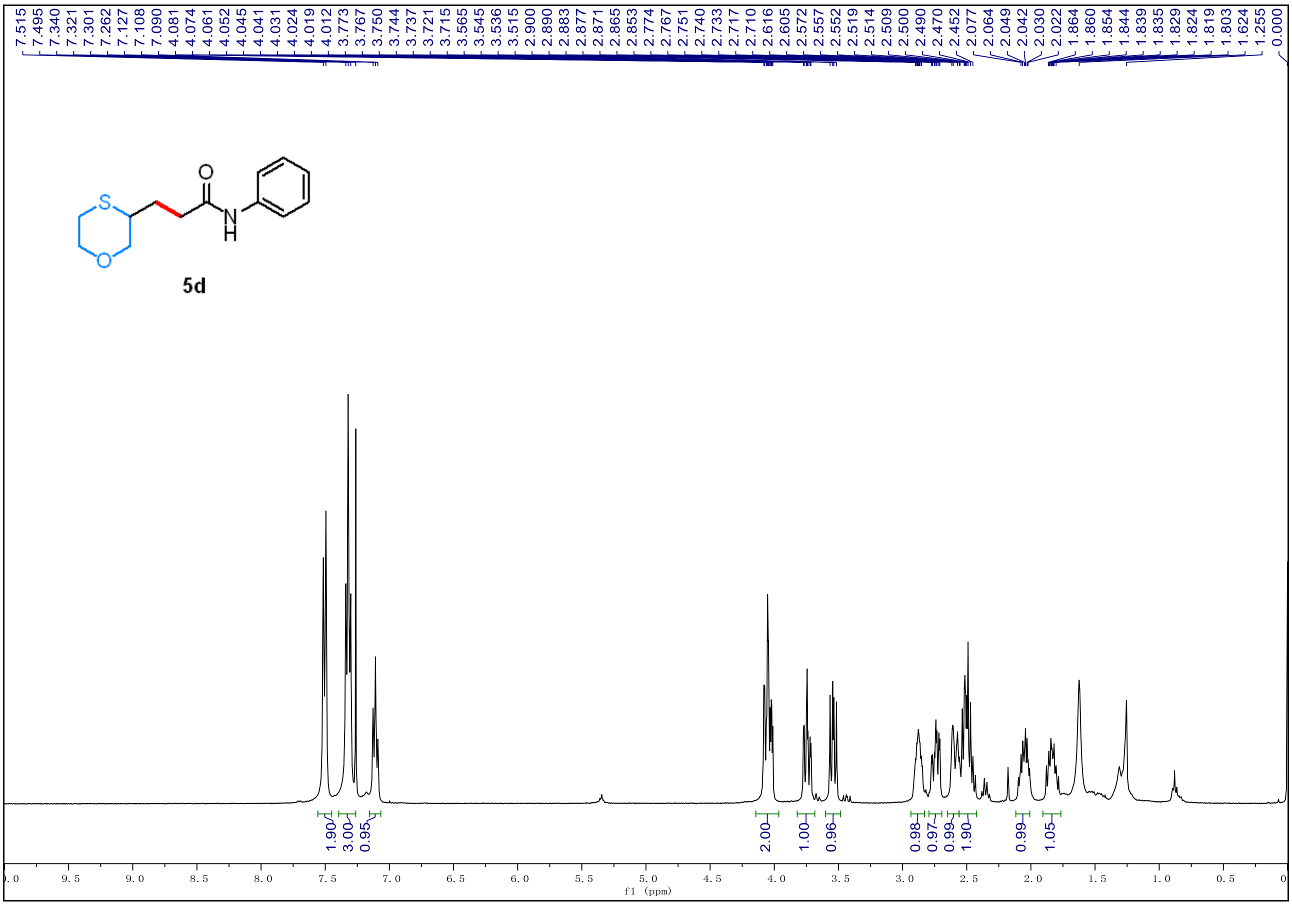

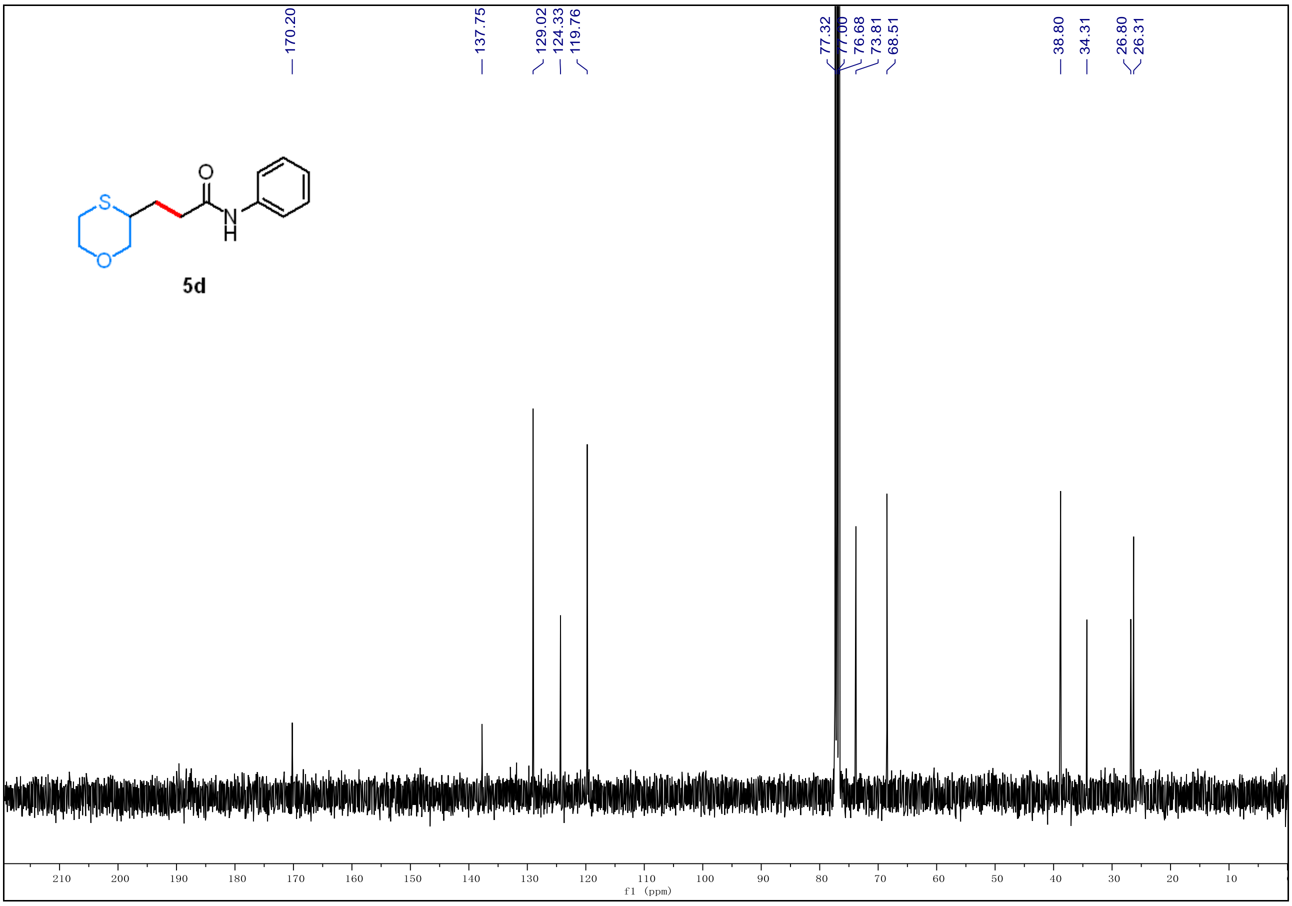

^{1}H NMR (400 MHz, CDCl3) and ^{13}C NMR (100 MHz, CDCl3) spectrum of 5e

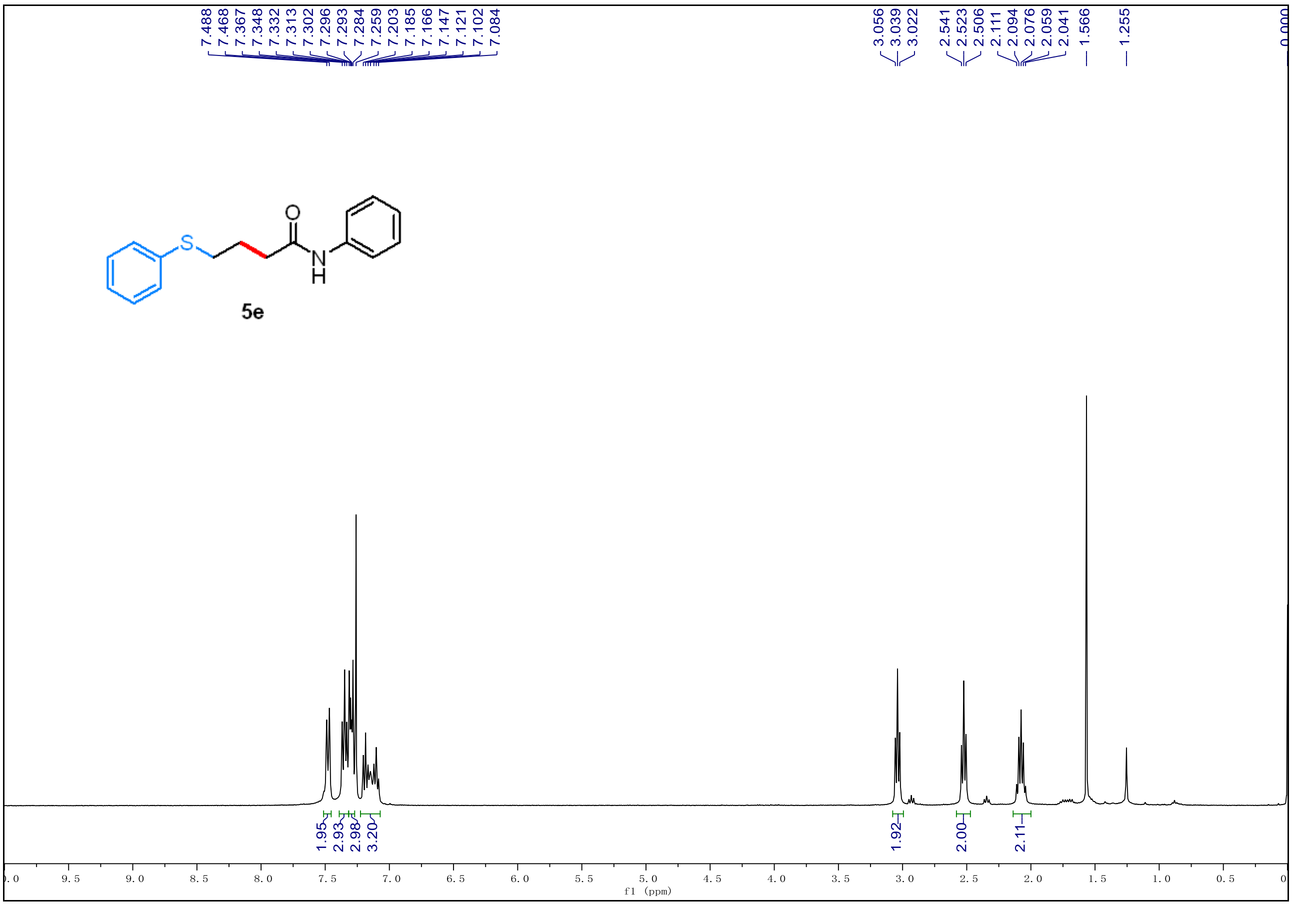

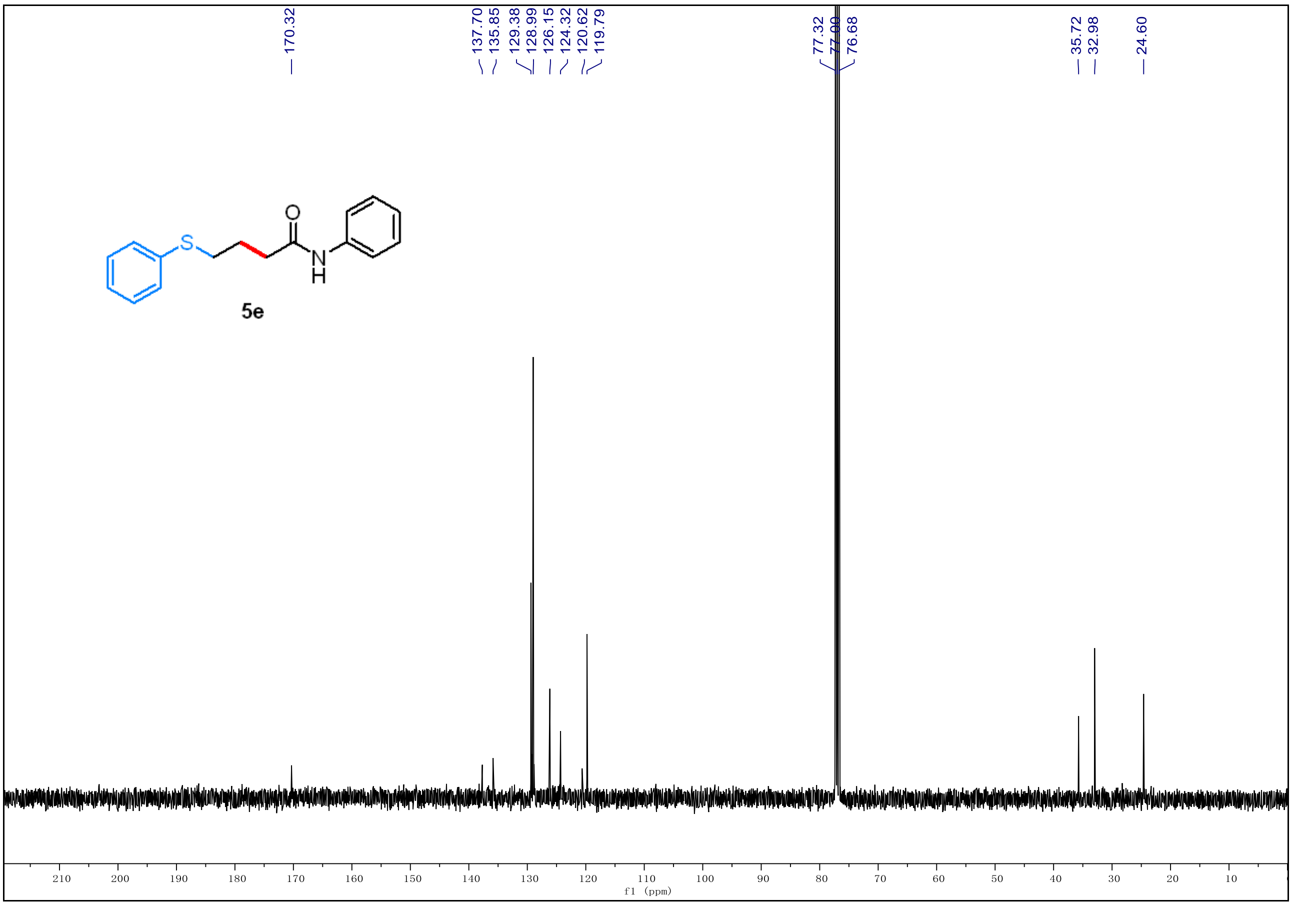

^{1}H NMR (400 MHz, CDCl3) and ^{13}C NMR (100 MHz, CDCl3) spectrum of 5f

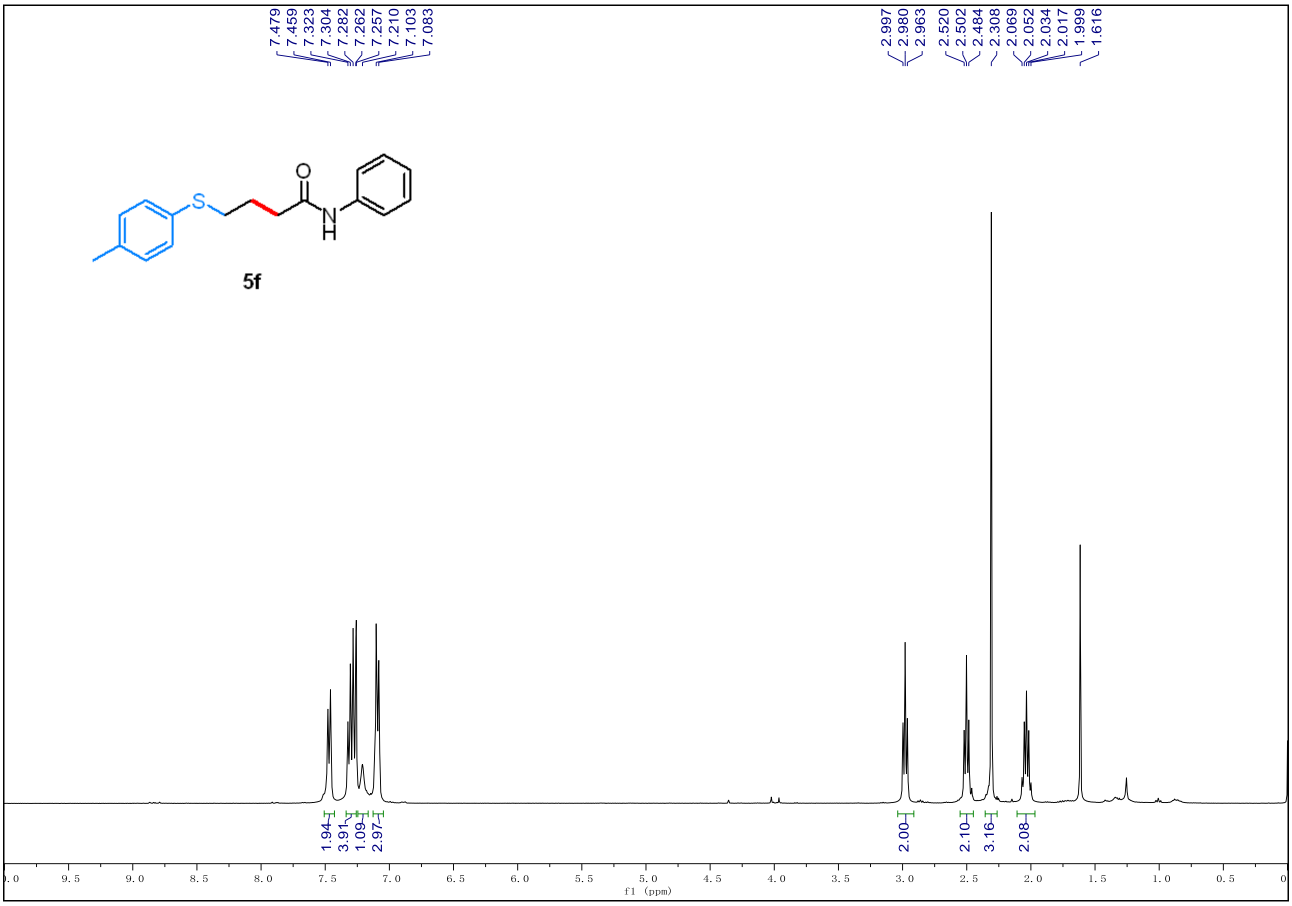

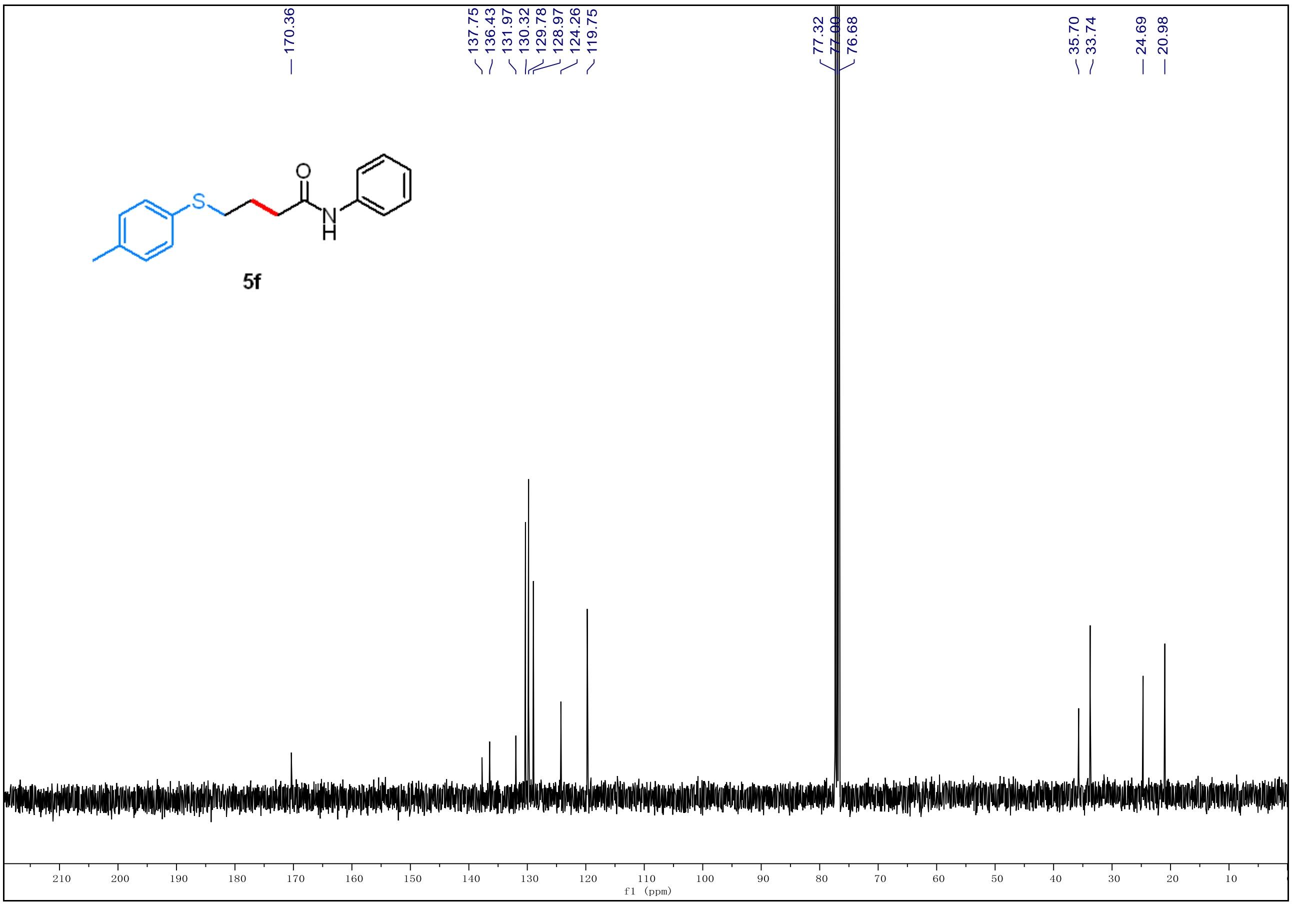

^{1}H NMR (400 MHz, $CDCl_3$) and ^{13}C NMR (100 MHz, $CDCl_3$) spectrum of 5f'

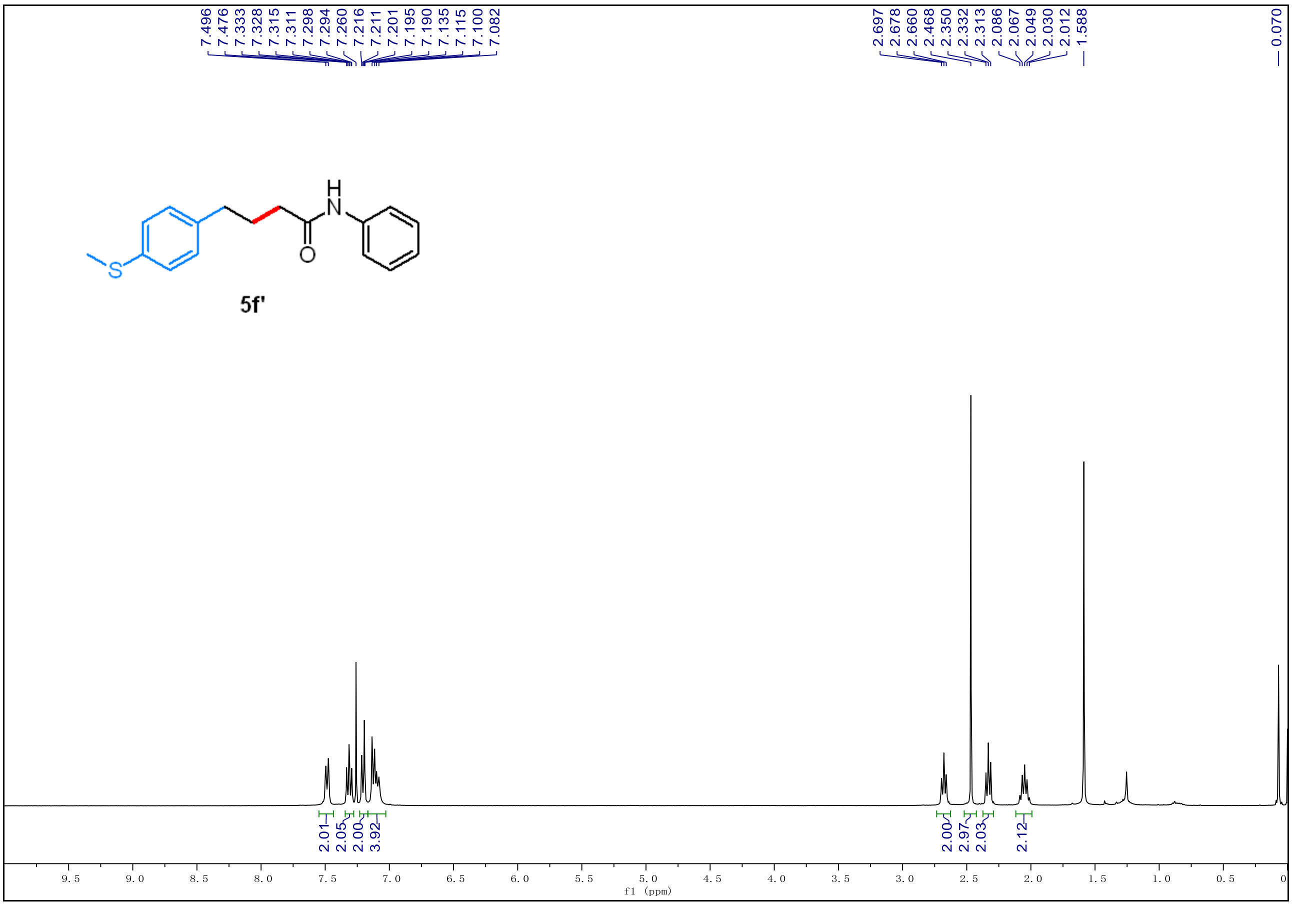

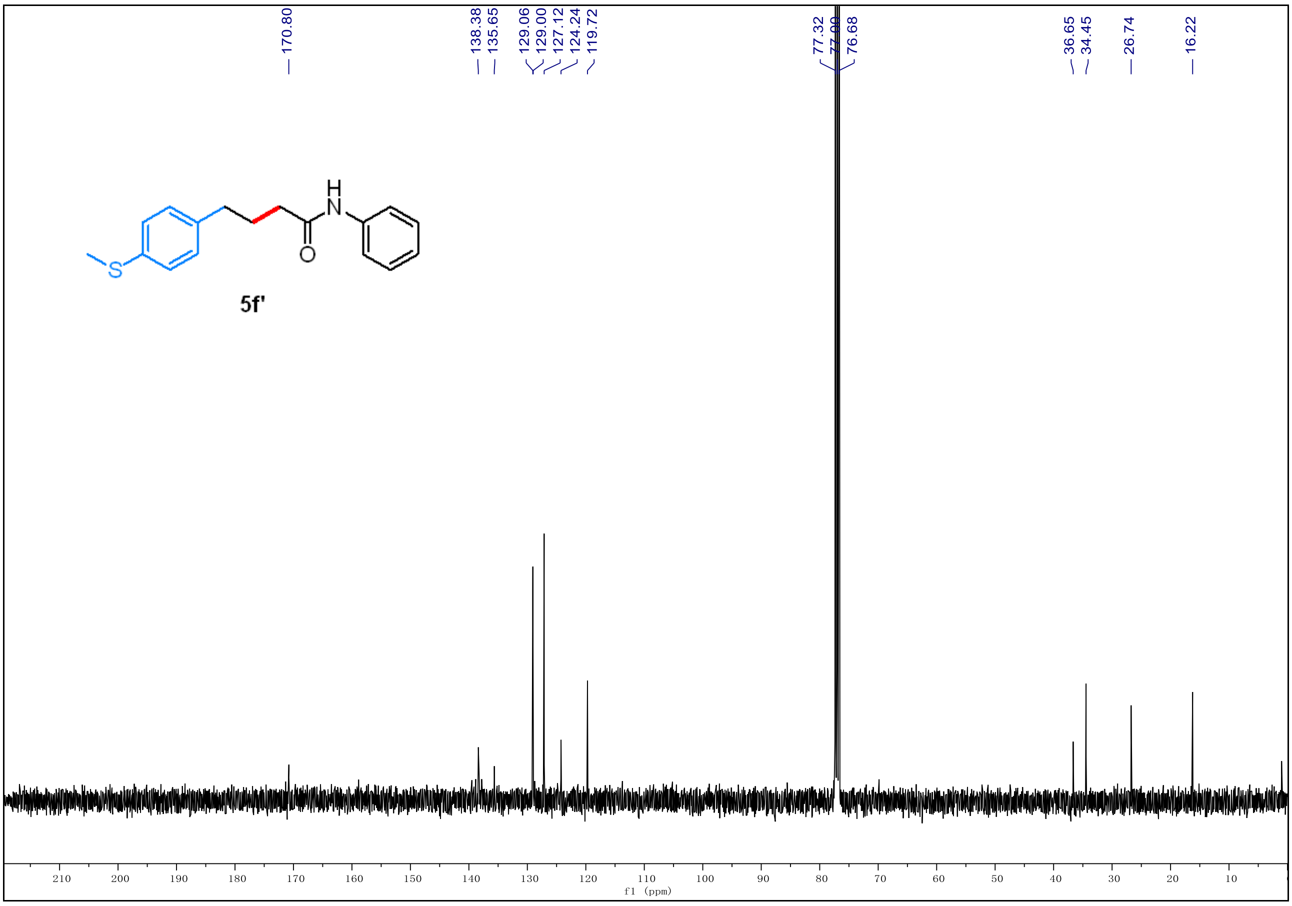

^{1}H NMR (700 MHz, $CDCl_3$) and ^{13}C NMR (175 MHz, $CDCl_3$) spectrum of 6

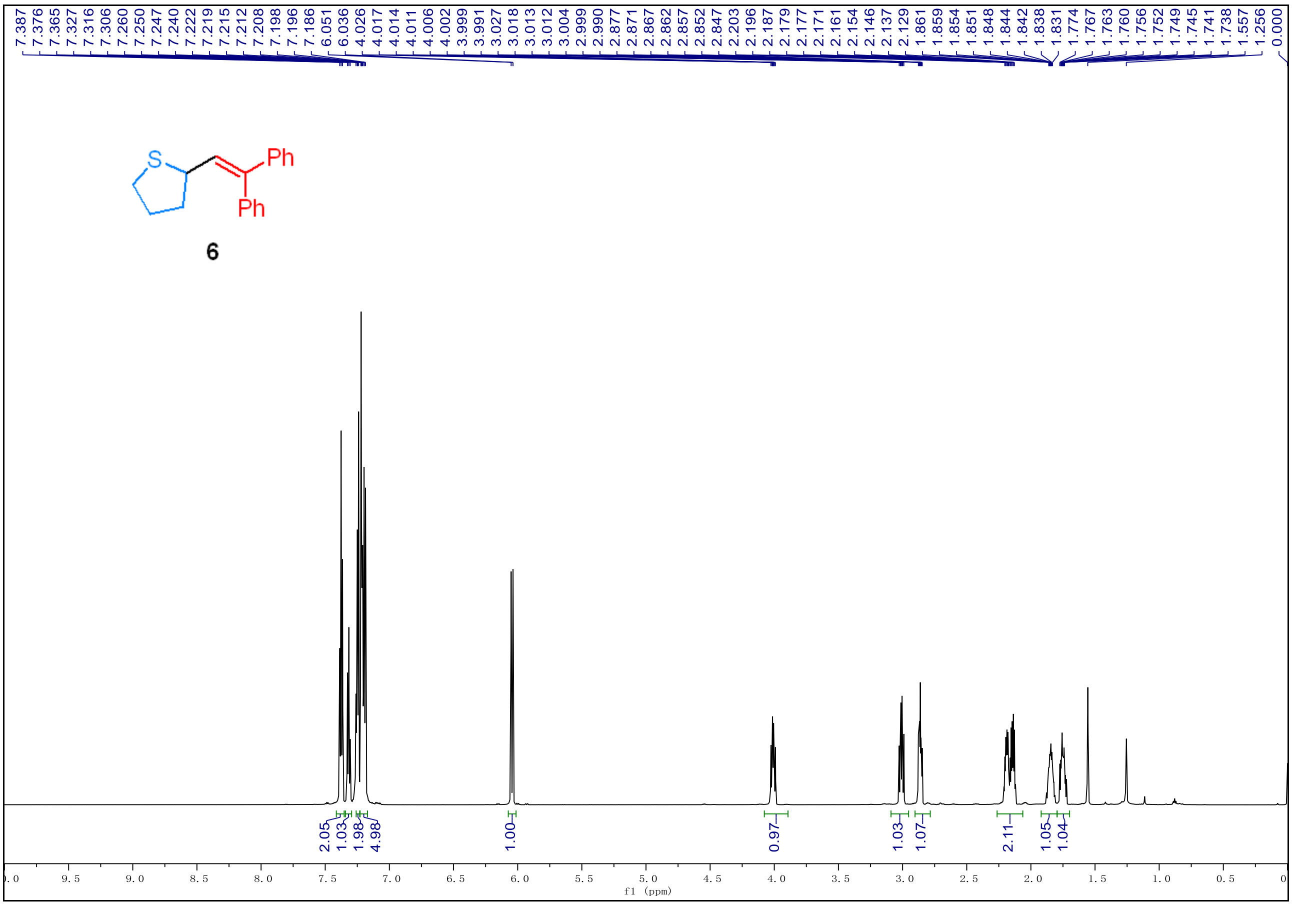

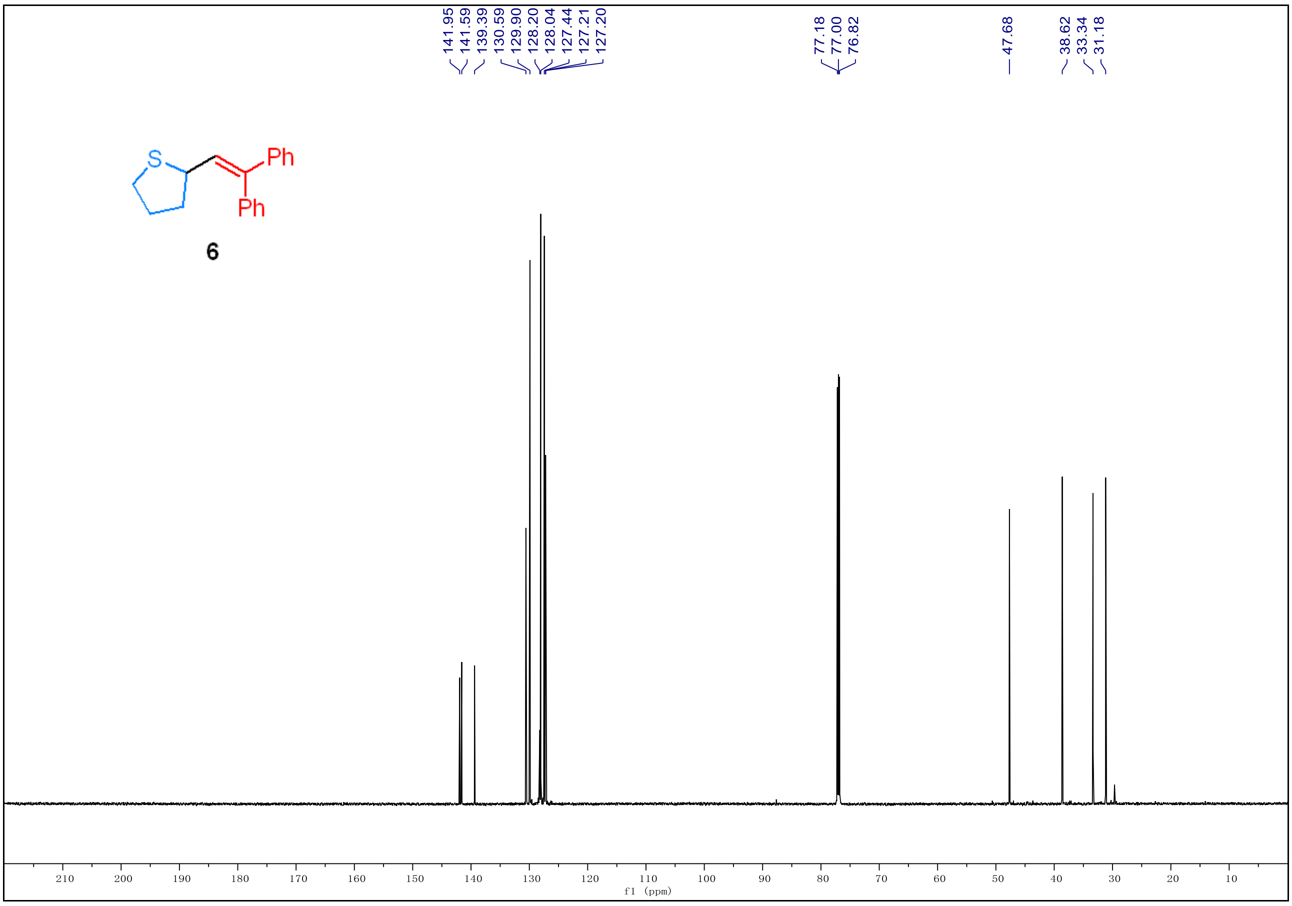

^{1}H NMR (700 MHz, $CDCl_3$) and ^{13}C NMR (175 MHz, $CDCl_3$) spectrum of 8

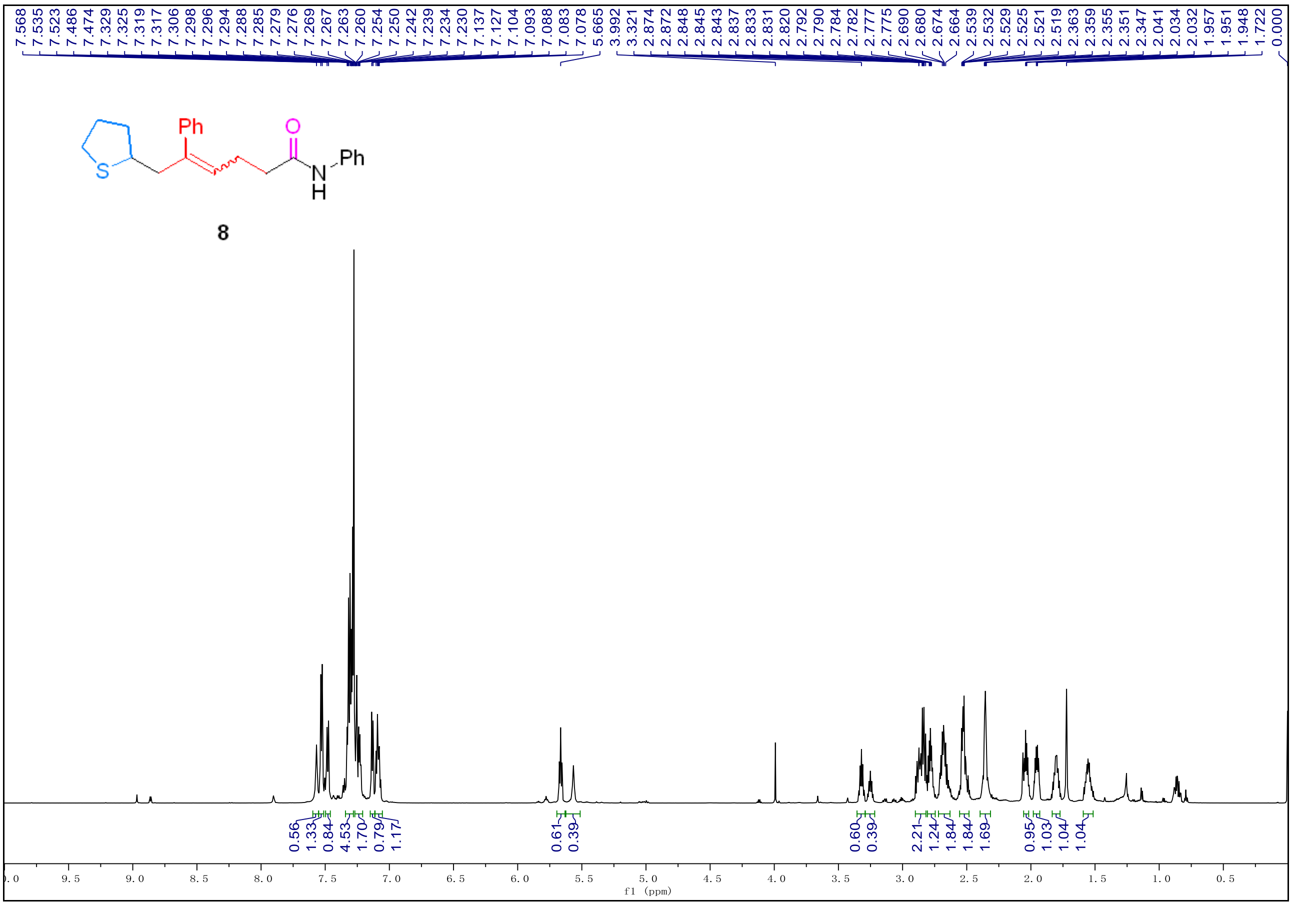

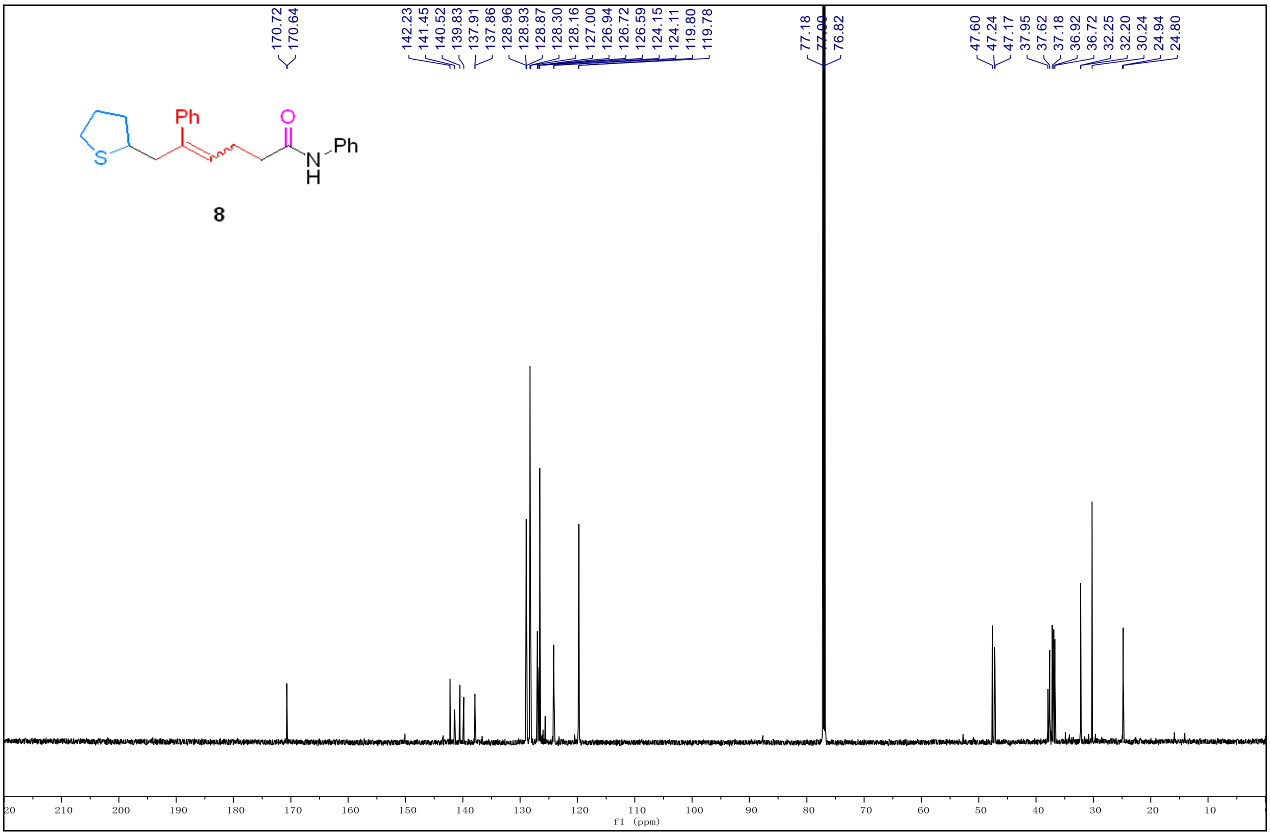

Supplement: Supplementary file 1 [file ol5c04357_si_001.pdf]
